# The pial vasculature of the mouse develops according to a sensory-independent program

**DOI:** 10.1038/s41598-018-27910-3

**Published:** 2018-06-29

**Authors:** Matthew D. Adams, Aaron T. Winder, Pablo Blinder, Patrick J. Drew

**Affiliations:** 1 Center for Neural Engineering, Department of Engineering Science and Mechanics, Pennsylvania State University, University Park, Pennsylvania, PA 16802 USA; 2 Neurobiology, Biochemistry and Biophysics School, George Wise Faculty of Life Sciences, Sagol School of Neuroscience, Tel-Aviv University, Tel-Aviv, 98888 Israel; 3 Department of Neurosurgery, Department of Biomedical Engineering, Pennsylvania State University, University Park, Pennsylvania, PA 16802 USA

## Abstract

The cerebral vasculature is organized to supply the brain’s metabolic needs. Sensory deprivation during the early postnatal period causes altered neural activity and lower metabolic demand. Neural activity is instructional for some aspects of vascular development, and deprivation causes changes in capillary density in the deprived brain region. However, it is not known if the pial arteriole network, which contains many leptomeningeal anastomoses (LMAs) that endow the network with redundancy against occlusions, is also affected by sensory deprivation. We quantified the effects of early-life sensory deprivation via whisker plucking on the densities of LMAs and penetrating arterioles (PAs) in anatomically-identified primary sensory regions (vibrissae cortex, forelimb/hindlimb cortex, visual cortex and auditory cortex) in mice. We found that the densities of penetrating arterioles were the same across cortical regions, though the hindlimb representation had a higher density of LMAs than other sensory regions. We found that the densities of PAs and LMAs, as well as quantitative measures of network topology, were not affected by sensory deprivation. Our results show that the postnatal development of the pial arterial network is robust to sensory deprivation.

## Introduction

Neural activity and development is energetically demanding^1–4^, and the glucose and oxygen that supply the needs of neurons and astrocytes must be supplied by blood flow^5,6^. The structure of the cerebral vasculature is a major determinant of the local metabolic supply^7,8^, but what aspects of the vascular structure is altered to match the neural demands is not well understood. Consistent with the structure of the vasculature being a key determinant of local metabolic supply, anatomical studies have shown that the capillary density is matched to the metabolic demand of the tissue^9,10^. On the coarse scale of areas and layers, vascular density is correlated with neural density and markers of metabolic demand^10–15^. This matching of the vascular structure and neural activity takes place during the postnatal period^16^. In rodents, during the four weeks following birth, both the cerebral vasculature^17–21^ and the cortical neural activity and connections^16,22–28^ undergo extensive modification and co-development. Perturbing neural activity with sensory deprivations (such as whisker plucking in rodent models) during the postnatal critical period (specifically in the first two postnatal weeks^29,30^) causes sustained reductions in metabolic activity^31,32^, cortical spiking rate following sensory stimulation^33^, excitatory and inhibitory synaptic strengths^34^, and also altered synaptic connectivity^22,35^.

Because of this co-development, disruptions of normal neural activity will drive changes in capillary density^21,36^, though this deprivation must be done during the early critical period for it to be effective (see^21^). Early life experience is known to bi-directionally modulate cortical capillary density^37^. Sensory deprivation, such as whisker plucking or lesioning of the vibrissae follicles, decreases neural activity and capillary density^38^ in the deprived region. As with synaptic plasticity^39^, there is a critical period where the vascular network is more malleable to these perturbations. In adulthood (approximately after postnatal day 30 in mice), the vascular networks are much less plastic^19,21,40,41^. The postnatal remodeling of the pial vasculature is primarily due to removal of leptomeningeal anastomoses (LMAs)^17,20,42^. LMAs are collateral connections between arteries on the pial surface that form ‘loops’ in the vascular network that allow blood flow to route around an occlusion in one arterial branch^43^. Note that the pial arterioles and veins are *not* connected to the dural vessels. Though these two vascular structures are physically adjacent, they form completely unconnected vascular networks^44,45^.

While many studies have looked at the effects of neural manipulations on the capillary network density, the effects of perturbed neural activity on the pial arterial network has not been investigated. We hypothesized that in addition to capillary networks being altered by sensory deprivation, pial arteriole structure might be altered as well. Though the pial arterioles are innervated by peripheral nerves^46^, neural activity in the deep layers of cortex drives dilation^47^ (and sometimes constriction^48^) of pial arterioles. This strong relationship between neural activity in the cortex and the pial arterioles is due to the electrical conduction of dilatory signal along the endothelial cells^49–54^. The net result of this strong electrical coupling between the parenchymal vessels and pial arterioles is that pial arterioles are strongly dilated by neural activity, evoked by sensory stimulation^55–59^, locomotion^44,54,60–62^, whisking^63^, or optogenetic stimulation of cortical neurons^64,65^. Whisker deprivation causes decrease in evoked firing rates during behavior and in response to whisker stimulation^66,67^. Acute sensory deprivation decreases metabolic activity in the deprived cortex in both humans^68^ and animals^31^. Sensory deprivation should consequently decrease the frequency and amplitude of pial arteriole dilation during the deprivation period, decreasing overall flow to the deprived region. Because of the conduction of electrical signals among arteries, these changes could spill over other brain regions. The pial arterioles are innervated by peripheral nerves^46^, though it is controversial whether they play a substantial role in controlling cerebral blood flow under normal physiological conditions^46,69^. Though pial vessels are in contact with the CSF, signals in the CSF do not seem to impact sensory-evoked dilation^70^. While we currently do not completely understand how the vascular system assembles and remodels, there is wealth of evidence that the amount and variations in blood flow control vessel growth or pruning^71–74^. Computer simulations have shown that networks of blood vessels instantiating flow-dependent remodeling or pruning can assemble into structures similar to those observed in animals^75–80^. Importantly, these simulations show it is not only the absolute level of flow, but also the spatial and temporal dynamics of the fluctuations in flow that shape the final vascular network. The existence and maintenance of the anastomoses in the network are particularly sensitive to flow variations^75–80^. Without fluctuations in flow, the anastomoses are pruned in these models. Whisker deprivation will change the spatial and temporal patterns of neural activity in vibrissa-related somatosensory cortex, which will consequently change the patterns of pial arteriole dilations, and thus flow, in the pial arterial network. These deprivation-induced flow changes could alter the pial architecture. While neurovascular coupling is weak or inverted in neonatal rodents before ~P15 and in human infants^6^, the effects of sensory deprivation at early time points will drive long-term alteration of neural activity and circuit function^22,33^, which will likely drive lesser increase in blood flow during later stages of the pial arterial remodeling period^17,20^. Other metabolic signals from astrocytes that not directly related to neural activity and phasic dilations may also play a role in shaping flow^81^.

Here, we examined how early life experience affected the number of penetrating arterioles and the topology of the pial arterial network in the cortex of mice. We unilaterally deprived mice of their whiskers for the first month of life, a manipulation that is known to decrease capillary density^36^, but does not alter the position or shape of the histologically visible cytochrome oxidase staining in barrel cortex^31,33^. By keeping this important anatomical landmark intact, we can unambiguously associate pial vasculature features with the underlying brain regions^54,57^. We then reconstructed the pial arteriole network of the middle cerebral artery (MCA) and nearby brain regions with respect to these anatomically identified regions, and quantified penetrating arteriole density, anastomoses density and quantified the network architecture in each brain region by calculating the number of vertices (bifurcations and penetrating arterioles) per vascular offshoot in primary sensory regions. As deprivation in one sensory modality can cause large scale reorganization of other sensory modalities and their corresponding cortical representations^82–87^, we performed our analyses not only in the vibrissae representation, but also in other identified primary sensory cortices (forelimb/hindlimb representation in somatosensory cortex, visual cortex, and auditory cortex). We found that deprivation did not change penetrating arteriole density, LMA density, or quantitative measures of network structure. However, we found that LMA density was higher in the hindlimb representation than other sensory areas. Our results show that the development of the pial arteriole network is robust to sensory deprivation.

## Results

**Figure 1:**
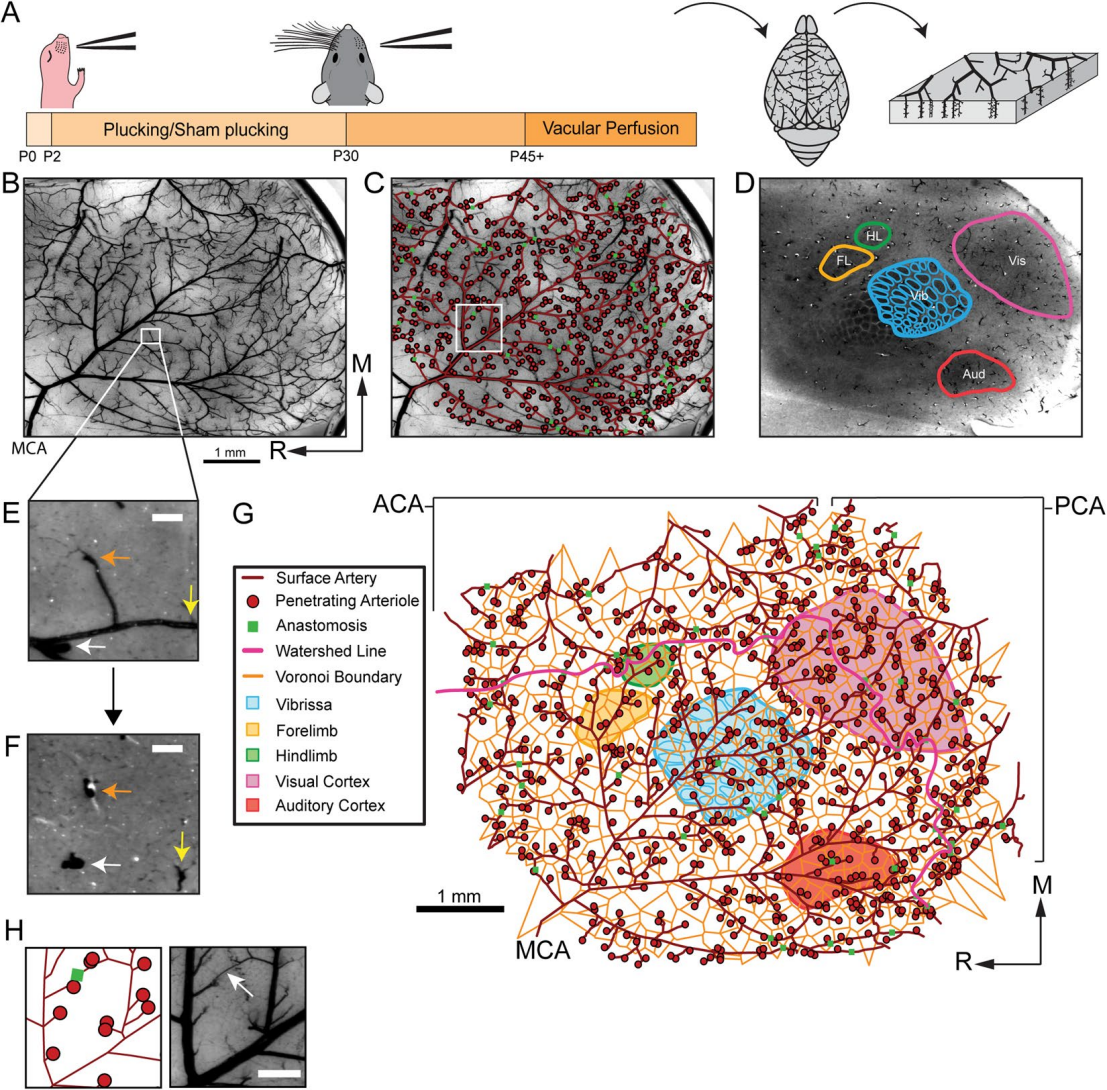
Reconstructing the pial arterial network with respect to the underlying neuroanatomy. (**A**) Schematic of experimental timeline. Whisker plucking/sham plucking was performed between P2 and P30, mice were sacrificed after P45. The vasculature was filled, the brain extracted, and the cortex flattened. (**B**) Photograph of a cortical slab. The Middle Cerebral Artery (MCA) is labeled in the bottom left corner. Scale bar: 1 mm. (**C**) The arterial vascular tracing overlaid on a picture of the slab vasculature. The arterial backbone is depicted in dark red. Penetrating arterioles (PAs) are depicted as red circles, and leptomeningeal anastomoses (LMAs) are shown as green squares. (**D**) Tangential section stained for CO, with S1 (vibrissae, forelimb, and hindlimb regions), visual, and auditory cortices denoted by colored lines. (**E**) Zoomed image of filled vasculature in (B). The white, orange, and yellow arrows point to penetrating arterioles (PAs). Scale bar: 100 µm. (**F**) A tangential slice taken just below the image in (E) demonstrating that a PA location can be verified by following it into the parenchyma. Colored arrows denote the same PAs noted in the previous image. (**G**) The completed pial arterial vascular reconstruction, including the anatomically-identified cortical regions, Voronoi cells centered around PAs (orange), and watershed line (pink) between the Anterior Cerebral Artery (ACA) and MCA, and Posterior Cerebral Artery (PCA) and MCA. Note that PAs and LMAs are both vessels, though they are denoted by point markers. Scale bar: 1 mm. (**H**) Zoomed image of anastomoses (box in C). Scale bar: 0.25 mm.

We reconstructed the middle cerebral artery (MCA), along with the locations of the underlying primary sensory cortices in the left hemispheres of 19 mice (9 whisker-plucked mice and 10 sham-plucked mice) (Fig. 1). The locations of penetrating arteriole (PA) were validated by following the vessels down into subsurface sections (Fig. 1E,F). We also traced out the boundary between the areas perfused by the MCA and those perfused by the ACA and PCA, known as the watershed line^88,89^. The reconstruction was aligned to primary sensory areas using cytochrome oxidase staining (Fig. 1G). We saw no obvious differences in the positions of the cytochrome oxidase-stained sensory regions between deprived and control animals, and there were no significant differences in the areas of any of the sensory regions between the two groups (Statistical Supplement, p.5). Pial arteriole network reconstructions are shown in Supplementary Data Set 1.

Because our data was nested and multiple measurements were made from the same animals, (e.g., PA counts in the barrel cortex) statistical tests that deal with these issues are required^90^. We used a generalized linear model (GLM) to deal with the correlations arising from nested data (see Methods and Statistical Supplement, p. 1). Generalized linear models account for correlated data without inflating Type I error rates or loss of statistical power (problems that accompany statistical comparison tests like ANOVA) by distinguishing between variance arising from within- and between-group factors^90^. In addition, GLMs are capable of dealing with non-normally distributed data, such as the count data obtained here. In a few animals, we were unable to reconstruct the pial network in every sensory region due to damage, and these regions were omitted from subsequent analyses. We made across-animal comparisons rather than comparisons within animals, as deprivation can affect the metabolic rate^91^ and activity in the side contralateral to the deprived cortex^92^, making comparisons across hemispheres difficult to interpret.

### Penetrating arteriole density is relatively constant across primary cortical areas, and is unaffected by sensory deprivation

Penetrating arterioles branch off surface arteries and enter perpendicularly into the cortex, and supply the blood for a roughly cortical-column-sized area^93^. Due to the lack of topological redundancy at this level, penetrating arterioles are bottlenecks for blood flow^94–96^. Unlike the capillary bed which forms a continuum^93,97^, relatively small changes in the structure of pial and penetrating arterioles could lead to large changes in the flow dynamics, as the pial vessel responses to neural activity do not always parallel the responses of penetrating arterioles^54,58^.

**Figure 2:**
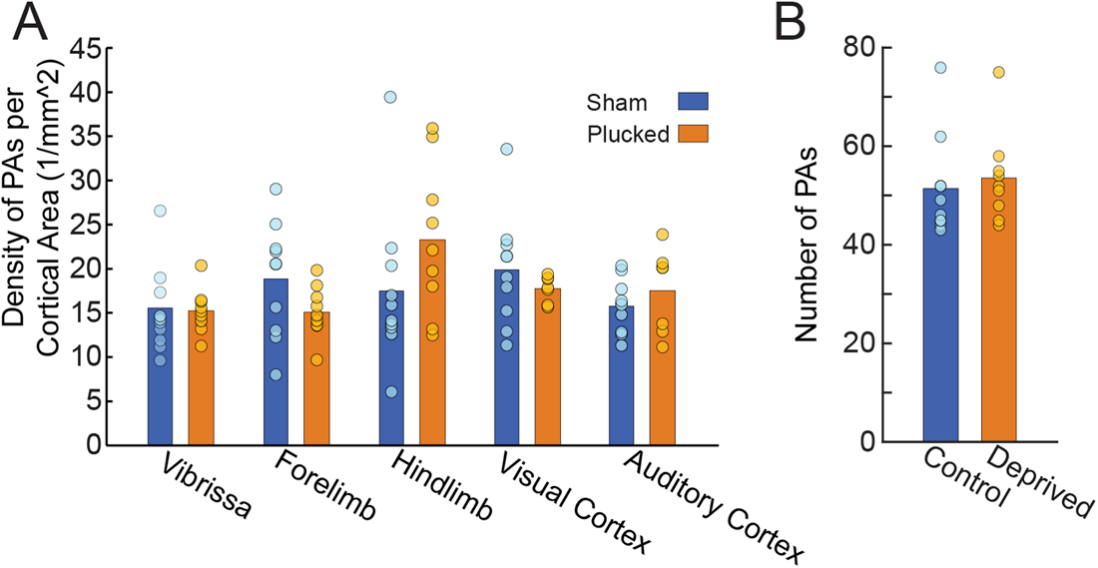
(**A**) Density of PAs plotted for five identified cortical areas (Sham: n = 10; Plucked: n = 9). Bars show mean across animals within a treatment (sham or deprived); circles show data points from individual animals. Color denotes group. (**B**) Comparison of the total number of PAs in plucked and control mice within the anatomically-identified barrel, forelimb and hindlimb areas. The PA count spans a factor of two in both conditions.

We first asked if whisker deprivation caused a reduction in the density of PAs for any cortical region (Fig. 2A). The average density of PA across all animals and cortical regions was 17.4 per mm^2^. Since the data were well represented by a Poisson distribution, the variance of the PA data among animals was roughly equal to the mean. Given previous measures showing high variability (>10 fold) in flux of blood through individual PAs^98–100^ and the tenfold range of velocities seen in individual capillaries of awake mice^41^, these measurements are consistent with a relatively constant global cerebral blood flow across animals that is partitioned amongst the variable number of vessels, producing the observed heterogeneity in flow. We determined that the PA density was nearly uniform across the analyzed cortical regions within single animals, although we did observe a small overall difference in PA density between the visual and barrel cortices (visual area PA density – barrels area PA density = 1.22 PA/mm^2^, p < 0.001, Z = 4.413, Tukey HSD, see Statistics Supplement, p. 28). Neither the mean PA density (p = 0.71, χ^2^(1) = 0.13) nor the region-specific PA densities (p = 0.10, χ^2^(4) = 7.87) were significantly affected by sensory deprivation (Likelihood ratio test, see Methods). These results show that, unlike the effects of sensory deprivation on capillary density^36^, PA density was robust to sensory deprivation.

### The density of leptomeningeal anastomoses was higher in the hindlimb region, and was unaffected by sensory deprivation

The pial arterial network has numerous interconnected loops or leptomeninigeal anastomoses (LMAs)^98,99,101,102^ that endow the network with a robustness against occlusions^43,99^. These LMAs are most prevalent in the watershed regions between the territories of major cerebral arteries and their corresponding perfusion territories^103^, but LMAs can be found anywhere in the arterial network. The location of this watershed region in humans is highly variable^104–106^, and differs across mouse strains^88,107,108^. Theoretical studies have shown that fluctuations in flow can generate and stabilize these anastomoses^76,79^. The number of anastomoses has a large impact on blood flow redistribution after stroke^88,89^, and consequently are a contributor to stroke outcome. In the mouse, the pial arterial network undergoes a pruning in the first few weeks of life^17,20^, during the same critical period as neurons and the capillaries, but it is not known what role cortical neural activity plays in the development of the pial arterial network.

**Figure 3:**
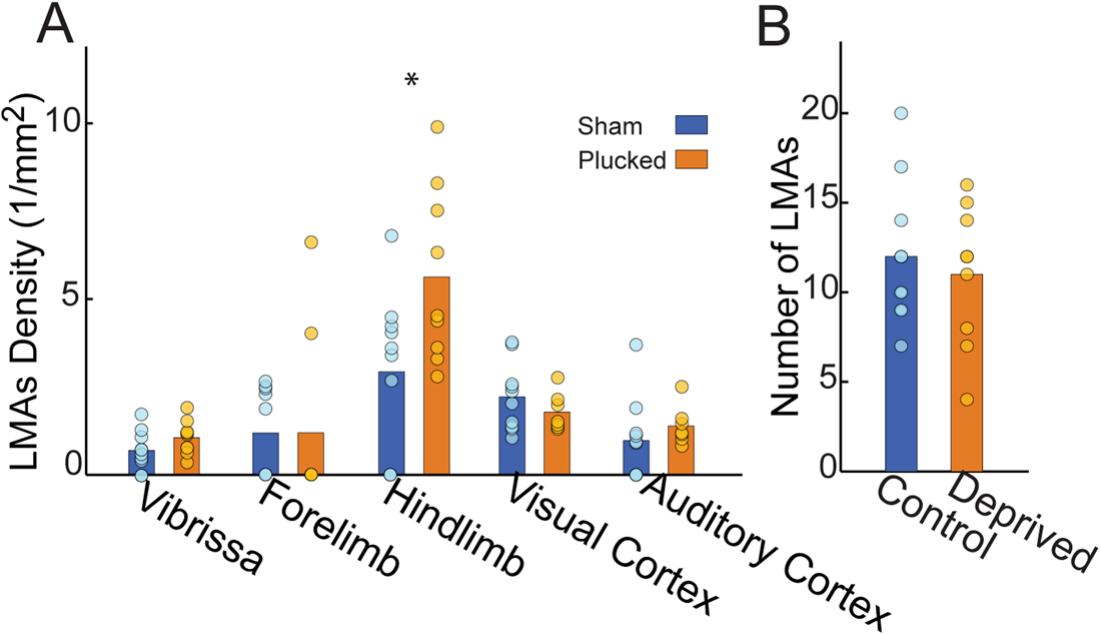
Lemtomeningeal anastomoses density was higher in the hindlimb representation of somatosensory cortex, but was not altered by sensory deprivation. (**A**) Density of LMAs for five cortical sensory areas. (**B**) Comparison of the total number of LMAs in sham-treated and plucked mice within the anatomically-identified barrel, forelimb, hindlimb areas. The LMA count spans a factor of three. Bars show means across animals within a treatment group, circles are data points from individual animals.

We asked if sensory deprivation altered the density of LMAs in identified cortical sensory regions (Fig. 3A). As with the total number of PAs, we observed wide variations in the number of LMAs across animals (approximately three-fold, Fig. 3B). This observed variance is consistent with previous measures of variability in LMAs being in both animals^88,99^ and humans^104–106^. We found that LMAs were significantly more likely to occur near the hindlimb region than auditory, visual, or barrel regions (see Statistical Supplement, p. 11). However, our results indicate that the number (p = 0.70, χ^2^(1) = 0.70) and location of LMAs in anatomically-identified primary sensory regions (p = 0.20, χ^2^(4) = 6.04) were insensitive to sensory deprivation (Likelihood ratio test, see Statistical Supplement, p. 15).

We also tested whether age at time of perfusion or sex had an impact on the variance in the number of PAs (Supplementary Fig. 1A) and LMAs (Supplementary Fig. 1B). We found that neither age nor sex significantly contribute to the observed variability (male: 253 ± 43.8; female, 308 ± 66.0; likelihood ratio test, PAs: p = 0.14, χ^2^(1) = 2.17; see Statistical Supplement page 25; LMAs: p = 0.24, χ^2^(1) = 1.40) (see Statistical Supplement, p. 14). These results show the effects of age at time of perfusion and sex did not impact the PA and LMA density.

### Lack of relationship between LMA and PA numbers within the whisker and limb regions

**Figure 4:**
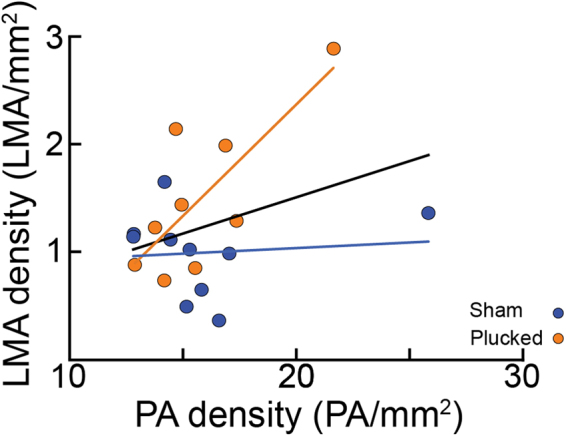
Relationship between number of LMAs and PAs. The number of PAs vs the number of LMAs within the barrel, forelimb, and hindlimb regions. While the number of PAs and LMAs trend together, the two vascular features were not significantly related (Linear regression fit by least squares, pooled data: p > 0.42, Bonferroni corrected, t(17) = 1.54; plucked: p = 0.051, Bonferroni corrected, t(7) = 3.10; sham: p > 0.79, Bonferroni corrected, t(8) = 0.28), suggesting that they develop independently. Each circle represents an individual mouse. Lines indicate linear fit.

As we saw a large variability in the number of PAs and LMAs across mice, we asked if these two features were correlated within the whisker and limb regions where we had the most complete reconstructions. If these two features co-varied, this would suggest they were regulated by a common process, or that the presence of one increased the numbers of the other. We found that the density of PAs was not significantly correlated with the number of LMAs (Fig. 4; pooled: y = 0.158 + 0.067x , p > 0.42 (Bonferroni corrected), R^2^ = 0.122; plucked: y = −1.774 + 0.207x , p = 0.051 (Bonferroni corrected), R^2^ = 0.578; sham: y = 1.00–0.057x, p > 0.675 (Bonferroni corrected), R^2^ = 0.23; least-squares regression; see Statistical Supplement, p. 6). The lack of a correlation between LMA and PA density suggests that independent processes drive their respective formations.

### Sensory deprivation has no effect on quantitative measures of network topology

The pial network can be conceptually subdivided into a backbone, formed by multiple connected LMAs, from which offshoots branches emerge and lead into penetrating arteries, with or without additional bifurcations along the offshoot branch. In the rodent these offshoots tend to be short, leading to PAs that dive immediately after leaving the pial backbone^99^. The offshoot’s topological structure can be quantitatively assessed by considering the number of vertices (either a bifurcation or a penetrating site) per branch (with longer branches with multiple bifurcation being a rare case). Here we report offshoot size by considering all vertices, regardless of location along the branch^99^.

We found that the offshoot branching structure in the forelimb/hindlimb region differed from the other cortical regions (see Statistical Supplement, p. 38). However, we found no effect of sensory deprivation on the overall offshoot branching structure (p = 0.17, χ^2^(1) = 1.86) or offshoot remodeling in any of the cortical regions (p = 0.78, χ^2^(4) = 1.75) (see Statistical Supplement, p. 36). Combined with the lack of LMA-associated structural changes, these results show that the topological structure of the network (both at the backbone and offshoot levels) was not affected by sensory deprivation.

## Discussion

**Figure 5:**
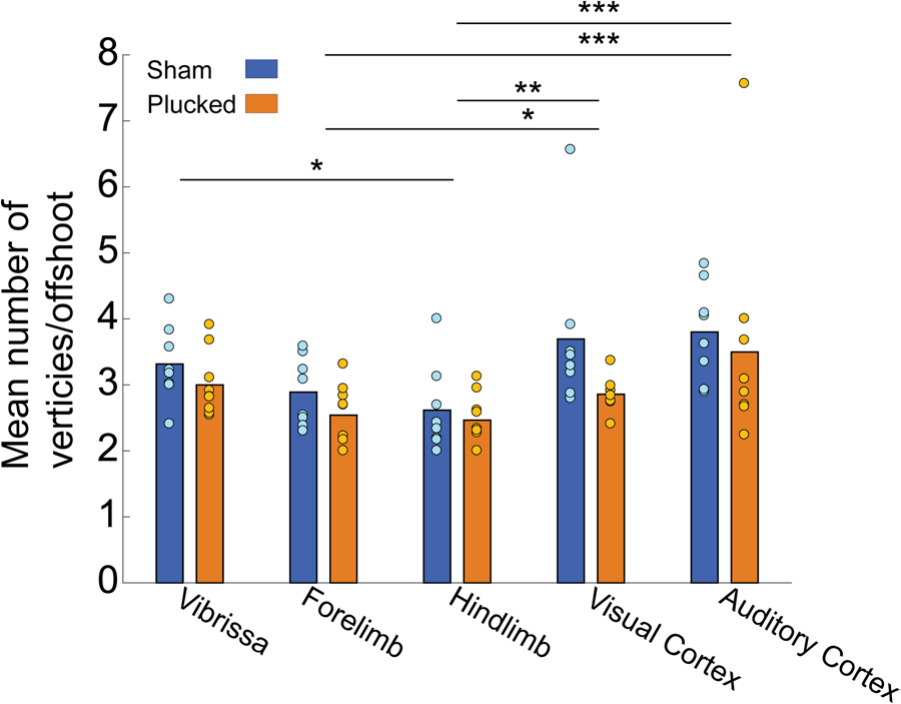
Quantification of network topology with a vertex-per-offshoot metric. Offshoot branches are vascular segments that emerge from the main backbone of the pial network (i.e. the portion that remains on the pial surface) and lead to penetrating arteries. For each such branch, we count the number of vertices (either a bifurcation or a penetrating artery). Plot of the mean vertex/offshoot ratio across cortical area. Bars show means, circles are individual animals. There was no effect of deprivation on the vertex/offshoot ratio, but the hindlimb area had a lower ratio than the vibrissa, visual and auditory cortex. The forelimb area had a significantly lower ratio than visual and auditory cortex. *p < 0.05; **p < 0.01; ***p < 0.001.

We analyzed the topology of the pial arteriole network to observe the effects of sensory deprivation on the number of penetrating arterioles, leptomeningeal anastomoses, and higher order network structure. Surprisingly, we found that the density of PAs was regularly distributed in primary sensory cortices despite the large variance in the number of PAs across animals (Fig. 2). While we did not analyze the capillary network density or structure, this result is consistent with previous studies that found penetrating arteriole distribution and capillary density to be unrelated to cortical columns^93,109^. We found a similar large variation in number of LMAs (Fig. 3) across animals that was unaffected by sensory deprivation. However, we observed a higher density of LMAs (Fig. 3) and a lower vertices-per-offshoot ratio in the hindlimb and forelimb representations than in visual and auditory areas (Fig. 5). These differences likely due to the position of the hindlimb region, right at the watershed between the MCA and PCA rather than some aspect of its neural activity, as hindlimb LMA density was higher than forelimb LMA density, and they both have similar patterns of vascular and neural activation during natural behaviors^54,60–62^, suggesting that these area-specific differences are not due to patterns of activation.

Why might pial arteries be less plastic than capillaries in response to alteration of neural activity? Previous work has found large differences in the architecture of the cerebral arterial network among mouse strains, showing a strong genetic component to the pial arterial network formation^42,88,108^. Recent single-cell transcriptomics work has elucidated the differential expression of genes by endothelial cell, smooth muscle and pericytes in blood vessels throughout the vascular tree^110^. It is likely that these vessel and cell-type specific gene expression contributes to differences in vascular remodeling. Recent mechanistic studies have also shown that pericytes play a role in maintaining capillary diameter^111^, potentially providing a mechanism for capillary plasticity. Lastly, arterioles have their own intrinsic vasomotor dynamics independent of neural activity^63,112^ which may play a more dominant role in shaping the pial arteriole network than neural activity.

This study aimed to elucidate the role of sensory deprivations on the preservation of vascular features during developmental pruning that provide fault tolerance, LMAs, as well as PAs within the arteriole network. Previous studies have shown that vibrissae deprivation lowers metabolic activity in the barrel cortex^31^, affected neural activity^22^, and capillary density^36^. Our results show that deprivation does not alter the structure of the pial arterial network. Because our studies were histological, we cannot rule out the possibilities sensory deprivation causes changes in blood flow or vessel tone. However, our results suggest that the large-scale vasculature structure is robust to perturbations of neural activity, and that there are region-specific differences in vascular structure that should be taken into account when comparing the hemodynamic responses in different areas. Specifically, the hindlimb representation has a substantially higher density of LMAs than other primary sensory regions. These area-specific differences could be used to study the roles of LMAs in vascular perfusion^43^ and neurovascular coupling^113^.

The LMAs of the pial vasculature play a critical role in rerouting blood around an occlusion of a pial arteriole^94–98^, reducing the size of the ischemic zone. Because of the LMAs imbue the pial network with a robustness against occlusions, any genetic or environmental factors that shape the development of the LMAs could impact stroke severity^43^. Previous work has found that large variations in the number of anastomoses (due to genetics, strain differences, age, or other factors), and these variations play a key role in determining the susceptibility to stroke^42,88,108,114–116^. Our work shows that, unlike genetic factors, early environmental experience, specifically sensory deprivation, does not have an impact on the connectivity of the pial vasculature.

## Methods

### Animals

All experimental procedures were approved by the Institutional Animal Care and Use Committee (IACUC) at the Pennsylvania State University. All experiments were performed in accordance with relevant guidelines and regulations. Both male (n = 9) and female (n = 10) mice from six different C57BL/6 litters were used in this study. All mice were obtained from Jackson Laboratory and bred in our vivarium. Mice were maintained on a 12-hour light-dark cycle with ad libitum access to food and water.

### Whisker deprivation

Plucking began on postnatal day 2 (P2). All pups in the litter (both sham and plucked) were simultaneously removed from their cage and placed in an incubator (Brinsea Products Inc, TLC-40 Advance Parrot Brooder, 35 °C and 25% humidity) before and after whisker manipulation. The pups were then rubbed with bedding from their cage before being returned to the dam to prevent rejection^20^. For the plucking/sham procedures, pups were initially induced with 5% isoflurane and oxygen as anesthesia, then maintained at 1–3% through the duration of the procedure. All macrovibrissae on the right whisker pad were plucked under a stereoscopic microscope. Care was taken to ensure the whisker follicles were not damaged during the procedure. Control mice were subjected to the same anesthetic regimen as plucked animals. The macrovibrissa on both sides of the control animals were gently stimulated during the procedure to mimic the manipulation required for whisker plucking. After the procedure, the mice were returned to the incubator and allowed to recover from the anesthesia, before being returned to the dam. Plucking was done every 24–36 hours until P30. We did not make within animal comparisons because whisker deprivation induces alterations of neural activity on the side ipsilateral to the whisker deprivation^91,92^.

### Histology

Between ages P45 and P65, mice were deeply anesthetized with 5% isoflurane in oxygen and pericardially perfused with physiological saline using a peristaltic pump. Once the perfusate was clear of blood, 25 mL of an India ink solution (1:2 ratio of India ink to physiological saline, filtered and heated to 65 °C)^117^ was infused using a luer-lock syringe. The ink perfusion filled arteries, capillaries and veins. Transcardial dye perfusion has the added advantage of only labeling perfused vascular vessels that will be supplying the brain with blood. The common transgenic labels for endothelial cells (e.g. Tie2) are also expressed by lymphatic vessels^118^. If any dura matter (which contains lymphatic vessels^119,120^) were to stay attached to the cortical surface, the use of genetic markers might falsely identify these lymphatic vessels as part of the cerebral vasculature. The mouse’s head was submerged in 4% paraformaldehyde in 0.1 mol/L phosphate buffer solution overnight. On the following day, the brain was extracted from the skull with great care to maintain pial vasculature, and sunk in 4% paraformaldehyde and 30% sucrose solution. In order to visualize the pial vascular network, the brain was dissected into cortical slabs for tangential sectioning and placed between two glass microscope slides^33,99^. High-resolution images were taken of the cortical slab pial vasculature before sectioning (Zeiss SteREO Discovery.V8 with Zeiss Achromat S 0.5 × FWD 134 mm objective; QImaging Retiga 2000R CCD camera). The cortical slabs were then mounted pial-side up on a freezing microtome and sectioned. The cortical sections were stained for cytochrome oxidase (CO)^121^ to visualize primary sensory areas (vibrissae-related cortex, the forelimb- and hindlimb-related regions, and both visual and auditory cortex). The boundaries between the cytochrome oxidase-delineated primary sensory areas and the lighter surrounding areas were manually traced. Given the contrast of the staining between the CO rich regions and the adjacent tissue, we estimate that we could reconstruct the boundaries of the cytochrome oxidase-rich sensory regions with ±50 µm accuracy. This is consistent with previous studies that used surface-recorded intrinsic optical signals to target neural recordings to a single barrel column^33,122^, and reported agreement between functional calcium signals and CO-delineated visual cortex^123^.

### Vascular and anatomical reconstructions

To create a complete image of the flattened cortical slab, vascular images were stitched together using Adobe Photoshop CS6 or the ImageJ plug-in MosaicJ. A manual tracing program (code available at: https://github.com/pbl007/dataExtractor_v2_1) was used to map the MCA and its connections to the anterior and posterior cerebral arteries (ACA and PCA, respectively) as well as penetrating arterioles (PAs), and LMAs, and codify the results in graph notation^99^. Mapping was done blinded to the treatment condition for 7 of the 19 mice. To determine the extent of the region fed by each PA, a Voronoi polygon tiling was generated, using the PAs as the centers of the polygons^94^. The surface vasculature reconstruction was aligned to the cytochrome oxidase labeled sensory areas using penetrating arterioles that were visible through the tangential sections. The watershed line was determined by manually bisecting the LMAs that connected the MCA territory to the ACA/PCA territories. These LMAs were identifiable as narrow vessels that joined two arterial bifurcations. In an anastomoses, the acute angles of these two bifurcations point towards each other^89,103^, rather than in the same direction, as is seen with offshoots off of an arteriole that is not an anastomoses^99^. LMAs often, but not always, exhibit a sinusoidal shape.

Using the cytochrome oxidase-stained sections as a reference, the spatial boundaries of the vibrissae representation, forelimb and hindlimb regions, and the primary visual and auditory cortices were aligned with the vascular graph in MATLAB. The area of each primary sensory region was calculated in MATLAB. No compensations for shrinkage due to fixation were performed. A PA was counted as being inside a sensory region only if the majority of the area enclosed by its Voronoi polygon was inside the region. While the topology of the network is preserved in the fixed brain, the dimeters of the fixed vessels will likely differ from the natural *in vivo* conditions. Because of this limitation, we did not quantify the pial vessel diameters.

### Statistics and Power calculations

We calculated (*post hoc*) that for our sample size, we could detect an effect size (d) of ~1.2, using a one-tailed t-test, a 1-beta of 0.8, and an alpha of 0.05 (G * Power)^124^. This effect size is much smaller than inter-strain differences in mice^88^, and should be more than adequate for detecting any physiologically relevant differences. Our sample size was comparable to, or larger than previous anatomical studies of the cerebral angioarchitecture^88,99,114,125^. In some cases where a complete reconstruction of a cortical region could not be performed, we omitted the cortical region from the relevant analysis.

### Generalized linear model

Counts of LMAs, PAs, and branching in multiple cortical areas from the same animal cannot be considered to be independent measurements. To account for the dependency between these data (which underestimates error variance), and to efficiently deal with the hierarchy of our experimental design, we used a generalized linear model (GLM)^90^ to test the effects of sensory deprivation on vascular structure. In addition, GLMs can account for non-normal error structures inherent to count data^126^, which are typically better approximated by Poisson or Negative Binomial distributions^127^.

We modeled the number of LMA and PA within a cortical region using a log-linear Poisson model as follows:

1 $$\mathrm{log}(\frac{C}{A})={\beta }_{0}+{\beta }_{1}R+\varepsilon $$

where $C$ was the LMA, PA, or branch count, $A$ was area (mm^2^) of the cortical region, R was a categorical variable denoting the identity of the cortical region, and ε was the measurement error. We tested whether the mean count (${\beta }_{0}$) was altered by sensory deprivation ($S$), age ($Y$), or sex ($G$). We also allowed for random variation ($u\,$_1_) in the overall PA/LMA counts due to individual mice ($M$):

2 $${\beta }_{0}={\gamma }_{1}S+{\gamma }_{2}Y+{\gamma }_{3}G+{u}_{1}M$$

We also allowed for the effect of age to differ among treatments ($S$):

3 $${\gamma }_{2}={\alpha }_{0}+{\alpha }_{1}S$$

and for sensory deprivation to differentially impact the region ($R$) specific counts:

4 $${\beta }_{1}={\gamma }_{2}+{\gamma }_{3}S$$

Thus, our full, mixed-effect model was:

5 $$\mathrm{log}(C)={\gamma }_{1}S+{\alpha }_{0}Y+{\alpha }_{1}(Y\ast S)+{\gamma }_{3}G+{\gamma }_{4}R+(S\ast R){\gamma }_{5}+M{u}_{1}+\,\mathrm{log}(A)$$

and $(\ast )$ denoted an interaction effect between treatment and region. Note that the term $\mathrm{log}(A)$ was an offset to the model to account for differences in areas of regions, not a free parameter to be fit. This model was fit using the lme4 package^128^ in R (version 3.4.2). Further details of the model are described in the Statistical Supplement (page 1).

To test whether a factor (specifically: sensory-deprivation, cortical region, age, sex) had a significant impact on overall LMA/PA/branch counts or LMA/PA/branch counts within a given area, we constructed a reduced model, which omitted the factor to be tested. We then conducted a maximum likelihood ratio (MLR) test between a model which included the factor and the reduced model. MLR tests yielding p-values <0.05 indicated that the factor contributed significantly to the variance of the count data. We performed post-hoc analysis on significant MLR results using Tukey’s honest significance difference (HSD) test to examine differences among the levels significant variables and correct for multiple comparisons.

To test whether the assumption of a Poisson error distribution was correct, we visually inspected the relationship between the model residuals and the fitted values, and saw no clear relationship between them. We conducted a χ^2^-test on the ratio of residual variance (v_r_) to residual mean (µ_r_) (H_a_: v_r_ > µ_r_, LMA: χ^2^(85) = 0.80, PA: χ^2^(85) = 0.98; H_a_: v_r_ < µ_r_, LMA: χ^2^(85) = 0.19, PA: χ^2^(85) = 0.03 (corrected)) which indicated that the mean-variance relationship was satisfied. Further details can be found in the Statistical Supplement (pages 19, 30, and 37).

### Data and code availability

Data and R code used in this paper is available for download at: https://github.com/DrewLab/Adams_Winder_Blinder_Drew_DataAndCode.

## Electronic supplementary material

Supplementary Material
