# Supplementary material for: The pial vasculature of the mouse develops according to a sensory-independent program
Source: Sci Rep. 2018 Jun 29;8:9860. doi: 10.1038/s41598-018-27910-3 (PMC6026131; doi:10.1038/s41598-018-27910-3)
Supplement: Supplementary file 1 — Supplementary Material [file 41598_2018_27910_MOESM1_ESM.pdf]

Title: The pial vasculature of the mouse develops according to a sensory-independent program

Authors: Matthew D. Adams, Aaron T. Winder, Pablo Blinder, Patrick J. Drew

The pial vasculature of the mouse develops according to a sensory-independent program

Explanation of the model

Overview:

This analysis tested the hypothesis that sensory deprivation (plucking whiskers) affected the distribution of leptomeningeal anastomoses (LMA), penetrating arterioles (PA) and the vessel branching structure. Mice received either the sensory-deprivation (plucked) or control (sham) treatment. The LMA/PA/branch count from 5 cortical regions (Auditory, Barrel, forelimb, hindlimb, Visual) was measured. The size (mm^2) of each cortical region was measured using cytochrome oxidase stains.

Approach: We chose to examine the effect of sensory deprivation on LMA/PA/branch counts using a generalized linear model for 2 reasons:

1. Inter-dependence of count data: Since counts were taken from multiple cortical regions in the same mouse, they cannot be considered to be independent of each other. This dependence of the data due to the nested structure violates the assumptions of basic tests such as ANOVA/t-tests, resulting in an underestimation of the residual variance and inflation of Type 1 error. Dependence of variance on the grouping of data is handled well by general linear models, which can include fixed and random effects.
2. Non-normality of errors: The LMA/PA count within each cortical region was not well represented by a normal distribution since counts are bounded by 0. Rather the data are likely to follow a Poisson or Negative Binomial distribution. A generalized linear model, which is an extension of the general linear model, can handle non-normal residuals.

The LMA/PA/branch counts can be conceptualized as a series of Bernoulli trials, where each trial evaluates the presence (success) or absence (failure) of an LMA/PA/branch within a given trial area. However, each cortical region was a different size and spanned a different number of "Bernoulli trial areas".

To account for the nested data structure, non-normal distribution, and varying size of cortical regions. We used a generalized linear model (GLM) based on a Poisson distribution with an offset term which models the logarithm of the count data as a linear combination of a set of independent variables:

(1)

$$log(C) = X\beta + Zu + \epsilon$$

where C was the LMA/PA count, X and Z were matrices of independent variables, β was a vector of the fixed-effects, u was a vector of the random effects, and ϵ was a vector of error.

At the lowest level of the nested data, the main factor influencing count data was assumed to be the cortical region (R):

(2)

$$log(C) = \beta_0 + R\beta_1 + \epsilon$$

where β_0 and β_1 denote columns of the matrix β. However, the count also scales with the size of the cortical area. This can be corrected by converting the count into a rate (count per unit area):

(3)

$$log(\frac{C}{A}) = \beta_0' + \beta_1' R + \epsilon$$

where A was a vector containing the area corresponding to each count in C.

Rearranging:

(4)
$$log(C) = \beta_0' + \beta_1' R + log(A) + \epsilon$$

The mean number of vessels (β_0') for a given animal may be affected by sensory-deprivation S (a fixed effect), Age (Y), Sex (G), and by the variation in counts due to differences between mice M (a random effect):

(5)
$$\beta_0' = \gamma_1 S + \gamma_2 Y + \gamma_3 G + u_1 M$$

where γ_1 was the fixed effects of sensory deprivation (S), γ_2 was the fixed-effect of age (Y), γ_3 was the fixed effect of sex (G), and u_1 was the random effect of an individual mice on the vessel mean. S and G were categorical variables, which was assigned a dummy variable (0 = sensory deprivation, 1 = sham; 0 = Female, 1 = Male). We also considered that the effect of age may be different among treatments:

(6)
$$\gamma_2 = \alpha_0 + \alpha_1 S$$

Additionally, since the whiskers were plucked we examined whether sensory deprivation impacted counts within cortical regions

(7)
$$\beta_1' = \gamma_4 + \gamma_5 S$$

where γ_4 was the fixed-effect of the cortical region, and γ_5 was the fixed-effect of the interaction (*) between sensory deprivation and cortical region. Combining equations (4) - (7), our final model was:

(8)
$$log(C) = \gamma_1 S + \alpha_0 Y + \alpha_1 (Y * S) + \gamma_3 G + \gamma_4 R + \gamma_5 (S * R) + u_1 M + log(A) + \epsilon$$

Instructions for replicating the results

The code blocks below contain commands in the “R” scripting language. To replicate the reported results the contents of the code blocks can be copied and pasted into the R-command line or the entire contents of the .rmd document can be loaded into R-Studio and run.

In order to run the code, the “tidyverse”, “lme4”, “multcomp”, and “knitr” packages must be installed into R.

Setup

Load Packages

```
library(tidyverse) # suite of packages for plotting and cleaning
```

```
## -- Attaching packages --------------------------------- tidyverse 1.2.1 --

## v ggplot2 2.2.1     v purrr   0.2.4
## v tibble  1.3.4     v dplyr   0.7.4
## v tidyr   0.7.2     v stringr 1.2.0
## v readr   1.1.1     v forcats 0.2.0

## -- Conflicts ------------------------------------ tidyverse_conflicts() --
## x dplyr::filter() masks stats::filter()
## x dplyr::lag()    masks stats::lag()
```

```
library(lme4) # for fitting the general linear model
```

```
## Loading required package: Matrix
```

```
##
## Attaching package: 'Matrix'
## The following object is masked from 'package:tidyr':
##
##     expand
```

```
library(multcomp) # for Tukey HSD test
```

```
## Loading required package: mvtnorm
## Loading required package: survival
## Loading required package: TH.data
## Loading required package: MASS
##
## Attaching package: 'MASS'
## The following object is masked from 'package:dplyr':
##
##     select
##
## Attaching package: 'TH.data'
## The following object is masked from 'package:MASS':
##
##     geyser
```

```
library(knitr) # for publication of markdown
```

Load Data

```
BranchData = read.table("../Data/OFFSHOOT_29Sep2017_004858.txt", header=T, sep="\t")
head(BranchData)
```

```
##      Name Group Area nBranches fractionOfBranches AvgNumVertInBranch
## 1 MDA101L  Sham  AUD        20        0.023148148           3.350000
## 2 MDA101L  Sham  VIS        64        0.074074074           3.500000
## 3 MDA101L  Sham HIND         7        0.008101852           2.428571
## 4 MDA101L  Sham FORE        13        0.015046296           3.230769
## 5 MDA101L  Sham   BF        53        0.061342593           3.018868
## 6 MDA105L  Sham  AUD        18        0.018887723           4.833333
##   AvgNumEdgesInBranch
## 1            2.350000
## 2            2.500000
## 3            1.428571
## 4            2.230769
## 5            2.018868
## 6            3.833333
```

```
PAData = read.table("../Data/LMA_PA_Data_Appended.txt", header = T, sep = "\t")
head(PAData)
```

```
##    Animal Treatment  Region NumLMA NumPA   Area Age Sex
## 1 MDA101L      Sham Barrels      3    31 2.7685  60   M
## 2 MDA105L      Sham Barrels      0    36 2.4597  62   F
```

```
## 3 MDA106L      Sham Barrels      2    48 3.3594  62   F
## 4 MDA307L      Sham Barrels      4    30 2.3019  60   M
## 5 MDA308L      Sham Barrels      1    36 2.4488  60   M
## 6 MDA401L      Sham Barrels      1    36 2.0627  50   F
```

Clean Data

```
# Recode the area variable
BranchClean <- BranchData %>%
  mutate(Area = recode(Area, AUD = "A", BF = "BF", FORE = "FL", HIND = "HL", VIS = "V"))

# Rename fieldnames of the data frames to match
colnames(BranchClean) <- c("Animal","Treatment","Region","nBranches","fractionOfBranches",
                           "AvgNumVertInBranch","AvgNumEdgesInBranch")

# PAData: Pipe1 - convert the NumLMA to numeric
#         Pipe2 - convert the NumPA to numeric
#         Pipe3 - recode the regions
PAClean <- PAData %>%
  mutate(NumLMA = as.numeric(levels(NumLMA)[NumLMA])) %>%
  mutate(NumPA = as.numeric(levels(NumPA)[NumPA])) %>%
  mutate(Region = recode(Region, Auditory = "A", Barrels = "BF", Forepaw = "FL",
                      Hindpaw = "HL", Visual = "V"))

# Merge the two data frames, Entries in PAData without a corresponding entry
# in BranchClean will be entered as NaN
# Pipe 1: Convert "Animal" variable to a factor
# Pipe 2: Create a new variable containing the density of LMA per mm^2
# Pipe 3: Create a new variable containing the density of PA per mm^2
Data <- as.tibble(left_join(PAClean,BranchClean) %>%
  mutate(Animal = factor(Animal)) %>%
  mutate(LMADensity = NumLMA/Area) %>%
  mutate(PADensity = NumPA/Area))
```

```
## Joining, by = c("Animal", "Treatment", "Region")
```

```
## Warning: Column `Animal` joining factors with different levels, coercing to
## character vector
```

```
# Pipe 1: Convert the "Animal" field from names to numbers
# Pipe 2: rescale the age variable
Data <- Data %>%
  mutate(Animal = factor(Data$Animal, level=levels(Data$Animal),
                         labels=c(1:nlevels(Data$Animal)))) %>%
  mutate(Age_scaled = scale(Age, center = T, scale = T))

# Clean data for figures
Data.Figs.RegionFilt <- dplyr::filter(Data, Region == "BF" | Region == "FL" | Region == "HL")
Data.Figs.Num <- mutate(aggregate(cbind(NumLMA, NumPA, Area) ~ Animal,
                                  data = Data.Figs.RegionFilt, sum, na.action = na.pass))
Data.Figs.Fctrs <- dplyr::filter(Data.Figs.RegionFilt, Region == "BF") %>%
  dplyr::select(Animal, Sex, Age, Age_scaled, Treatment) %>%
  dplyr::mutate(Age_scaled = Age_scaled[,1])
Data.Figs <- left_join(Data.Figs.Num, Data.Figs.Fctrs)
```

```
## Joining, by = "Animal"
```

```
Data.Figs.Complete = Data.Figs[complete.cases(Data.Figs),] %>%
  mutate(LMADensity = NumLMA/Area) %>%
  mutate(PADensity = NumPA/Area)
```

Compare the area of the cytochrome-oxidase stained regions between the sham and plucked conditions

Plot

```
ggplot(data = Data, mapping = aes(x = Treatment, y = Area)) +
  geom_point(na.rm = TRUE) +
  facet_wrap(~Region, nrow = 2) +
  labs(title = "Area of cortical regions compared between treatment conditions",
       x = "Treatment", y = "Sensory region area (mm^2)") +
  theme_bw()
```

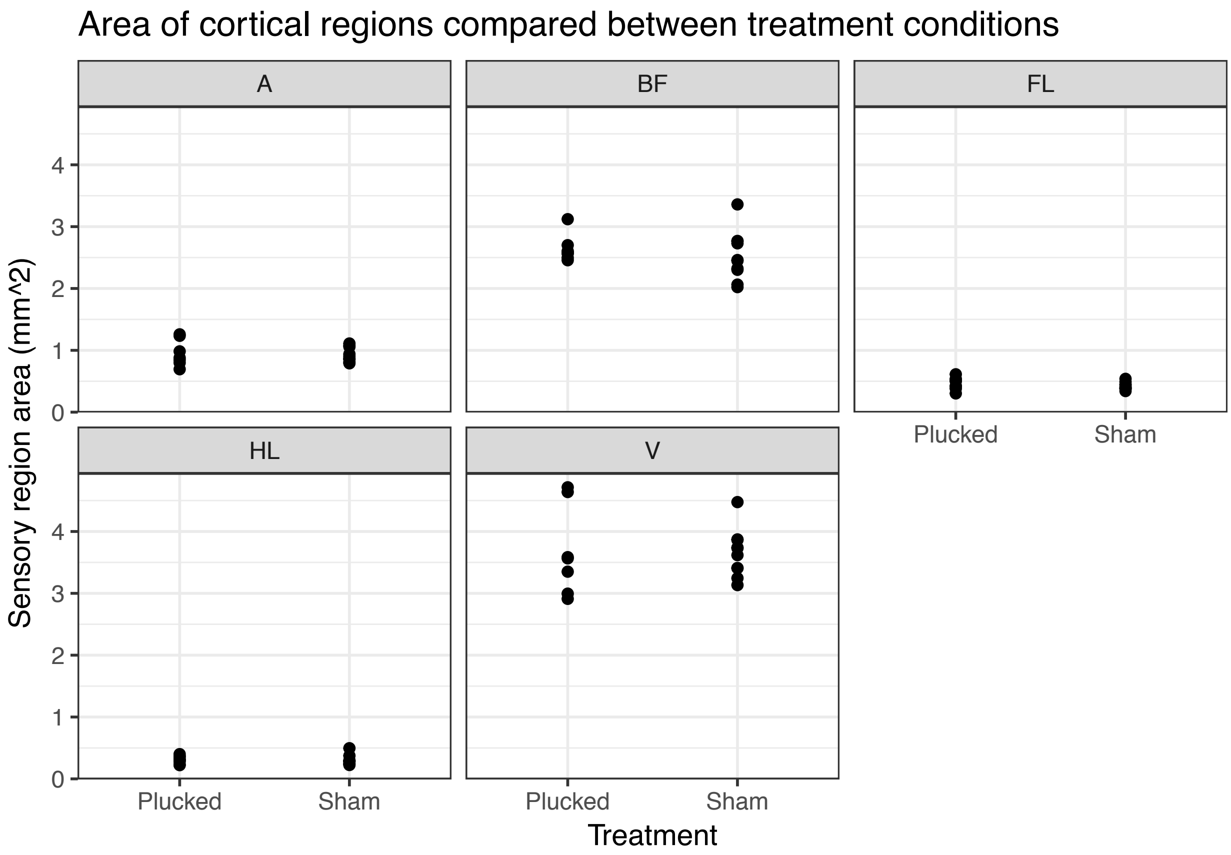

Test for differences in areas among treatments

```
AreaModel.Full = lm(data = Data, Area ~ Region*Treatment, na.action = na.omit)
summary(AreaModel.Full)
```

```
##
```

```
## Call:
## lm(formula = Area ~ Region * Treatment, data = Data, na.action = na.omit)
##
## Residuals:
##      Min       1Q   Median       3Q      Max
## -0.76909 -0.08298 -0.02710  0.06971  1.03451
##
## Coefficients:
##                           Estimate Std. Error t value Pr(>|t|)
## (Intercept)               0.956080   0.109814   8.706 3.03e-13 ***
## RegionBF                  1.675520   0.146419  11.443  < 2e-16 ***
## RegionFL                 -0.488260   0.146419  -3.335  0.00129 **
## RegionHL                 -0.647853   0.146419  -4.425 2.98e-05 ***
## RegionV                   2.723906   0.155301  17.540  < 2e-16 ***
## TreatmentSham            -0.027808   0.143181  -0.194  0.84649
## RegionBF:TreatmentSham -0.079692   0.195759  -0.407  0.68501
## RegionFL:TreatmentSham -0.019176   0.195759  -0.098  0.92221
## RegionHL:TreatmentSham  0.022088   0.195759   0.113  0.91044
## RegionV:TreatmentSham  -0.002738   0.202488  -0.014  0.98925
## ---
## Signif. codes:  0 '***' 0.001 '**' 0.01 '*' 0.05 '.' 0.1 ' ' 1
##
## Residual standard error: 0.2905 on 81 degrees of freedom
##   (4 observations deleted due to missingness)
## Multiple R-squared:  0.9577, Adjusted R-squared:  0.953
## F-statistic: 203.9 on 9 and 81 DF,  p-value: < 2.2e-16
```

Comment: The linear model indicates that the only factor affecting the size of the cytochrome stained area is the identity of the cortical region. There was not an effect of treatment on the area overall, or for any individual region ($p > 0.05$).

Plot the number of LMAs vs. PAs

```
# Fit the points
LMA.Model.PA.All = lm(data = Data.Figs.Complete,
                           LMADensity ~ PADensity, na.action = na.omit)
Data.Figs.Plucked <- dplyr::filter(Data.Figs.Complete, Treatment == "Plucked")
LMA.Model.PA.Plucked = lm(data = Data.Figs.Plucked,
                           LMADensity ~ PADensity, na.action = na.omit)
Data.Figs.Sham <- dplyr::filter(Data.Figs.Complete, Treatment == "Sham")
LMA.Model.PA.Sham = lm(data = Data.Figs.Sham,
                        LMADensity ~ PADensity, na.action = na.omit)

# Summarize results as a table
LMA.Table.PA <- tibble(
  Treatment = c("All","Plucked","Sham"),
  Slope = c(summary(LMA.Model.PA.All)$coefficients[2,1],
            summary(LMA.Model.PA.Plucked)$coefficients[2,1],
            summary(LMA.Model.PA.Sham)$coefficients[2,1]),
  tStat = c(summary(LMA.Model.PA.All)$coefficients[2,3],
            summary(LMA.Model.PA.Plucked)$coefficients[2,3],
            summary(LMA.Model.PA.Sham)$coefficients[2,3]),
  pVal = c(summary(LMA.Model.PA.All)$coefficients[2,4],
```

```
            summary(LMA.Model.PA.Plucked)$coefficients[2,4],
            summary(LMA.Model.PA.Sham)$coefficients[2,4])
  )
kable(LMA.Table.PA)
```

Treatment	Slope	tStat	pVal
All	0.0673640	1.5422802	0.1414143
Plucked	0.2071705	3.0988325	0.0173507
Sham	0.0103519	0.2776666	0.7883089

```
# Plot
ggplot(data = Data.Figs.Complete, mapping = aes(x = PADensity, y = LMADensity, color = Treatment)) +
  geom_point() +
  scale_color_manual(values = c("orange","blue")) +
  geom_line(mapping = aes(x = PADensity, y = fitted(LMA.Model.PA.All)),
          color = "black", size = 1) +
  geom_line(Data.Figs.Plucked, mapping = aes(x = PADensity, y = fitted(LMA.Model.PA.Plucked)),
          color = "orange", size = 1) +
  geom_line(Data.Figs.Sham, mapping = aes(x = PADensity, y = fitted(LMA.Model.PA.Sham)),
          color = "blue", size = 1) +
  labs(x = "PA Density (PA/mm^2)", y = "LMA Density (LMA/mm^2)",
       title = "Density of PAs and LMAs in the BF, FL, HL") +
  xlim(10,30) +
  ylim(0,3) +
  theme_bw()
```

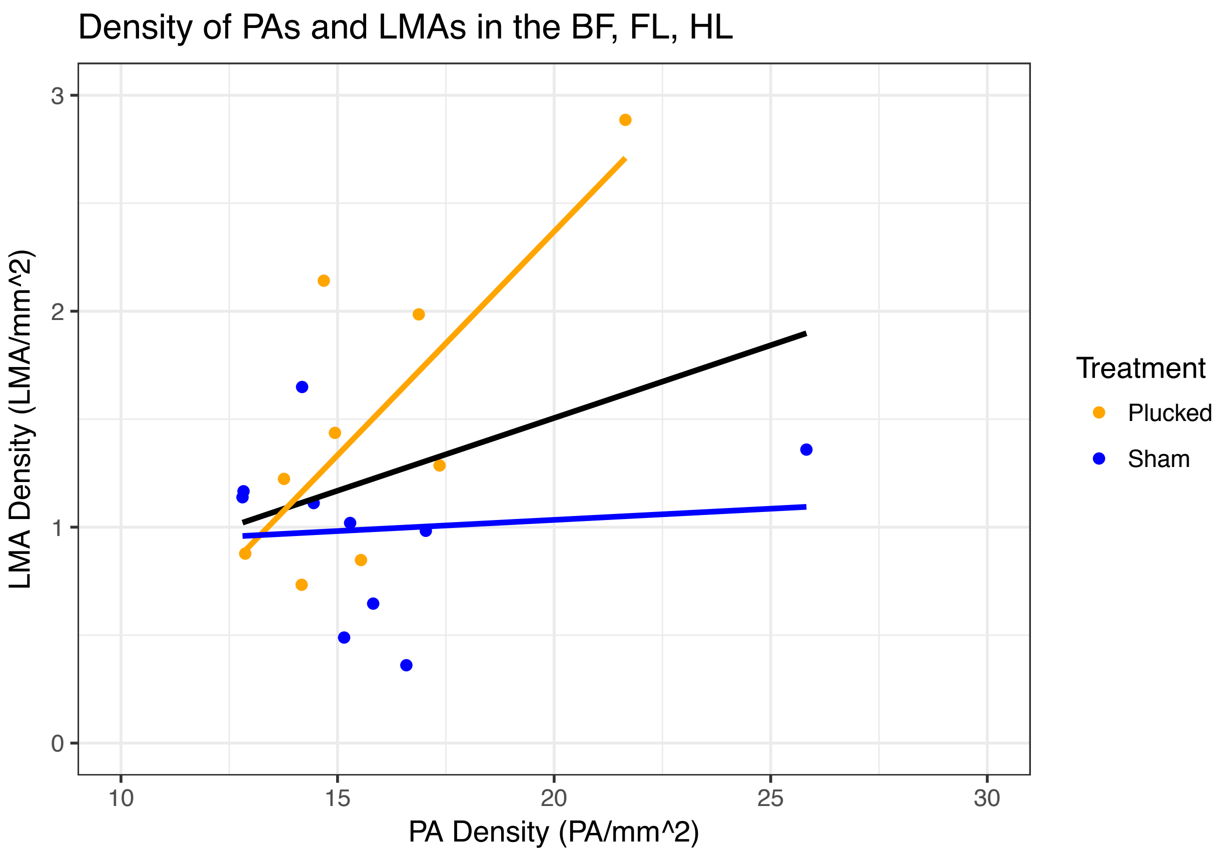


```r
ggsave("NumPAvsNumLMA.eps")
```

```
## Saving 6.5 x 4.5 in image
```

Effect of sensory deprivation on LMA count

Hypothesis: Sensory deprivation (whisker plucking) alters the number LMA within individual cortical regions

Null hypothesis (H_0): Sensory deprivation DOES NOT affect number of LMAs in a cortical region.

Alternative hypothesis (H_a): Sensory deprivation DOES affect the number of LMAs in a cortical region.

Plot the distribution of LMA counts

```r
ggplot(data = Data, mapping = aes(x=NumLMA)) +
  geom_histogram(bins = 15, na.rm = TRUE) +
  theme_bw() +
  xlim(0,15) +
  labs(title = "Distribution of all LMA counts:", x = "LMA count", y = "Frequency")
```

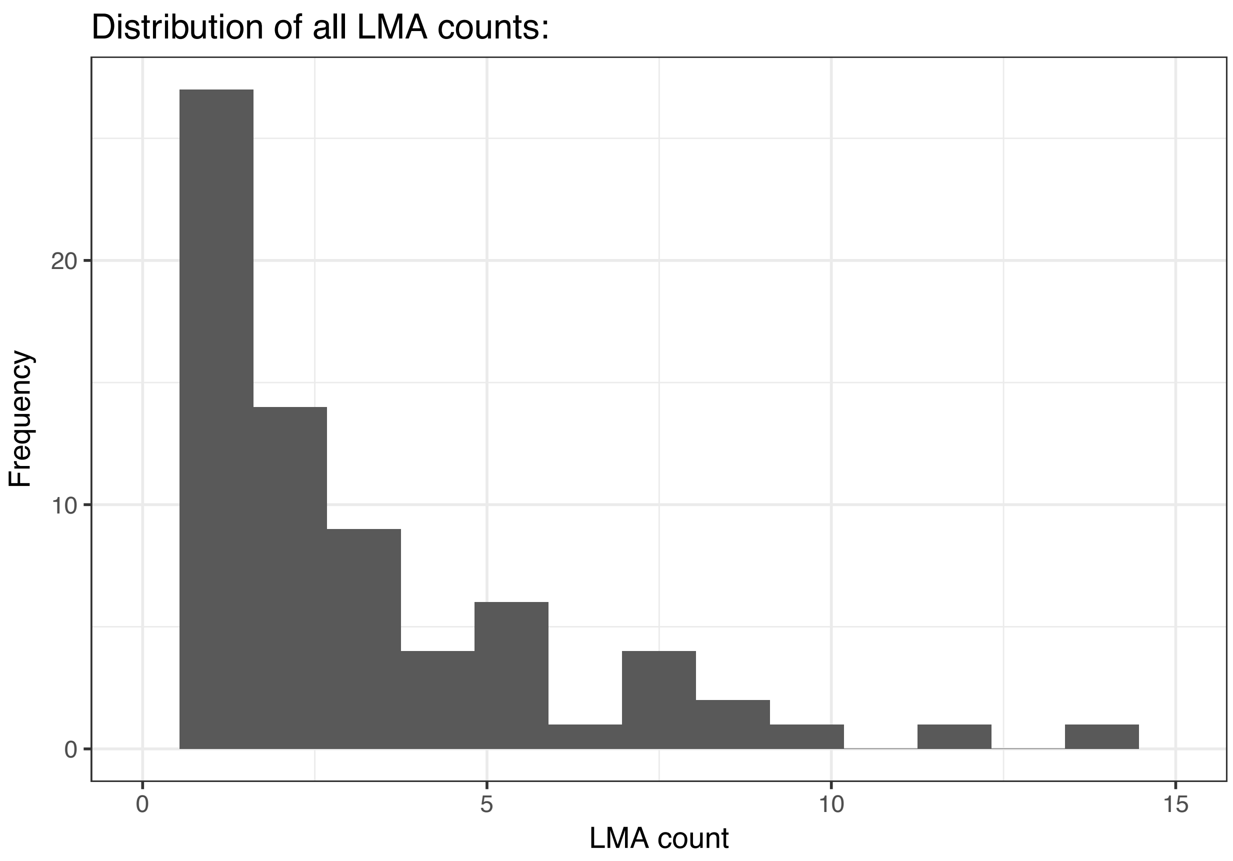

Plot the LMA *counts* by Region, Treatment, and Animal

```
ggplot(data = Data, mapping = aes(x=Region, y=NumLMA, group = Animal, shape = Sex)) +
  geom_point(na.rm = TRUE, mapping = aes(col = Animal), size = 4) +
  geom_line(na.rm = TRUE, mapping = aes(col = Animal), size = 1) +
  facet_wrap(~Treatment, nrow = 1) +
  labs(title = "LMA counts for each animal:", x = "Cortical region", y = "LMA count") +
  guides(color = FALSE) +
  theme_bw()
```

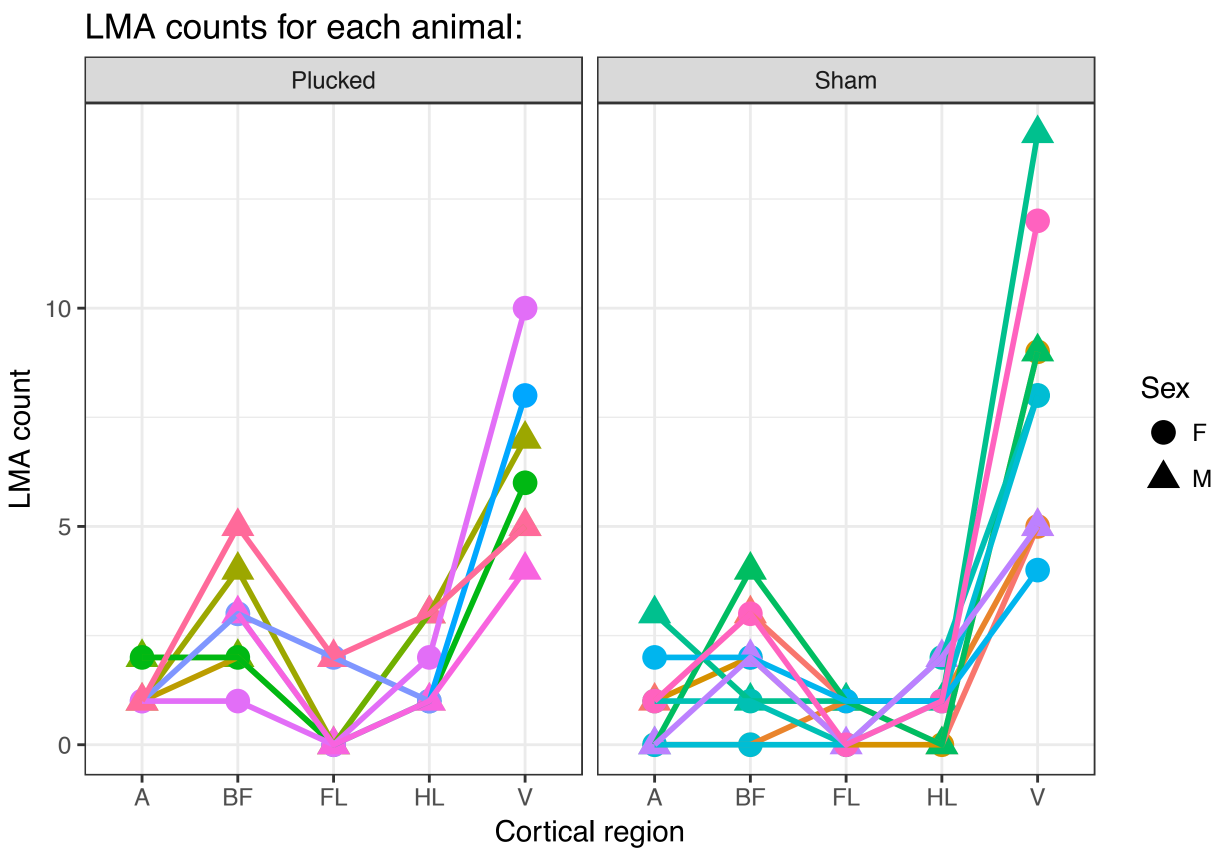

Plot the LMA *density* by Region, Treatment, and Animal

```
ggplot(data = Data, mapping = aes(x=Region, y=LMADensity, group = Animal, shape = Sex)) +
  geom_point(na.rm = TRUE, mapping = aes(col = Animal), size = 4) +
  geom_line(na.rm = TRUE, mapping = aes(col = Animal), size = 1) +
  facet_wrap(~Treatment, nrow = 1) +
  labs(title = "LMA density for each animal:", x = "Cortical region",
       y = "LMA density (LMA*mm-2)") +
  guides(color = FALSE) +
  theme_bw()
```

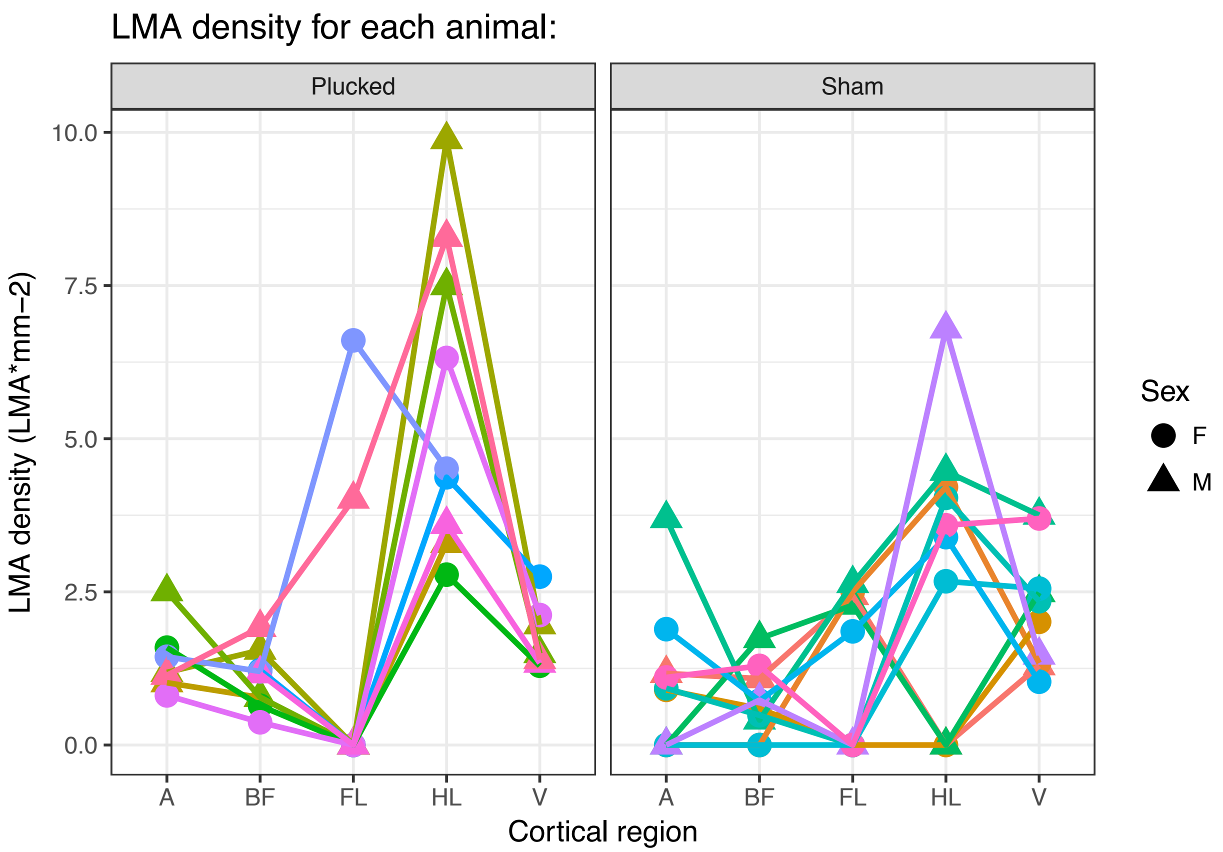

Plot the relationship between Age and total LMA count

```
# Build models
LMA.Model.Age.All = lm(data = Data.Figs.Complete,
                           LMADensity ~ Age_scaled, na.action = na.omit)
Data.Figs.Plucked <- dplyr::filter(Data.Figs.Complete, Treatment == "Plucked")
LMA.Model.Age.Plucked = lm(data = Data.Figs.Plucked,
                           LMADensity ~ Age_scaled, na.action = na.omit)
Data.Figs.Sham <- dplyr::filter(Data.Figs.Complete, Treatment == "Sham")
LMA.Model.Age.Sham = lm(data = Data.Figs.Sham,
                        LMADensity ~ Age_scaled, na.action = na.omit)

# Summarize model results in table
LMA.Table.Age <- tibble(
  Treatment = c("All","Plucked","Sham"),
  Slope = c(summary(LMA.Model.Age.All)$coefficients[2,1],
            summary(LMA.Model.Age.Plucked)$coefficients[2,1],
            summary(LMA.Model.Age.Sham)$coefficients[2,1]),
  tStat = c(summary(LMA.Model.Age.All)$coefficients[2,3],
            summary(LMA.Model.Age.Plucked)$coefficients[2,3],
            summary(LMA.Model.Age.Sham)$coefficients[2,3]),
  pVal = c(summary(LMA.Model.Age.All)$coefficients[2,4],
           summary(LMA.Model.Age.Plucked)$coefficients[2,4],
           summary(LMA.Model.Age.Sham)$coefficients[2,4])
```

```
  )
kable(LMA.Table.Age)
```

Treatment	Slope	tStat	pVal
All	-0.1681540	-1.2125910	0.2418689
Plucked	-0.2284799	-0.9068762	0.3946176
Sham	-0.0565696	-0.4346794	0.6752821

```
# Plot
ggplot(data = Data.Figs.Complete, mapping = aes(x = Age, y = LMADensity)) +
  geom_point(mapping = aes(color = Treatment), size = 3) +
  scale_color_manual(values = c("orange","blue")) +
  geom_line(mapping = aes(x = Age, y = fitted(LMA.Model.Age.All)),
            color = "black", size = 1) +
  geom_line(Data.Figs.Plucked, mapping = aes(x = Age, y = fitted(LMA.Model.Age.Plucked)),
            color = "orange", size = 1) +
  geom_line(Data.Figs.Sham, mapping = aes(x = Age, y = fitted(LMA.Model.Age.Sham)),
            color = "blue", size = 1) +
  labs(x = "Age (days)", y = "LMA density", title = "Effect of age on Number of LMAs:") +
  theme_bw()
```

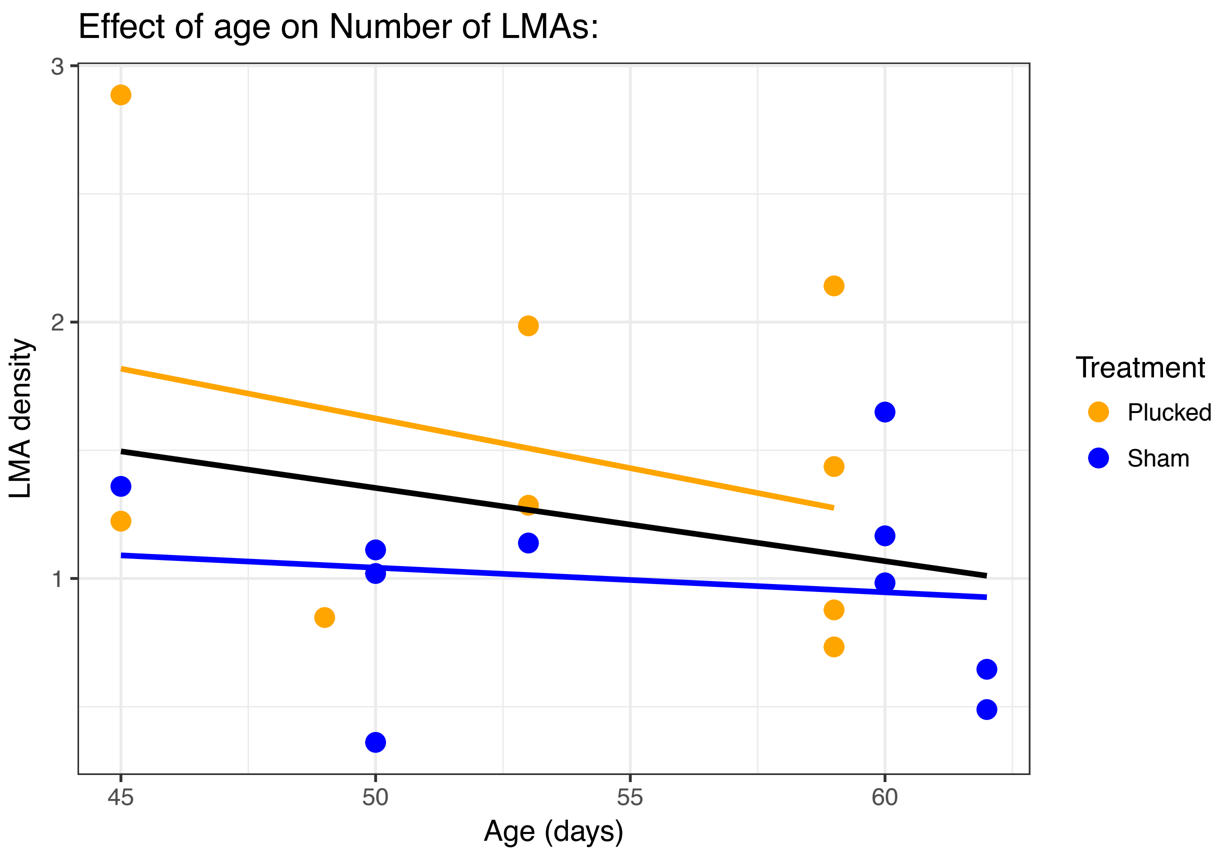


```
ggsave("EffectOfAgeOnNumberOfLMAs.eps")

## Saving 6.5 x 4.5 in image
```

Model the LMA count

```
LMA.Poisson.Full = glmer(NumLMA ~ 1 + Age_scaled + Sex + Treatment + Region  +
                           Treatment:Region + Age_scaled:Treatment + (1|Animal) +
                           offset(log(Area)),
                         data = Data, family = poisson,
                         control = glmerControl(optimizer="bobyqa"), na.action = na.omit)
LMA.Summary.Poisson.Full = summary(LMA.Poisson.Full)
LMA.Summary.Poisson.Full
```

```
## Generalized linear mixed model fit by maximum likelihood (Laplace
##   Approximation) [glmerMod]
##  Family: poisson  ( log )
## Formula:
## NumLMA ~ 1 + Age_scaled + Sex + Treatment + Region + Treatment:Region +
##     Age_scaled:Treatment + (1 | Animal) + offset(log(Area))
##    Data: Data
## Control: glmerControl(optimizer = "bobyqa")
##
##      AIC      BIC   logLik deviance df.resid
##    291.6    326.7   -131.8    263.6       77
##
## Scaled residuals:
##     Min      1Q  Median      3Q     Max
## -1.5574 -0.6740 -0.1173  0.4263  3.3976
##
## Random effects:
##  Groups Name        Variance Std.Dev.
##  Animal (Intercept) 0        0
## Number of obs: 91, groups:  Animal, 19
##
## Fixed effects:
##                           Estimate Std. Error z value Pr(>|z|)
## (Intercept)                0.20510    0.34325   0.598 0.550162
## Age_scaled                -0.05300    0.09964  -0.532 0.594778
## SexM                       0.17151    0.14530   1.180 0.237853
## TreatmentSham             -0.29327    0.47221  -0.621 0.534556
## RegionBF                  -0.25708    0.38933  -0.660 0.509053
## RegionFL                  -0.36318    0.60124  -0.604 0.545808
## RegionHL                   1.43391    0.41724   3.437 0.000589 ***
## RegionV                    0.24408    0.36666   0.666 0.505611
## TreatmentSham:RegionBF    -0.04698    0.56420  -0.083 0.933636
## TreatmentSham:RegionFL     0.56419    0.82013   0.688 0.491492
## TreatmentSham:RegionHL    -0.31889    0.62958  -0.507 0.612492
## TreatmentSham:RegionV      0.56341    0.50824   1.109 0.267626
## Age_scaled:TreatmentSham -0.04604    0.14027  -0.328 0.742750
## ---
## Signif. codes:  0 '***' 0.001 '**' 0.01 '*' 0.05 '.' 0.1 ' ' 1

##
## Correlation matrix not shown by default, as p = 13 > 12.
## Use print(x, correlation=TRUE)  or
##   vcov(x)     if you need it
```

Comment: The fixed effects are compared to the intercept which is the log(mean) of the LMA count in

auditory cortex for sensory deprived mice. The model summary suggested that cortical region is likely the only significant effect. We used maximum likelihood ratios to test the effects directly.

Perform likelihood ratio tests to test main and interaction effects:

Test the significance of Sex on the LMA counts

```
LMA.Poisson.Reduced1 = glmer(NumLMA ~ 1 + Age_scaled + Treatment + Region  +
                               Treatment:Region + Age_scaled:Treatment + (1|Animal) +
                               offset(log(Area)),
                        data = Data, family = poisson,
                        control = glmerControl(optimizer="bobyqa"), na.action = na.omit)
anova(LMA.Poisson.Full, LMA.Poisson.Reduced1)
```

```
## Data: Data
## Models:
## LMA.Poisson.Reduced1: NumLMA ~ 1 + Age_scaled + Treatment + Region + Treatment:Region +
## LMA.Poisson.Reduced1:     Age_scaled:Treatment + (1 | Animal) + offset(log(Area))
## LMA.Poisson.Full: NumLMA ~ 1 + Age_scaled + Sex + Treatment + Region + Treatment:Region +
## LMA.Poisson.Full:     Age_scaled:Treatment + (1 | Animal) + offset(log(Area))
##                      Df    AIC    BIC  logLik deviance  Chisq Chi Df
## LMA.Poisson.Reduced1 13 290.96 323.60 -132.48   264.96
## LMA.Poisson.Full     14 291.56 326.71 -131.78   263.56 1.3998      1
##                      Pr(>Chisq)
## LMA.Poisson.Reduced1
## LMA.Poisson.Full         0.2368
```

Comment: the p-value (p=0.2368) indicated that the model was not significantly improved by inclusion of sex as a factor and there was no effect of sex on mean LMA counts.

Test the significance of Age on LMA counts

```
LMA.Poisson.Reduced2 = glmer(NumLMA ~ 1 + Treatment + Region  + Treatment:Region +
                               (1|Animal) + offset(log(Area)),
                        data = Data, family = poisson,
                        control = glmerControl(optimizer="bobyqa"), na.action = na.omit)
anova(LMA.Poisson.Reduced1, LMA.Poisson.Reduced2)
```

```
## Data: Data
## Models:
## LMA.Poisson.Reduced2: NumLMA ~ 1 + Treatment + Region + Treatment:Region + (1 | Animal) +
## LMA.Poisson.Reduced2:     offset(log(Area))
## LMA.Poisson.Reduced1: NumLMA ~ 1 + Age_scaled + Treatment + Region + Treatment:Region +
## LMA.Poisson.Reduced1:     Age_scaled:Treatment + (1 | Animal) + offset(log(Area))
##                      Df    AIC   BIC  logLik deviance  Chisq Chi Df
## LMA.Poisson.Reduced2 11 287.78 315.4 -132.89   265.78
## LMA.Poisson.Reduced1 13 290.96 323.6 -132.48   264.96 0.8259      2
##                      Pr(>Chisq)
## LMA.Poisson.Reduced2
## LMA.Poisson.Reduced1     0.6617
```

Comment: the p-value (p=0.6617) indicated that the model was not significantly improved by including age as a factor and there was no effect of age on mean LMA counts for all animals or for either treatment group.

Test the significance of the effect of sensory deprivation on LMA counts within cortical regions

```
LMA.Poisson.Reduced3 = glmer(NumLMA ~ 1 + Treatment + Region + (1|Animal) +
                                 offset(log(Area)), data = Data,
                                 family = poisson,
                                 control = glmerControl(optimizer="bobyqa"),
                                 na.action = na.omit)
anova(LMA.Poisson.Reduced2, LMA.Poisson.Reduced3, test = "Chisq")
```

```
## Data: Data
## Models:
## LMA.Poisson.Reduced3: NumLMA ~ 1 + Treatment + Region + (1 | Animal) + offset(log(Area))
## LMA.Poisson.Reduced2: NumLMA ~ 1 + Treatment + Region + Treatment:Region + (1 | Animal) +
## LMA.Poisson.Reduced2:     offset(log(Area))
##                      Df    AIC   BIC  logLik deviance  Chisq Chi Df
## LMA.Poisson.Reduced3  7 285.82 303.4 -135.91   271.82
## LMA.Poisson.Reduced2 11 287.78 315.4 -132.89   265.78 6.0428      4
##                      Pr(>Chisq)
## LMA.Poisson.Reduced3
## LMA.Poisson.Reduced2      0.196
```

Comment: the p-value (p=0.196) indicated that the model was not significantly improved by including the interaction between Treatment and Region and that there was no differential, region-specific effect of the sensory deprivation.

Test the significance of the effect of sensory deprivation on LMA count

```
LMA.Poisson.Reduced4 = glmer(NumLMA ~ 1 + Region + (1|Animal) + offset(log(Area)),
                               data = Data, family = poisson,
                               control = glmerControl(optimizer="bobyqa"),
                               na.action = na.omit)
LMA.Summary.Poisson.Reduced4 = summary(LMA.Poisson.Reduced4)
anova(LMA.Poisson.Reduced3, LMA.Poisson.Reduced4, test = "Chisq")
```

```
## Data: Data
## Models:
## LMA.Poisson.Reduced4: NumLMA ~ 1 + Region + (1 | Animal) + offset(log(Area))
## LMA.Poisson.Reduced3: NumLMA ~ 1 + Treatment + Region + (1 | Animal) + offset(log(Area))
##                      Df    AIC    BIC  logLik deviance  Chisq Chi Df
## LMA.Poisson.Reduced4  6 283.98 299.04 -135.99   271.98
## LMA.Poisson.Reduced3  7 285.82 303.40 -135.91   271.82 0.1512      1
##                      Pr(>Chisq)
## LMA.Poisson.Reduced4
## LMA.Poisson.Reduced3     0.6974
```

Comment: the p-value (p=0.697) indicated that the model was not significantly improved by including the overall effect of Treatment on the LMA count and that the sensory deprivation did not alter the number of LMAs.

Test the significance of the effect of cortical region on LMA count

```r
LMA.Poisson.Reduced5 = glmer(NumLMA ~ 1 + (1|Animal) + offset(log(Area)),
                             data = Data, family = poisson,
                             control = glmerControl(optimizer="bobyqa"),
                             na.action = na.omit)
anova(LMA.Poisson.Reduced4, LMA.Poisson.Reduced5, test = "Chisq")
```

```
## Data: Data
## Models:
## LMA.Poisson.Reduced5: NumLMA ~ 1 + (1 | Animal) + offset(log(Area))
## LMA.Poisson.Reduced4: NumLMA ~ 1 + Region + (1 | Animal) + offset(log(Area))
##                      Df    AIC    BIC  logLik deviance Chisq Chi Df
## LMA.Poisson.Reduced5  2 322.60 327.62 -159.30   318.60
## LMA.Poisson.Reduced4  6 283.98 299.04 -135.99   271.98 46.62      4
##                      Pr(>Chisq)
## LMA.Poisson.Reduced5
## LMA.Poisson.Reduced4   1.83e-09 ***
## ---
## Signif. codes:  0 '***' 0.001 '**' 0.01 '*' 0.05 '.' 0.1 ' ' 1
```

Comment: the p-value (p=1.83e-09) indicated that the model was signficantly improved by including the effect of cortical region on the LMA count and that LMA counts were different between regions.

Use Tukey's honest significance difference-test to test which regions are different.

Tukey's HSD evaluated signficance based on a studentized distribution that corrects for multiple comparisons.

```r
LMA.PostHoc.Region = glht(LMA.Poisson.Reduced4, mcp(Region = "Tukey"))
LMA.Summary.PostHoc.Region = summary(LMA.PostHoc.Region)
(LMA.Summary.PostHoc.Region)
```

```
##
##   Simultaneous Tests for General Linear Hypotheses
##
## Multiple Comparisons of Means: Tukey Contrasts
##
##
## Fit: glmer(formula = NumLMA ~ 1 + Region + (1 | Animal) + offset(log(Area)),
##     data = Data, family = poisson, control = glmerControl(optimizer = "bobyqa"),
##     na.action = na.omit)
##
## Linear Hypotheses:
##              Estimate Std. Error z value Pr(>|z|)
## BF - A == 0  -0.24902    0.28080  -0.887  0.89371
## FL - A == 0  -0.05256    0.40826  -0.129  0.99993
## HL - A == 0   1.34125    0.30917   4.338  < 0.001 ***
## V - A == 0    0.56957    0.25231   2.257  0.14678
## FL - BF == 0  0.19646    0.36657   0.536  0.98200
## HL - BF == 0  1.59026    0.25153   6.322  < 0.001 ***
## V - BF == 0   0.81859    0.17707   4.623  < 0.001 ***
## HL - FL == 0  1.39381    0.38874   3.585  0.00279 **
## V - FL == 0   0.62213    0.34525   1.802  0.35200
## V - HL == 0  -0.77168    0.21932  -3.518  0.00361 **
## ---
## Signif. codes:  0 '***' 0.001 '**' 0.01 '*' 0.05 '.' 0.1 ' ' 1
```

```
## (Adjusted p values reported -- single-step method)
```

```
# Extract the estimates for the significant results
LMA.SigCoef.PostHoc.Region <-
  LMA.Summary.PostHoc.Region$test$coefficients[LMA.Summary.PostHoc.Region$test$pvalues < 0.05]

# Extract the effect size for the signficant results
LMA.SigZ.PostHoc.Region <-
  LMA.Summary.PostHoc.Region$test$tstat[LMA.Summary.PostHoc.Region$test$pvalues < 0.05]

#Get the significant p-values
LMA.SigP.PostHoc.Region <-
  LMA.Summary.PostHoc.Region$test$pvalues[LMA.Summary.PostHoc.Region$test$pvalues < 0.05]

plot(LMA.PostHoc.Region, xlab = "Difference in log(mean) of LMA")
```

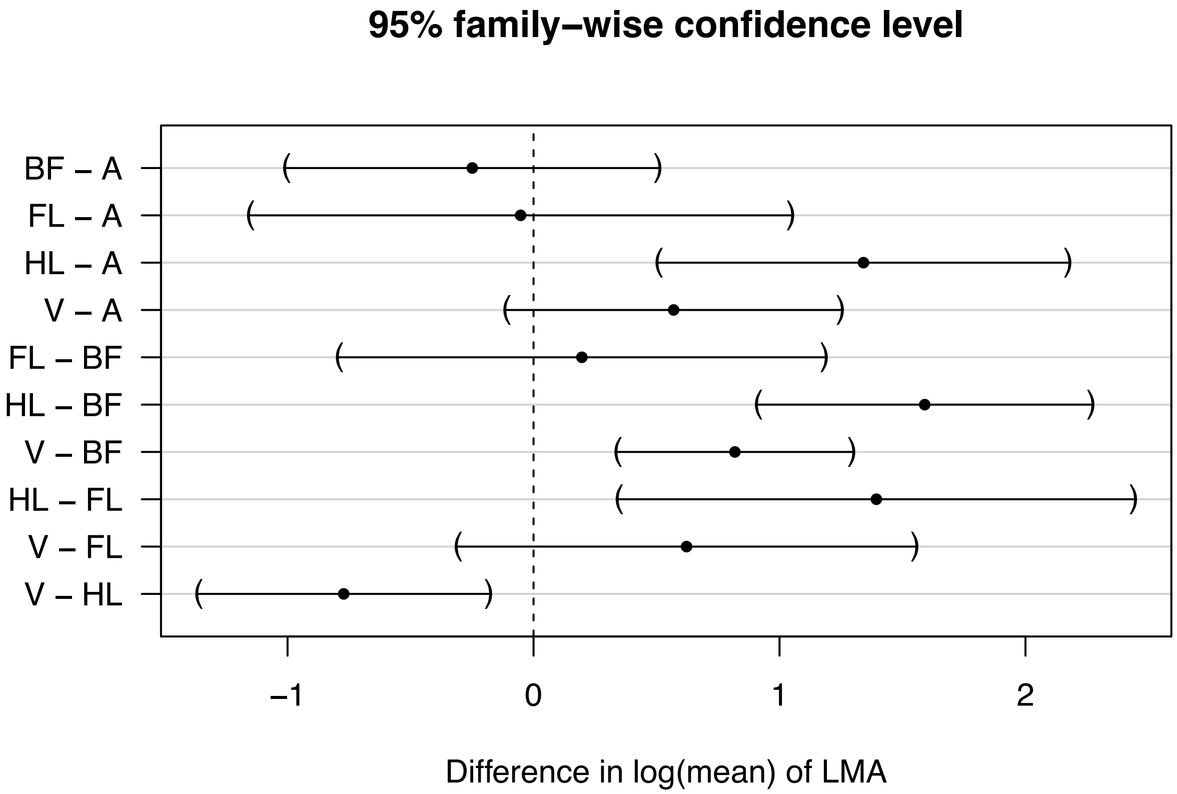

Comment: The Tukey-adjusted p-values indicated that the hindlimb, barrel, and visual cortices varied in their LMA counts.

Test model assumptions

Test assumption of Poisson distribution

An assumption of the Poisson distribution was that the mean and variance of the error distribution was equal. The error was distributed according to χ^2, where, by definition, the mean = degrees of freedom. Thus, the Poisson assumption can be tested by comparing the residual degrees of freedom (= mean residual) to the variance of the (studentized) residual (rp):

(9)

$$rp = \frac{actual - fitted}{\sqrt{fitted}}$$

```r
# Get the degrees of freedom for the residuals (rdf)
rdf <- LMA.Summary.Poisson.Reduced4$AICtab[[5]]

# Get the studentized residuals of the model
rp <- residuals(LMA.Poisson.Reduced4,type="pearson")

# Calculate the variance of the studentized residuals
rp_var <- sum(rp^2)

# Show the ratio of error variance, mean
rat = rp_var/rdf

# Get the probability (pval) that the variance > mean
pval_over <- pchisq(rp_var, df = rdf, lower.tail = FALSE)
pval_under <- pchisq(rp_var, df = rdf, lower.tail = TRUE)

# Summarize
overdisp = c(Residual_Variance = rp_var, Ratio = rat, df = rdf,
             p_over = pval_over, p_under = pval_under)
overdisp
```

```
## Residual_Variance             Ratio                df            p_over
##        73.5444383         0.8652287        85.0000000         0.8077790
##           p_under
##         0.1922210
```

Plot the fitted values against the residuals

```r
# Calculate the studentized residuals
Resids = data.frame(Mouse_Number = Data[complete.cases(Data$NumLMA),]$Animal,
                    Fitted_Value = fitted(LMA.Poisson.Reduced4),
                    Standardized_Resids = residuals(LMA.Poisson.Reduced4, type="pearson"))

# Fit the plot with a line to test whether slope = 0
Resids.Fitted = lm(Standardized_Resids~Fitted_Value, data = Resids)

# Plot the studentized residual amplitude vs. the fitted value
plot(LMA.Poisson.Reduced4)
```

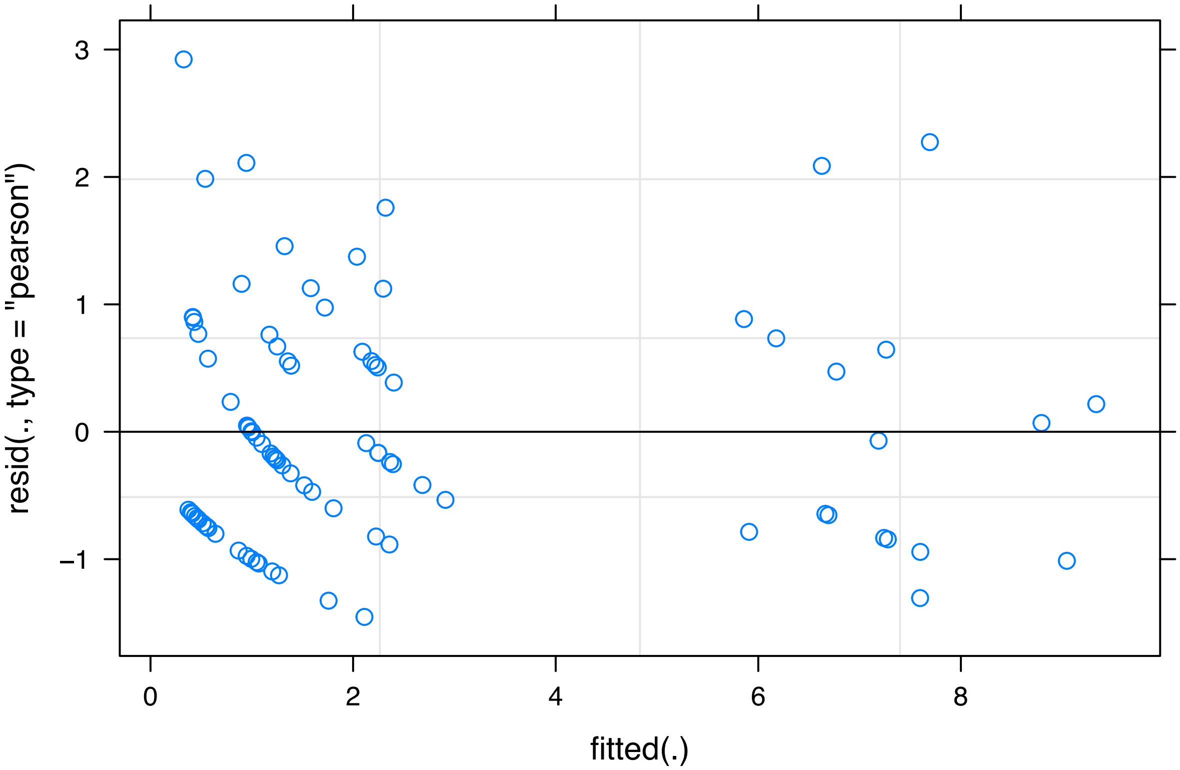


```r
# Test the significance of the slope
anova(Resids.Fitted)
```

```
## Analysis of Variance Table
##
## Response: Standardized_Resids
##               Df Sum Sq Mean Sq F value Pr(>F)
## Fitted_Value  1  0.006 0.00571  0.0069 0.9339
## Residuals    89 73.534 0.82623
```

Interpretation: The ratio $\frac{Var_{error}}{mean_{error}} = \frac{73.54}{85} = 0.865$ was not significantly greater than 1 (p=0.81). This indicated that the model was not overdispersed. Nor was it significantly less than 1 (p=0.19). This indicated that the assumption of a Poisson distribution was appropriate for these data. Similarly, there was no relationship between the fitted values and the residuals.

Summary of LMA count analysis:

These models indicated that sensory deprivation had no effect on the number LMAs overall or within any cortical area. The only significant factor in the LMA count was the cortical region.

Effect of sensory deprivation on PA counts

This analysis tests the hypothesis that sensory deprivation (plucking whiskers) affects the distribution of penetrating arterioles (PA).

Hypothesis: Sensory deprivation (whisker plucking) alters the density of PA within individual cortical regions

Null hypothesis (H_0): Sensory deprivation DOES NOT affect the density of PAs in a cortical region.

Alternative hypothesis (H_a): Sensory deprivation DOES affect the density of PAs in a cortical region.

Plot the distribution of all PA counts

```
ggplot(data = Data, mapping = aes(x=NumPA)) +
  geom_histogram(bins = 30, na.rm = TRUE) +
  theme_bw() +
  labs(title = "Distribution of all PA counts:", x = "PA count", y = "Frequency") +
  xlim(0,120)
```

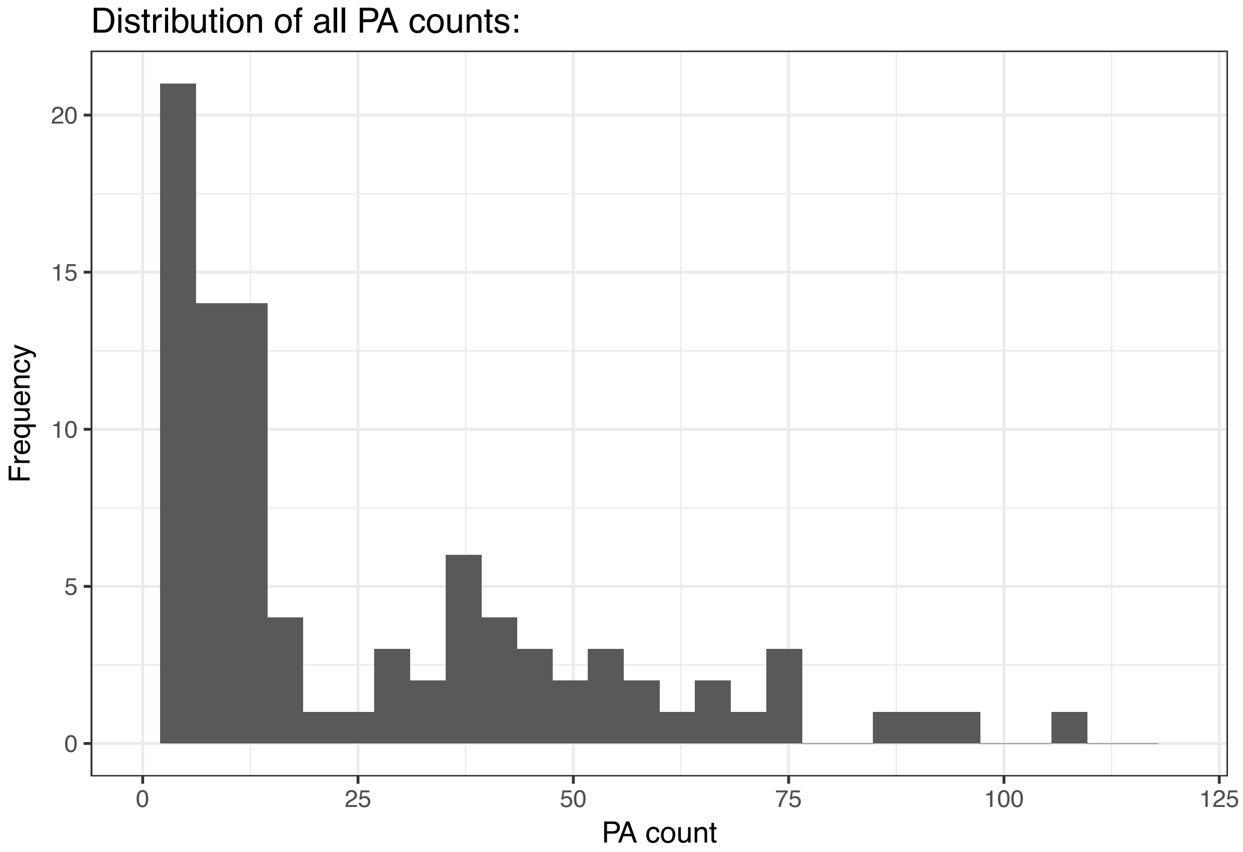

Plot the PA *counts* by Region, Treatment, and Animal

```
ggplot(data = Data, mapping = aes(x=Region, y=NumPA, group = Animal, shape = Sex)) +
  geom_point(na.rm = TRUE, mapping = aes(col = Animal), size = 4) +
  geom_line(na.rm = TRUE, mapping = aes(col = Animal), size = 1) +
  facet_wrap(~Treatment, nrow = 1) +
  labs(title = "PA counts for each animal:", x = "Cortical region", y = "PA count") +
  guides(color = FALSE) +
  theme_bw()
```

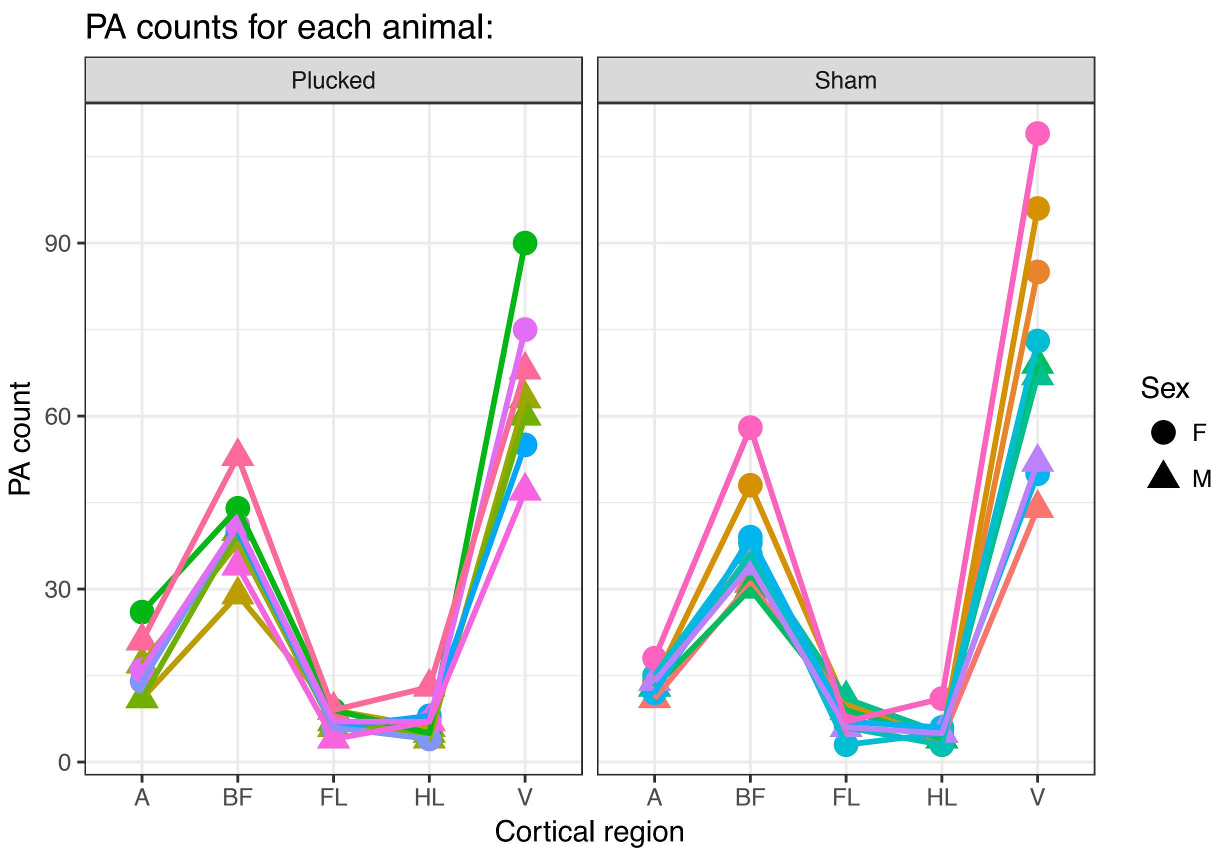

Plot the PA *density* by Region, Treatment, and Animal

```
ggplot(data = Data, mapping = aes(x=Region, y=PADensity, group = Animal, shape = Sex)) +
  geom_point(na.rm = TRUE, mapping = aes(col = Animal), size = 4) +
  geom_line(na.rm = TRUE, mapping = aes(col = Animal), size = 1) +
  facet_wrap(~Treatment, nrow = 1) +
  labs(title = "PA density for each animal:", x = "Cortical region",
       y = "PA density (PA*mm-2)") +
  guides(color = FALSE) +
  theme_bw()
```

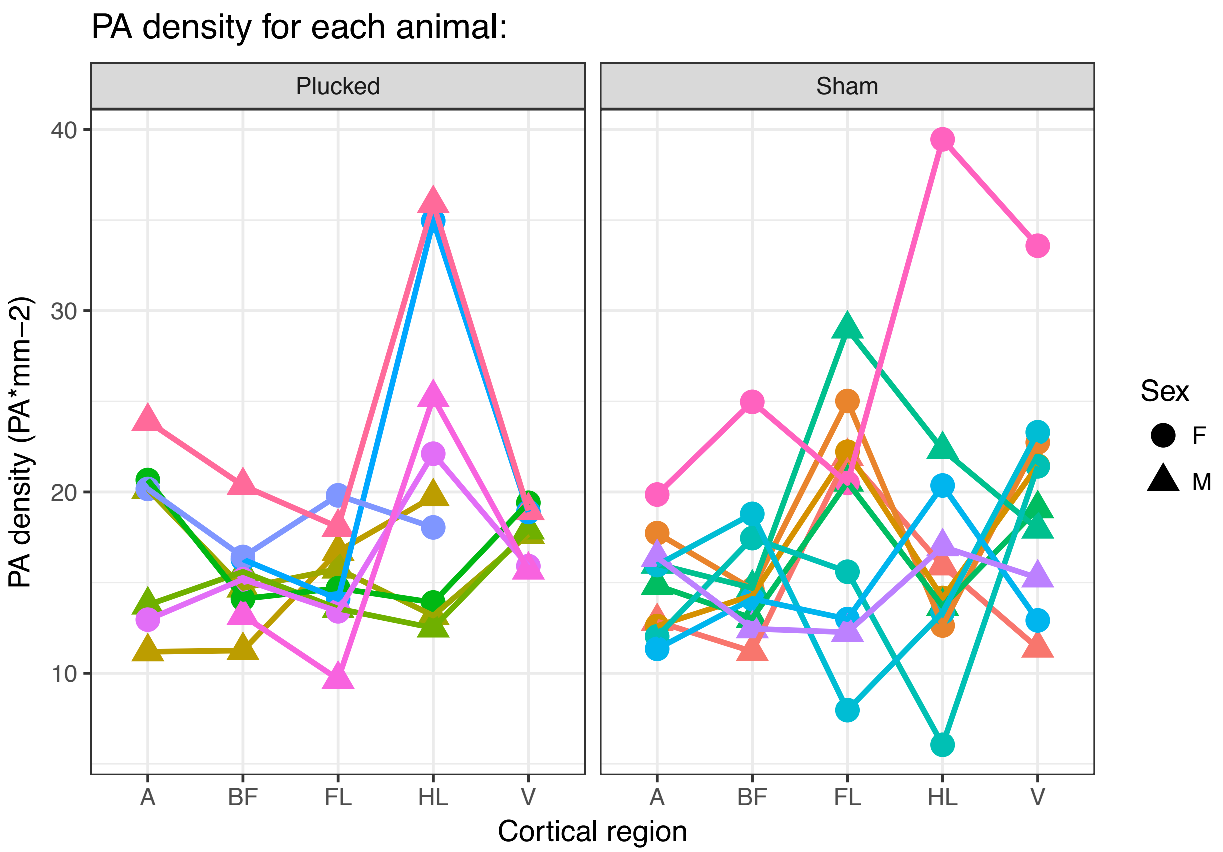

Plot the relationship between Age and total PA count

```
# Build models
PA.Model.Age.All = lm(data = Data.Figs.Complete,
                      PADensity ~ Age_scaled, na.action = na.omit)
Data.Figs.Plucked <- dplyr::filter(Data.Figs.Complete, Treatment == "Plucked")
PA.Model.Age.Plucked = lm(data = Data.Figs.Plucked,
                          PADensity ~ Age_scaled, na.action = na.omit)
Data.Figs.Sham <- dplyr::filter(Data.Figs.Complete, Treatment == "Sham")
PA.Model.Age.Sham = lm(data = Data.Figs.Sham,
                       PADensity ~ Age_scaled, na.action = na.omit)

# Summarize model results in table
PA.Table.Age <- tibble(
  Treatment = c("All","Plucked","Sham"),
  Slope = c(summary(PA.Model.Age.All)$coefficients[2,1],
            summary(PA.Model.Age.Plucked)$coefficients[2,1],
            summary(PA.Model.Age.Sham)$coefficients[2,1]),
  tStat = c(summary(PA.Model.Age.All)$coefficients[2,3],
            summary(PA.Model.Age.Plucked)$coefficients[2,3],
            summary(PA.Model.Age.Sham)$coefficients[2,3]),
  pVal = c(summary(PA.Model.Age.All)$coefficients[2,4],
           summary(PA.Model.Age.Plucked)$coefficients[2,4],
           summary(PA.Model.Age.Sham)$coefficients[2,4])
```

```
  )
kable(PA.Table.Age)
```

Treatment	Slope	tStat	pVal
All	-1.597851	-2.480459	0.0238831
Plucked	-1.410038	-1.720261	0.1290679
Sham	-1.850212	-1.751150	0.1180262

```
# Plot
ggplot(data = Data.Figs.Complete, mapping = aes(x = Age, y = PADensity)) +
  geom_point(mapping = aes(color = Treatment), size = 3) +
  scale_color_manual(values = c("orange","blue")) +
  geom_line(mapping = aes(x = Age, y = fitted(PA.Model.Age.All)),
            color = "black", size = 1) +
  geom_line(Data.Figs.Plucked, mapping = aes(x = Age, y = fitted(PA.Model.Age.Plucked)),
            color = "orange", size = 1) +
  geom_line(Data.Figs.Sham, mapping = aes(x = Age, y = fitted(PA.Model.Age.Sham)),
            color = "blue", size = 1) +
  labs(x = "Age (days)", y = "PA density", title = "Effect of age on PA density:") +
  ylim(10, 30) +
  theme_bw()
```

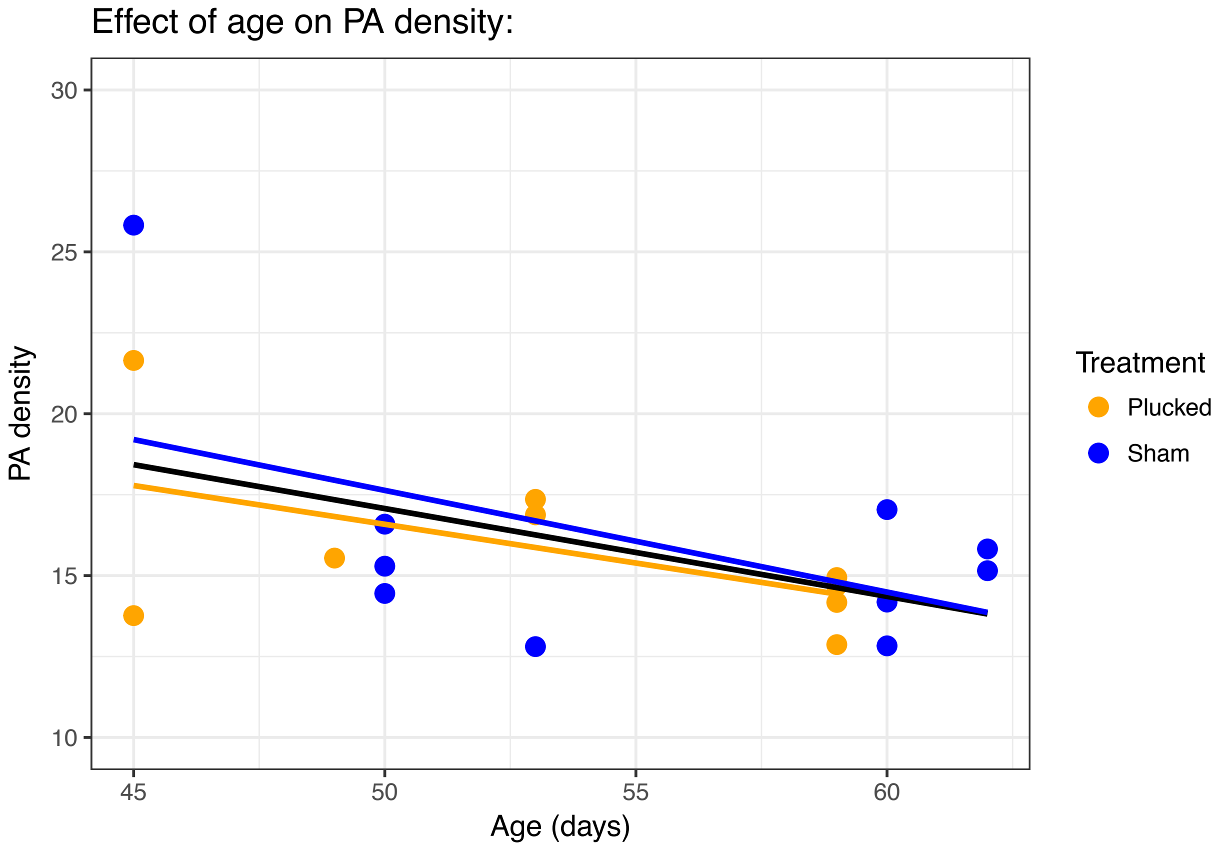


```
ggsave("EffectOfAgeOnNumberOfPAs.eps")
```

```
## Saving 6.5 x 4.5 in image
```

Model the PA counts

```r
PA.Poisson.Full = glmer(NumPA ~ 1 + Age_scaled + Sex + Treatment + Region  +
                          Treatment:Region + Age_scaled:Treatment + (1|Animal) +
                          offset(log(Area)),
                        data = Data, family = poisson,
                        control = glmerControl(optimizer="bobyqa"), na.action = na.omit)
PA.Summary.Poisson.Full = summary(PA.Poisson.Full)
PA.Summary.Poisson.Full
```

```
## Generalized linear mixed model fit by maximum likelihood (Laplace
##   Approximation) [glmerMod]
##  Family: poisson  ( log )
## Formula:
## NumPA ~ 1 + Age_scaled + Sex + Treatment + Region + Treatment:Region +
##     Age_scaled:Treatment + (1 | Animal) + offset(log(Area))
##    Data: Data
## Control: glmerControl(optimizer = "bobyqa")
##
##      AIC      BIC   logLik deviance df.resid
##    541.4    576.6   -256.7    513.4       77
##
## Scaled residuals:
##      Min       1Q   Median       3Q      Max
## -1.85338 -0.53442 -0.00016  0.53880  1.62960
##
## Random effects:
##  Groups Name        Variance Std.Dev.
##  Animal (Intercept) 0.01977  0.1406
## Number of obs: 91, groups:  Animal, 19
##
## Fixed effects:
##                          Estimate Std. Error z value Pr(>|z|)
## (Intercept)              2.912757   0.114288  25.486   <2e-16 ***
## Age_scaled              -0.037765   0.058746  -0.643   0.5203
## SexM                    -0.123392   0.081989  -1.505   0.1323
## TreatmentSham           -0.183534   0.143632  -1.278   0.2013
## RegionBF                -0.136685   0.107691  -1.269   0.2044
## RegionFL                -0.148316   0.156968  -0.945   0.3447
## RegionHL                 0.201240   0.160326   1.255   0.2094
## RegionV                  0.009628   0.106175   0.091   0.9277
## TreatmentSham:RegionBF   0.184202   0.146579   1.257   0.2089
## TreatmentSham:RegionFL   0.394254   0.211502   1.864   0.0623 .
## TreatmentSham:RegionHL  -0.093107   0.230164  -0.405   0.6858
## TreatmentSham:RegionV    0.287380   0.141278   2.034   0.0419 *
## Age_scaled:TreatmentSham -0.028452   0.079923  -0.356   0.7218
## ---
## Signif. codes:  0 '***' 0.001 '**' 0.01 '*' 0.05 '.' 0.1 ' ' 1

##
## Correlation matrix not shown by default, as p = 13 > 12.
## Use print(x, correlation=TRUE)  or
##   vcov(x)     if you need it
```

Comment: Fixed-effects were compared to PA counts in the auditory cortex of sensory deprived mice. The

results indicate there may be an effect of sensory deprivation on the visual cortex. Use likelihood ratios to test this directly.

Perform likelihood ratio tests to test main and interaction effects:

Test the significance of sex on overall PA counts

```
PA.Poisson.Reduced1 = glmer(NumPA ~ 1 + Age_scaled + Treatment + Region  +
                              Treatment:Region + Age_scaled:Treatment + (1|Animal) +
                              offset(log(Area)),
                        data = Data, family = poisson,
                        control = glmerControl(optimizer="bobyqa"), na.action = na.omit)
anova(PA.Poisson.Full, PA.Poisson.Reduced1, test = "Chisq")
```

```
## Data: Data
## Models:
## PA.Poisson.Reduced1: NumPA ~ 1 + Age_scaled + Treatment + Region + Treatment:Region +
## PA.Poisson.Reduced1:     Age_scaled:Treatment + (1 | Animal) + offset(log(Area))
## PA.Poisson.Full: NumPA ~ 1 + Age_scaled + Sex + Treatment + Region + Treatment:Region +
## PA.Poisson.Full:     Age_scaled:Treatment + (1 | Animal) + offset(log(Area))
##                     Df    AIC    BIC  logLik deviance  Chisq Chi Df
## PA.Poisson.Reduced1 13 541.62 574.26 -257.81   515.62
## PA.Poisson.Full     14 541.45 576.60 -256.72   513.45 2.1712      1
##                     Pr(>Chisq)
## PA.Poisson.Reduced1
## PA.Poisson.Full         0.1406
```

Comment: The p-value (p=0.1406) indicated that the model was not significantly improved by inclusion of sex in the model and that sex had no effect on the mean PA counts

Test the significance of sex on overall PA counts

```
PA.Poisson.Reduced2 = glmer(NumPA ~ 1 + Treatment + Region  + Treatment:Region +
                              (1|Animal) + offset(log(Area)),
                        data = Data, family = poisson,
                        control = glmerControl(optimizer="bobyqa"), na.action = na.omit)
anova(PA.Poisson.Reduced1, PA.Poisson.Reduced2, test = "Chisq")
```

```
## Data: Data
## Models:
## PA.Poisson.Reduced2: NumPA ~ 1 + Treatment + Region + Treatment:Region + (1 | Animal) +
## PA.Poisson.Reduced2:     offset(log(Area))
## PA.Poisson.Reduced1: NumPA ~ 1 + Age_scaled + Treatment + Region + Treatment:Region +
## PA.Poisson.Reduced1:     Age_scaled:Treatment + (1 | Animal) + offset(log(Area))
##                     Df    AIC    BIC  logLik deviance  Chisq Chi Df
## PA.Poisson.Reduced2 11 540.48 568.10 -259.24   518.48
## PA.Poisson.Reduced1 13 541.62 574.26 -257.81   515.62 2.8666      2
##                     Pr(>Chisq)
## PA.Poisson.Reduced2
## PA.Poisson.Reduced1     0.2385
```

Comment: The p-value (p=0.2385) indicated that the model was not significantly improved by inclusion of age as a factor in the model and that the age of the mouse did not affect the PA count.

Test the significance of the effect of sensory deprivation on PA counts within cortical regions

```
PA.Poisson.Reduced3 = glmer(NumPA ~ 1 + Treatment + Region + (1|Animal) +
                               offset(log(Area)), data = Data,
                             family = poisson,
                             control = glmerControl(optimizer="bobyqa"),
                             na.action = na.omit)
anova(PA.Poisson.Reduced2, PA.Poisson.Reduced3, test = "Chisq")
```

```
## Data: Data
## Models:
## PA.Poisson.Reduced3: NumPA ~ 1 + Treatment + Region + (1 | Animal) + offset(log(Area))
## PA.Poisson.Reduced2: NumPA ~ 1 + Treatment + Region + Treatment:Region + (1 | Animal) +
## PA.Poisson.Reduced2:     offset(log(Area))
##                     Df    AIC    BIC  logLik deviance  Chisq Chi Df
## PA.Poisson.Reduced3  7 540.35 557.92 -263.17   526.35
## PA.Poisson.Reduced2 11 540.48 568.10 -259.24   518.48 7.8654      4
##                     Pr(>Chisq)
## PA.Poisson.Reduced3
## PA.Poisson.Reduced2    0.09664 .
## ---
## Signif. codes:  0 '***' 0.001 '**' 0.01 '*' 0.05 '.' 0.1 ' ' 1
```

Comment: The p-value (p=0.096) indicated that the model was not significantly improved by including the interaction effects and that the PA counts in cortical regions were not differentially affected by sensory deprivation.

Test the significance of the effect of sensory deprivation on PA count

```
PA.Poisson.Reduced4 = glmer(NumPA ~ 1 + Region + (1|Animal) + offset(log(Area)),
                           data = Data, family = poisson,
                           control = glmerControl(optimizer="bobyqa"),
                           na.action = na.omit)
PA.Summary.Poisson.Reduced4 = summary(PA.Poisson.Reduced4)
anova(PA.Poisson.Reduced4, PA.Poisson.Reduced3, test = "Chisq")
```

```
## Data: Data
## Models:
## PA.Poisson.Reduced4: NumPA ~ 1 + Region + (1 | Animal) + offset(log(Area))
## PA.Poisson.Reduced3: NumPA ~ 1 + Treatment + Region + (1 | Animal) + offset(log(Area))
##                     Df    AIC    BIC  logLik deviance  Chisq Chi Df
## PA.Poisson.Reduced4  6 538.48 553.55 -263.24   526.48
## PA.Poisson.Reduced3  7 540.35 557.92 -263.17   526.35 0.1332      1
##                     Pr(>Chisq)
## PA.Poisson.Reduced4
## PA.Poisson.Reduced3     0.7151
```

Comment: The p-value (p=0.715) indicated that the model was not significantly improved by indluding the overall treatment effects, indicating that the overall PA counts were not affected by sensory deprivation.

Test the significance of the effect of cortical region on PA count

```
PA.Poisson.Reduced5 = glmer(NumPA ~ 1 + (1|Animal) + offset(log(Area)),
                            data = Data, family = poisson,
                            control = glmerControl(optimizer="bobyqa"),
                            na.action = na.omit)
anova(PA.Poisson.Reduced4, PA.Poisson.Reduced5, test = "Chisq")
```

```
## Data: Data
## Models:
## PA.Poisson.Reduced5: NumPA ~ 1 + (1 | Animal) + offset(log(Area))
## PA.Poisson.Reduced4: NumPA ~ 1 + Region + (1 | Animal) + offset(log(Area))
##                     Df    AIC    BIC  logLik deviance  Chisq Chi Df
## PA.Poisson.Reduced5  2 552.90 557.93 -274.45   548.90
## PA.Poisson.Reduced4  6 538.48 553.55 -263.24   526.48 22.423      4
##                     Pr(>Chisq)
## PA.Poisson.Reduced5
## PA.Poisson.Reduced4  0.0001651 ***
## ---
## Signif. codes:  0 '***' 0.001 '**' 0.01 '*' 0.05 '.' 0.1 ' ' 1
```

Comment: The p-value (p=1.651e-4) indicated that the model was improved by including the cortical region as a factor

Use Tukey's honest significance difference-test to determine which regions are different in PA counts

Tukey's HSD evaluated signficance based on a studentized distribution that corrected for multiple comparisons.

```
PA.PostHoc.Region = glht(PA.Poisson.Reduced4, mcp(Region = "Tukey"))
PA.Summary.PostHoc.Region = summary(PA.PostHoc.Region)
PA.Summary.PostHoc.Region
```

```
##
##   Simultaneous Tests for General Linear Hypotheses
##
## Multiple Comparisons of Means: Tukey Contrasts
##
##
## Fit: glmer(formula = NumPA ~ 1 + Region + (1 | Animal) + offset(log(Area)),
##     data = Data, family = poisson, control = glmerControl(optimizer = "bobyqa"),
##     na.action = na.omit)
##
## Linear Hypotheses:
##               Estimate Std. Error z value Pr(>|z|)
## BF - A == 0  -0.031002   0.073001  -0.425   0.9925
## FL - A == 0   0.068901   0.105195   0.655   0.9622
## HL - A == 0   0.171839   0.114614   1.499   0.5398
## V - A == 0    0.178210   0.069849   2.551   0.0719 .
## FL - BF == 0  0.099903   0.091767   1.089   0.7977
## HL - BF == 0  0.202841   0.102543   1.978   0.2570
## V - BF == 0   0.209212   0.047405   4.413   <0.001 ***
## HL - FL == 0  0.102938   0.127467   0.808   0.9216
## V - FL == 0   0.109309   0.089352   1.223   0.7192
```

```
## V - HL == 0   0.006371   0.100316   0.064   1.0000
## ---
## Signif. codes:  0 '***' 0.001 '**' 0.01 '*' 0.05 '.' 0.1 ' ' 1
## (Adjusted p values reported -- single-step method)
```

```
# Extract the estimates for the significant results
PA.SigCoef.PostHoc.Region <-
  PA.Summary.PostHoc.Region$test$coefficients[PA.Summary.PostHoc.Region$test$pvalues < 0.05]

# Extract the effect size for the signficant results
PA.SigZ.PostHoc.Region <-
  PA.Summary.PostHoc.Region$test$tstat[PA.Summary.PostHoc.Region$test$pvalues < 0.05]

#Get the significant p-values
PA.SigP.PostHoc.Region <-
  PA.Summary.PostHoc.Region$test$pvalues[PA.Summary.PostHoc.Region$test$pvalues < 0.05]

plot(PA.PostHoc.Region, xlab = "Difference in log(mean) of LMA")
```

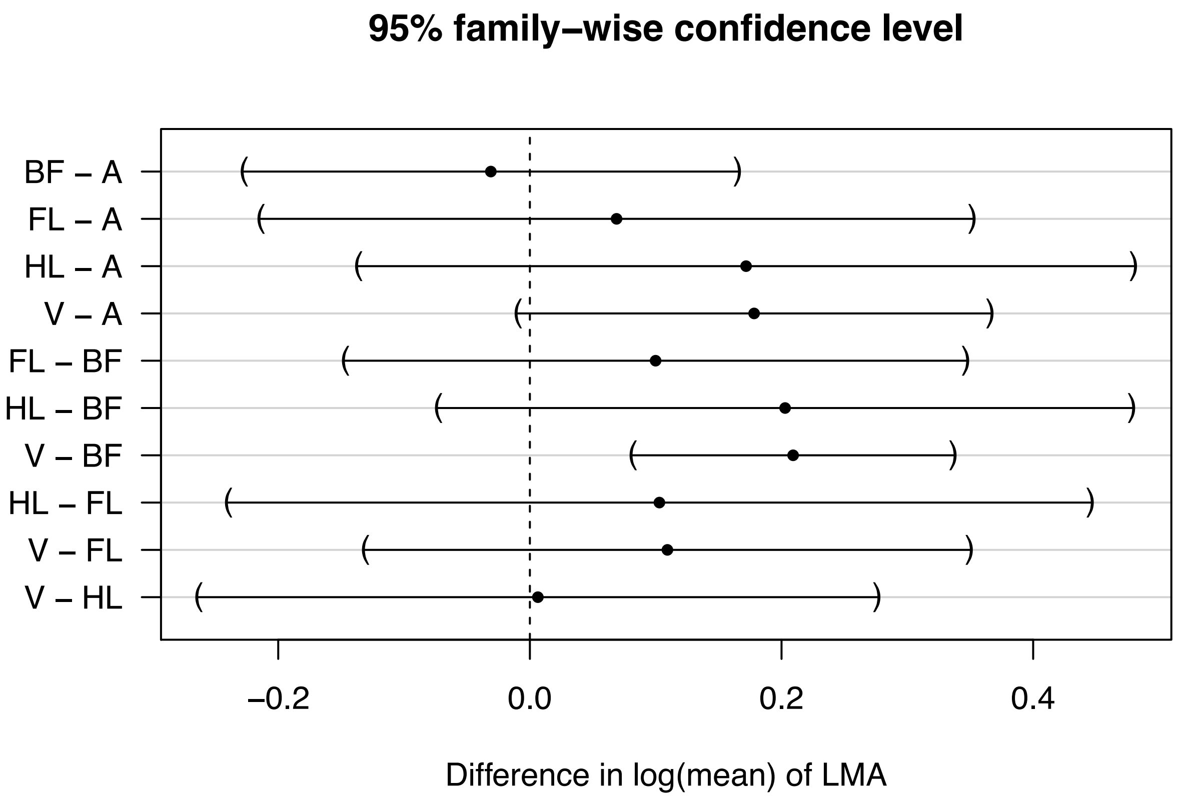

Comment: the visual and barrel cortex were different in PA counts although the difference was not drastic (1.22 PA/mm^2)

Test model assumptions

```
# Get the degrees of freedom for the residuals (rdf)
rdf <- PA.Summary.Poisson.Reduced4$AICtab[[5]]
```

```r
# Get the studentized residuals of the model
rp <- residuals(PA.Poisson.Reduced4,type="pearson")

# Calculate the variance of the studentized residuals
rp_var <- sum(rp^2)

# Show the ratio of error variance, mean
rat = rp_var/rdf

# Get the probability (pval) that the variance > mean
pval_over <- pchisq(rp_var, df = rdf, lower.tail = FALSE)
pval_under <- pchisq(rp_var, df = rdf, lower.tail = TRUE)

# Summarize
disp_summary = c(Residual_Variance = rp_var, Ratio = rat, df = rdf,
                 p_over = pval_over, p_under = pval_under)
disp_summary
```

```
## Residual_Variance             Ratio                df            p_over
##       59.47415597        0.69969595       85.00000000        0.98401561
##           p_under
##        0.01598439
```

```r
# Calculate the studentized residuals
Resids = data.frame(Mouse_Number = Data[complete.cases(Data$NumPA),]$Animal,
                    Fitted_Value = fitted(PA.Poisson.Reduced4),
                    Standardized_Resids = residuals(PA.Poisson.Reduced4, type="pearson"))

# Fit the plot with a line to test whether slope = 0
Resids.Fitted = lm(Standardized_Resids~Fitted_Value, data = Resids)

# Plot the studentized residual amplitude vs. the fitted value
plot(PA.Poisson.Reduced4)
```

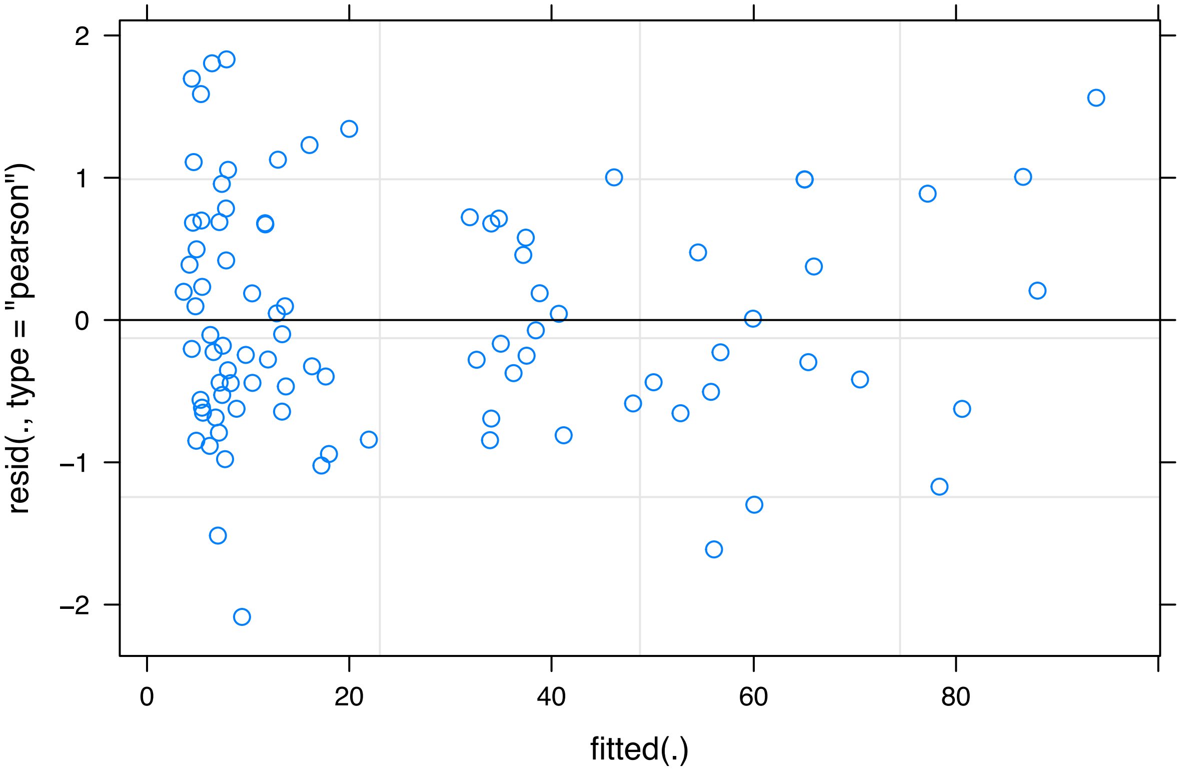


```
# Test the significance of the slope
anova(Resids.Fitted)
```

```
## Analysis of Variance Table
##
## Response: Standardized_Resids
##               Df Sum Sq Mean Sq F value Pr(>F)
## Fitted_Value  1  0.001 0.00141  0.0021 0.9634
## Residuals    89 59.455 0.66804
```

Comment: The ratio $\frac{Var_{error}}{mean_{error}} = \frac{73.54}{85} = 0.984$ was not significantly greater than 1 (p=0.98). This indicated that the model was not overdispersed. However, it was significantly less than 1 (p=0.015, 0.03 [bonferroni corrected]). This indicated that the data are slightly under-dispersed. However, examination of the fitted vs. residual plot indicated that this effect was subtle.

Summary of PA count analysis:

These models indicated that sensory deprivation had no effect on the number of PAs overall or within any cortical area. The only significant factor in the LMA count was the cortical region.

Effect of sensory deprivation on vessel branching structure

Branching structure can be evaluated by the amount of branches (vertices) per vessel. This analysis tested whether sensory deprivation altered the branching structure.

Plot the distribution offshoots per vessel

```
ggplot(data = Data, mapping = aes(x=AvgNumVertInBranch)) +
  geom_histogram(bins = 15, na.rm = TRUE) +
  theme_bw() +
  xlim(0,10) +
  labs(title = "Distribution of all branch densities:", x = "Vertices per offshoot",
       y = "Frequency")
```

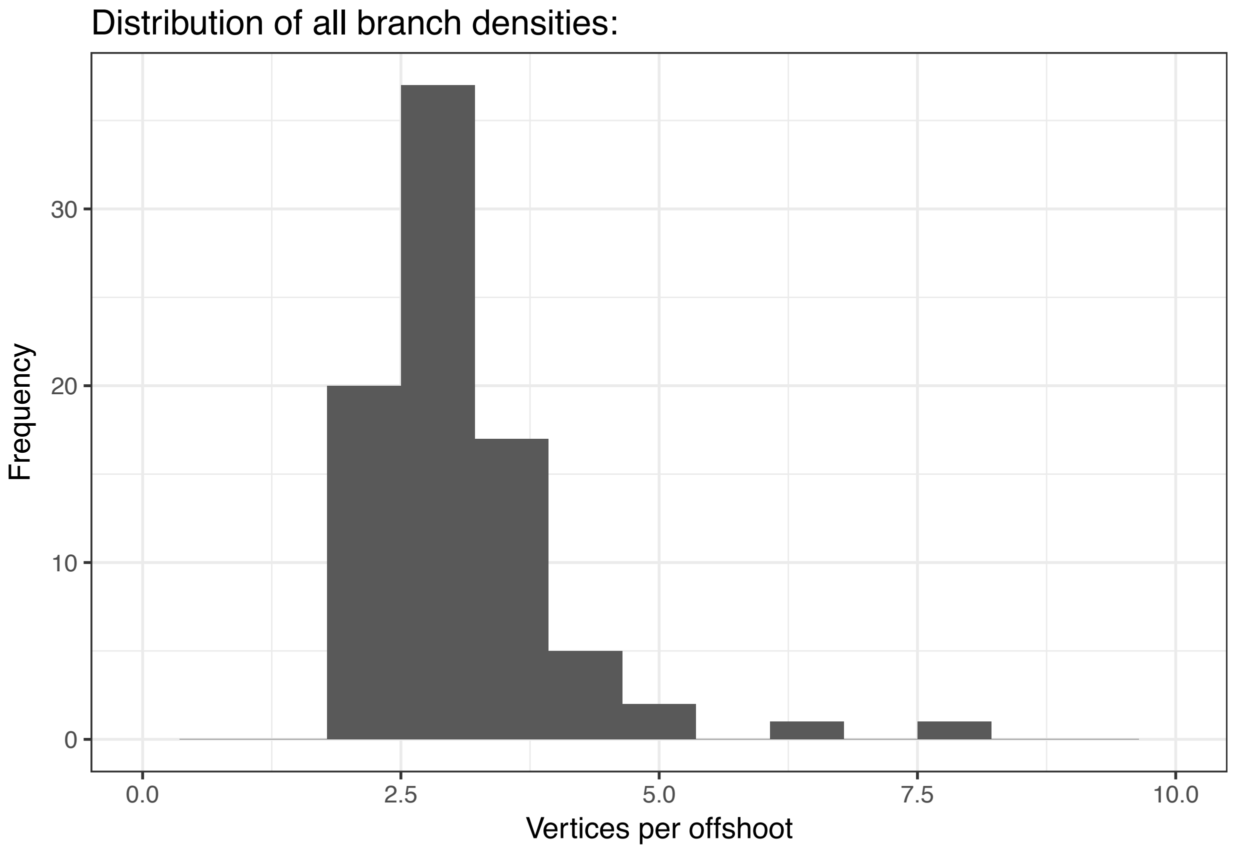

Comment: These data were not normal and were continuous. We therefore used a GLM based on a Gamma-distribution.

Plot the average number of vertices per offshoot by region, treatment, and animal

```
ggplot(data = Data, mapping = aes(x=Region, y=AvgNumVertInBranch, group = Animal)) +
  geom_point(na.rm = TRUE) +
  geom_line(na.rm = TRUE, mapping = aes(col = Animal)) +
  facet_wrap(~Treatment, nrow = 1) +
  labs(title = "Avg # Vertices per offshoot for each animal:", x = "Cortical region",
       y = "Avg # Vertices per offshoot") +
  guides(color = FALSE) +
  theme_bw()
```

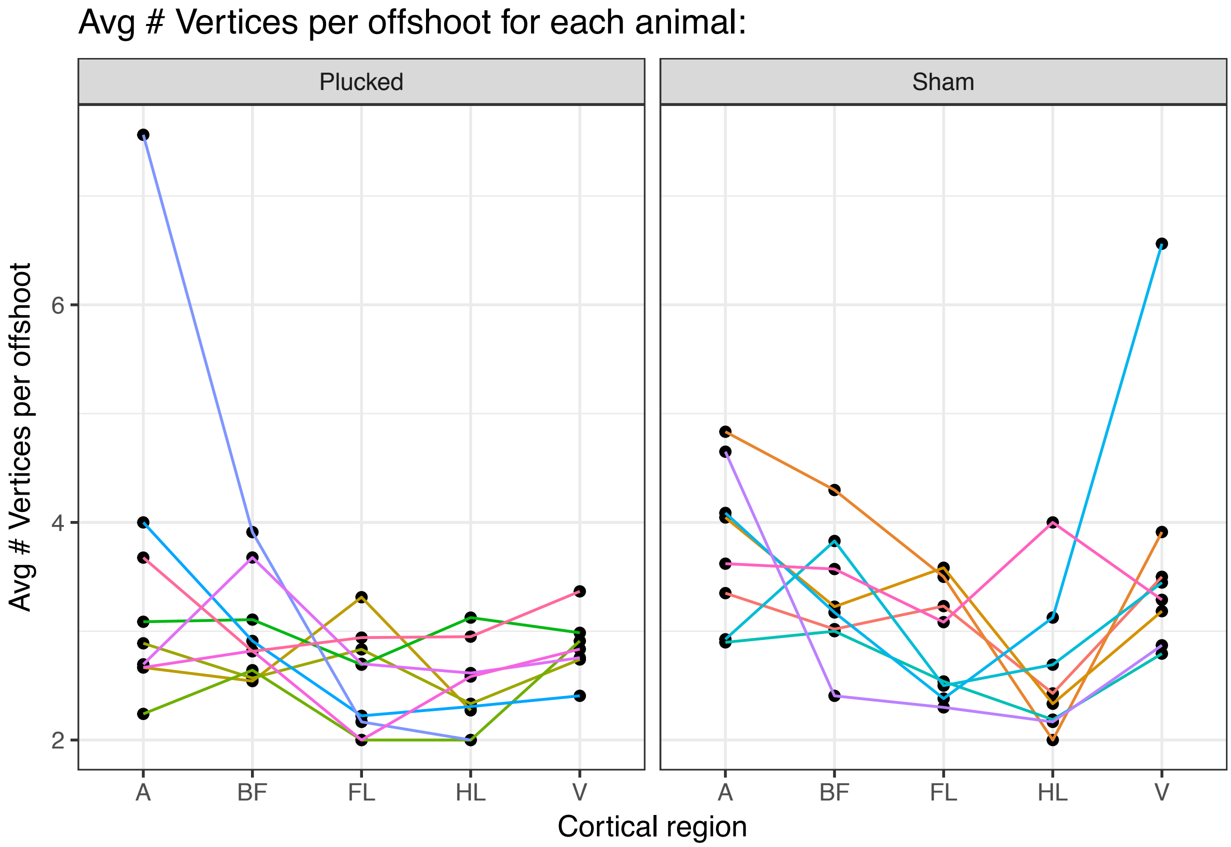

Model build

```
Vert.Gamma.Max = glmer(AvgNumVertInBranch ~ 1 + Treatment*Region + (1|Animal),
                        data = Data, family = Gamma,
                        control = glmerControl(optimizer="bobyqa"), na.action = na.omit)
Vert.Summary.Gamma.Max = summary(Vert.Gamma.Max)
Vert.Summary.Gamma.Max
```

```
## Generalized linear mixed model fit by maximum likelihood (Laplace
##   Approximation) [glmerMod]
##  Family: Gamma  ( inverse )
## Formula: AvgNumVertInBranch ~ 1 + Treatment * Region + (1 | Animal)
##    Data: Data
## Control: glmerControl(optimizer = "bobyqa")
##
##      AIC      BIC   logLik deviance df.resid
##    168.1    197.1    -72.0    144.1       71
##
## Scaled residuals:
##     Min      1Q  Median      3Q     Max
## -1.4765 -0.6266 -0.1040  0.4944  4.0012
##
## Random effects:
##  Groups   Name        Variance Std.Dev.
##  Animal   (Intercept) 0.001038 0.03222
```

```
##  Residual             0.038826 0.19704
## Number of obs: 83, groups:  Animal, 17
##
## Fixed effects:
##                          Estimate Std. Error t value Pr(>|z|)
## (Intercept)              0.293733   0.023526  12.486  < 2e-16 ***
## TreatmentSham           -0.023408   0.033768  -0.693 0.488186
## RegionBF                 0.046720   0.025475   1.834 0.066658 .
## RegionFL                 0.106098   0.028276   3.752 0.000175 ***
## RegionHL                 0.118048   0.028857   4.091  4.3e-05 ***
## RegionV                  0.051748   0.029076   1.780 0.075122 .
## TreatmentSham:RegionBF -0.008676   0.035456  -0.245 0.806681
## TreatmentSham:RegionFL -0.024093   0.038970  -0.618 0.536406
## TreatmentSham:RegionHL -0.000246   0.040662  -0.006 0.995173
## TreatmentSham:RegionV  -0.044276   0.037217  -1.190 0.234177
## ---
## Signif. codes:  0 '***' 0.001 '**' 0.01 '*' 0.05 '.' 0.1 ' ' 1
##
## Correlation of Fixed Effects:
##             (Intr) TrtmnS RegnBF RegnFL RegnHL ReginV TS:RBF TS:RFL TS:RHL
## TreatmntShm -0.686
## RegionBF    -0.458  0.319
## RegionFL    -0.414  0.287  0.381
## RegionHL    -0.406  0.282  0.373  0.336
## RegionV     -0.419  0.286  0.372  0.337  0.331
## TrtmntS:RBF  0.329 -0.448 -0.718 -0.273 -0.268 -0.267
## TrtmntS:RFL  0.300 -0.407 -0.276 -0.725 -0.244 -0.244  0.388
## TrtmntS:RHL  0.287 -0.390 -0.265 -0.239 -0.710 -0.234  0.372  0.339
## TrtmntSh:RV  0.327 -0.432 -0.291 -0.264 -0.258 -0.781  0.407  0.372  0.356
```

Comment: The fixed-effects were compared to branches per vessel in auditory cortex of sensory deprived mice. They indicated a possible difference in branching structure between cortical region. Directly tested the fixed-effects using the likelihood ratio test.

Perform likelihood ratio tests to test main and interaction effects:

Test the significance of the effect of sensory deprivation on vertices per offshoot within cortical regions

```
Vert.Gamma.NoInteraction = glmer(AvgNumVertInBranch ~ 1 + Treatment + Region + (1|Animal),
                                 data = Data, family = Gamma,
                                 control = glmerControl(optimizer="bobyqa"),
                                 na.action = na.omit)
anova(Vert.Gamma.Max, Vert.Gamma.NoInteraction, test = "Chisq")
```

```
## Data: Data
## Models:
## Vert.Gamma.NoInteraction: AvgNumVertInBranch ~ 1 + Treatment + Region + (1 | Animal)
## Vert.Gamma.Max: AvgNumVertInBranch ~ 1 + Treatment * Region + (1 | Animal)
##                          Df    AIC    BIC  logLik deviance  Chisq Chi Df
## Vert.Gamma.NoInteraction  8 161.83 181.18 -72.915   145.83
## Vert.Gamma.Max           12 168.08 197.10 -72.039   144.08 1.7519      4
##                          Pr(>Chisq)
```

```
## Vert.Gamma.NoInteraction
## Vert.Gamma.Max               0.7813
```

Comment: The p-value (p=0.781) indicated that the model of branching was not significantly improved by including the interaction between sensory deprivation and cortical region and that sensory deprivation did not differentially affect the branching in any cortical area

Test the significance of the effect of cortical region on vertices per offshoot

```
Vert.Gamma.Treatment = glmer(AvgNumVertInBranch ~ 1 + Treatment + (1|Animal),
                             data = Data, family = Gamma,
                             control = glmerControl(optimizer="bobyqa"),
                             na.action = na.omit)
Summary.Gamma.Treatment = summary(Vert.Gamma.Treatment)
anova(Vert.Gamma.NoInteraction, Vert.Gamma.Treatment, test = "Chisq")
```

```
## Data: Data
## Models:
## Vert.Gamma.Treatment: AvgNumVertInBranch ~ 1 + Treatment + (1 | Animal)
## Vert.Gamma.NoInteraction: AvgNumVertInBranch ~ 1 + Treatment + Region + (1 | Animal)
##                          Df    AIC    BIC  logLik deviance  Chisq Chi Df
## Vert.Gamma.Treatment      4 189.73 199.41 -90.867   181.73
## Vert.Gamma.NoInteraction  8 161.83 181.18 -72.915   145.83 35.903      4
##                          Pr(>Chisq)
## Vert.Gamma.Treatment
## Vert.Gamma.NoInteraction   3.03e-07 ***
## ---
## Signif. codes:  0 '***' 0.001 '**' 0.01 '*' 0.05 '.' 0.1 ' ' 1
```

Comment: The p-value (p=3.03e-07) indicated that the model was improved by including the cortical region as a factor.

Test the significance of the treatment on vertices per offshoot

```
Vert.Gamma.Null = glmer(AvgNumVertInBranch ~ 1 + (1|Animal),
                        data = Data, family = Gamma,
                        control = glmerControl(optimizer = "bobyqa"),
                        na.action = na.omit)
Summary.Gamma.Null = summary(Vert.Gamma.Null)
anova(Vert.Gamma.Treatment, Vert.Gamma.Null, test = "Chisq")
```

```
## Data: Data
## Models:
## Vert.Gamma.Null: AvgNumVertInBranch ~ 1 + (1 | Animal)
## Vert.Gamma.Treatment: AvgNumVertInBranch ~ 1 + Treatment + (1 | Animal)
##                      Df    AIC    BIC  logLik deviance  Chisq Chi Df
## Vert.Gamma.Null       3 189.59 196.85 -91.795   183.59
## Vert.Gamma.Treatment  4 189.73 199.41 -90.867   181.73 1.8569      1
##                      Pr(>Chisq)
## Vert.Gamma.Null
## Vert.Gamma.Treatment      0.173
```

Comment: The p-value (p=0.173) indicated that the model was not improved by including sensory deprivation as a factor and that sensory deprivation did not alter vessel branching overall.

Use Tukey's honest significance difference-test to test which regions are different in branches per vessel

Tukey's HSD evaluates signficance based on a studentized distribution that corrects for multiple comparisons.

```
Vert.Gamma.Region = glmer(AvgNumVertInBranch ~ 1 + Region + (1|Animal),
                              data = Data, family = Gamma,
                              control = glmerControl(optimizer="bobyqa"),
                              na.action = na.omit)
Vert.PostHoc.Region = glht(Vert.Gamma.Region, mcp(Region = "Tukey"))

Vert.Summary.PostHoc.Region = summary(Vert.PostHoc.Region)
Vert.Summary.PostHoc.Region
```

```
##
##   Simultaneous Tests for General Linear Hypotheses
##
## Multiple Comparisons of Means: Tukey Contrasts
##
##
## Fit: glmer(formula = AvgNumVertInBranch ~ 1 + Region + (1 | Animal),
##     data = Data, family = Gamma, control = glmerControl(optimizer = "bobyqa"),
##     na.action = na.omit)
##
## Linear Hypotheses:
##               Estimate Std. Error z value Pr(>|z|)
## BF - A == 0    0.04213    0.02065   2.040  0.24500
## FL - A == 0    0.09336    0.02263   4.126  < 0.001 ***
## HL - A == 0    0.11761    0.02360   4.984  < 0.001 ***
## V - A == 0     0.02386    0.02093   1.140  0.78377
## FL - BF == 0   0.05123    0.02393   2.140  0.20139
## HL - BF == 0   0.07548    0.02485   3.037  0.02004 *
## V - BF == 0   -0.01827    0.02233  -0.818  0.92451
## HL - FL == 0   0.02426    0.02651   0.915  0.89036
## V - FL == 0   -0.06949    0.02417  -2.876  0.03242 *
## V - HL == 0   -0.09375    0.02508  -3.739  0.00171 **
## ---
## Signif. codes:  0 '***' 0.001 '**' 0.01 '*' 0.05 '.' 0.1 ' ' 1
## (Adjusted p values reported -- single-step method)
```

```
# Extract the estimates for the significant results
Vert.SigCoef.PostHoc.Region <-
  Vert.Summary.PostHoc.Region$test$coefficients[Vert.Summary.PostHoc.Region$test$pvalues < 0.05]

# Extract the effect size for the signficant results
Vert.SigZ.PostHoc.Region <-
  Vert.Summary.PostHoc.Region$test$tstat[Vert.Summary.PostHoc.Region$test$pvalues < 0.05]

#Get the significant p-values
Vert.SigP.PostHoc.Region <-
  Vert.Summary.PostHoc.Region$test$pvalues[Vert.Summary.PostHoc.Region$test$pvalues < 0.05]

summary(Vert.PostHoc.Region)
```

```
##
```

```
##   Simultaneous Tests for General Linear Hypotheses
##
## Multiple Comparisons of Means: Tukey Contrasts
##
##
## Fit: glmer(formula = AvgNumVertInBranch ~ 1 + Region + (1 | Animal),
##     data = Data, family = Gamma, control = glmerControl(optimizer = "bobyqa"),
##     na.action = na.omit)
##
## Linear Hypotheses:
##              Estimate Std. Error z value Pr(>|z|)
## BF - A == 0   0.04213    0.02065   2.040  0.24495
## FL - A == 0   0.09336    0.02263   4.126  < 0.001 ***
## HL - A == 0   0.11761    0.02360   4.984  < 0.001 ***
## V - A == 0    0.02386    0.02093   1.140  0.78380
## FL - BF == 0  0.05123    0.02393   2.140  0.20126
## HL - BF == 0  0.07548    0.02485   3.037  0.02001 *
## V - BF == 0  -0.01827    0.02233  -0.818  0.92450
## HL - FL == 0  0.02426    0.02651   0.915  0.89035
## V - FL == 0  -0.06949    0.02417  -2.876  0.03248 *
## V - HL == 0  -0.09375    0.02508  -3.739  0.00171 **
## ---
## Signif. codes:  0 '***' 0.001 '**' 0.01 '*' 0.05 '.' 0.1 ' ' 1
## (Adjusted p values reported -- single-step method)
```

```r
plot(Vert.PostHoc.Region, xlab = "Difference in log(mean) of LMA")
```

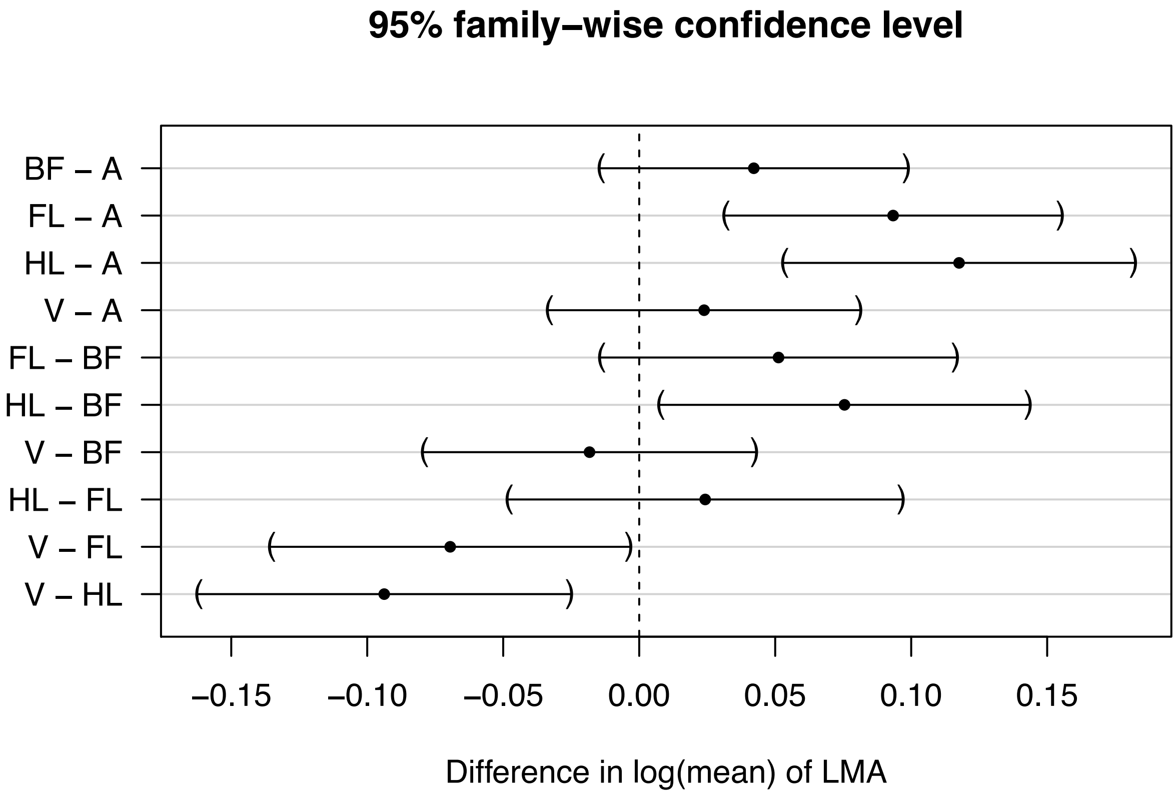

Comment: The branching structure in forelimb/hindlimb regions were different from visual, barrel, and auditory cortex.

Check that error is independent of the fitted value

```
# Calculate the studentized residuals
Resids = data.frame(Mouse_Number = Data[complete.cases(Data$AvgNumVertInBranch),]$Animal,
                    Fitted_Value = fitted(Vert.Gamma.Region),
                    Standardized_Resids = residuals(Vert.Gamma.Region, type="pearson"))

# Fit the plot with a line to test whether slope = 0
Resids.Fitted = lm(Standardized_Resids~Fitted_Value, data = Resids)

# Plot the studentized residual amplitude vs. the fitted value
plot(Vert.Gamma.Region)
```

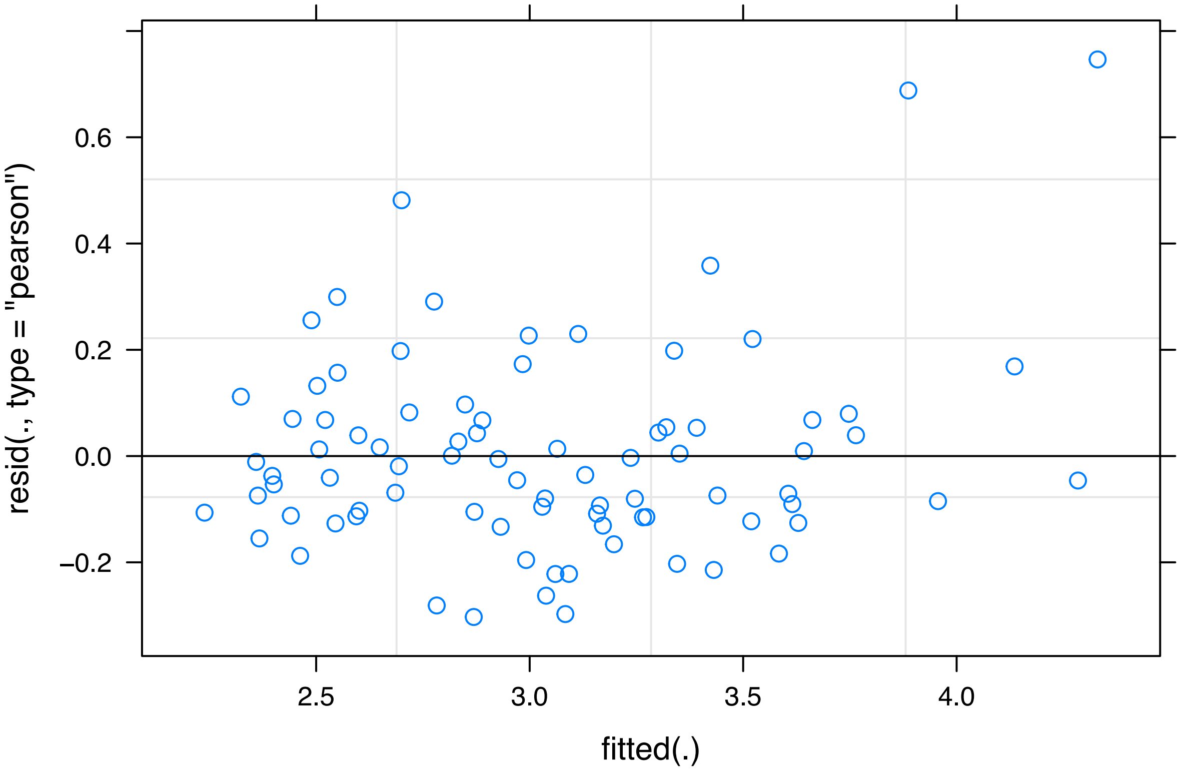


```
# Test the significance of the slope
anova(Resids.Fitted)
```

```
## Analysis of Variance Table
##
## Response: Standardized_Resids
##              Df  Sum Sq  Mean Sq F value Pr(>F)
## Fitted_Value  1 0.08484 0.084837  2.3914 0.1259
## Residuals    81 2.87360 0.035476
```

Comment: There was no significant slope between the fitted values and residuals, indicating the the Gamma-distribution appropriately captured the mean-variance structure.

Summary of the analysis of branching structure:

These models indicate that sensory deprivation had no effect on the overall branching structure or the branching structure within any cortical area. The only significant factor in the LMA count was the cortical region.

Summary Table

```
# Gather data for table
TabData <- tibble(
  Measurement = c(rep("LMACount",length(LMA.SigCoef.PostHoc.Region)),
            rep("PACount",length(PA.SigCoef.PostHoc.Region)),
            rep("BranchPerVessel",length(Vert.SigCoef.PostHoc.Region))),
  RegionComparison = c(names(LMA.SigCoef.PostHoc.Region),
               names(PA.SigCoef.PostHoc.Region),
               names(Vert.SigCoef.PostHoc.Region)),
  Difference = c(exp(LMA.SigCoef.PostHoc.Region),
               exp(PA.SigCoef.PostHoc.Region),
               exp(Vert.SigCoef.PostHoc.Region)),
  EffectSize = c(LMA.SigZ.PostHoc.Region,
               PA.SigZ.PostHoc.Region,
               Vert.SigZ.PostHoc.Region),
  PValue = c(LMA.SigP.PostHoc.Region,
           PA.SigP.PostHoc.Region,
           Vert.SigP.PostHoc.Region)
  )
kable(TabData)
```

Measurement	RegionComparison	Difference	EffectSize	PValue
LMACount	HL - A	3.8238080	4.338214	0.0001619
LMACount	HL - BF	4.9050442	6.322428	0.0000000
LMACount	V - BF	2.2672926	4.622904	0.0000329
LMACount	HL - FL	4.0301694	3.585437	0.0027877
LMACount	V - HL	0.4622369	-3.518452	0.0036118
PACount	V - BF	1.2327068	4.413329	0.0000948
BranchPerVessel	FL - A	1.0978522	4.125771	0.0003526
BranchPerVessel	HL - A	1.1248075	4.984126	0.0000044
BranchPerVessel	HL - BF	1.0784032	3.037364	0.0200439
BranchPerVessel	V - FL	0.9328649	-2.875664	0.0324197
BranchPerVessel	V - HL	0.9105094	-3.738812	0.0017144

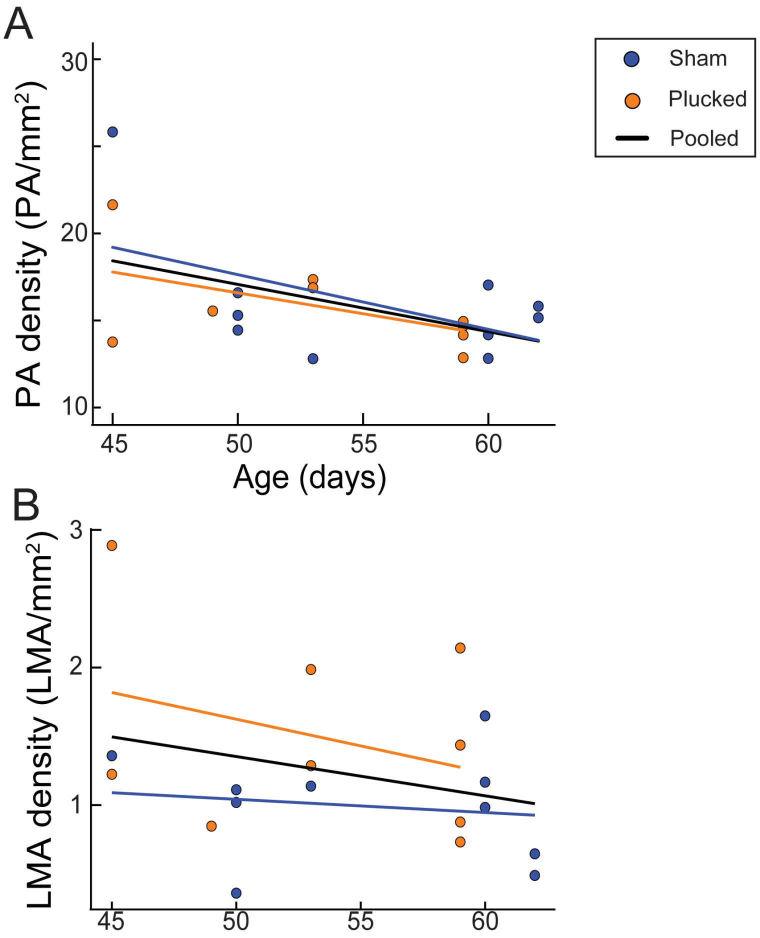

Supplementary Figure 1: Effect of age at time of perfusion on the number of PAs and LMAs. (A) Age versus number of penetrating arterioles. Age had no detectable effect on the number of PAs (Likelihood ratio test, p=0.24, $\chi^2(2) = 0.87$). (Sham: n = 10, Plucked: n = 9, Linear regression fit by least squares: Pooled: p=0.07 (Bonferroni corrected), t(17)=-2.48; Plucked: p = 0.13, t(7)=-1.72; Sham: p=0.12, t(8)=-1.75). (B) Age vs the number of leptomeningeal anastomoses. Age has no significant effect on the number of LMAs at the time of perfusion (Likelihood ratio test, p=0.66, χ^2=0.83). (Sham: n = 10, Plucked n = 7, Linear regression: Pooled: p=0.24, t(17) = -1.21; Plucked: p=0.39, t(7)=-0.91; Sham: p=0.68, t(8)=-0.44, (Bonferroni corrected)).

Supplementary data set 1: All pial arterial reconstructions used in this study. The backbone of the MCA from the left hemispheres used in this study are shown in dark red, PAs in red, and LMAs in green. The cortical regions are shown in blue. The Voronoi cells are shown in orange, plotted with a PA at the center of each cell. The watershed line can be defined two different ways, manually, or by assigning the Voronoi polygons surrounding the individual PAs to a vascular territory. The purple line shows the manually drawn watershed line which bisects the LMAs. The dark orange line denotes the boundary obtained by assigning the territory perfused by each PA to the MCA or the ACA/PCA. The MCA territory will be below the watershed line(s) in these schematics, the PCA/ACA territory will be above it.

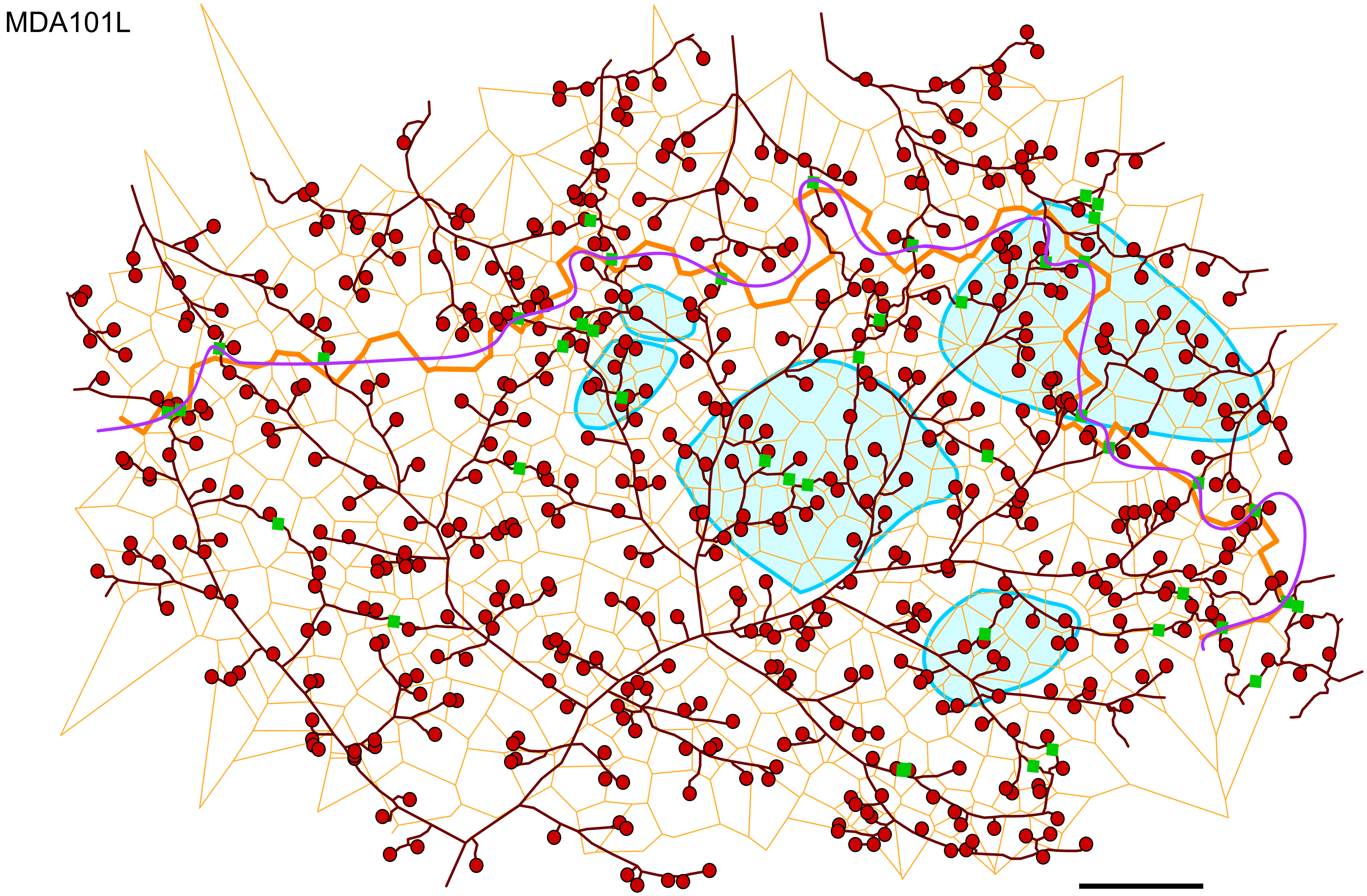
MDA101L

MDA105L

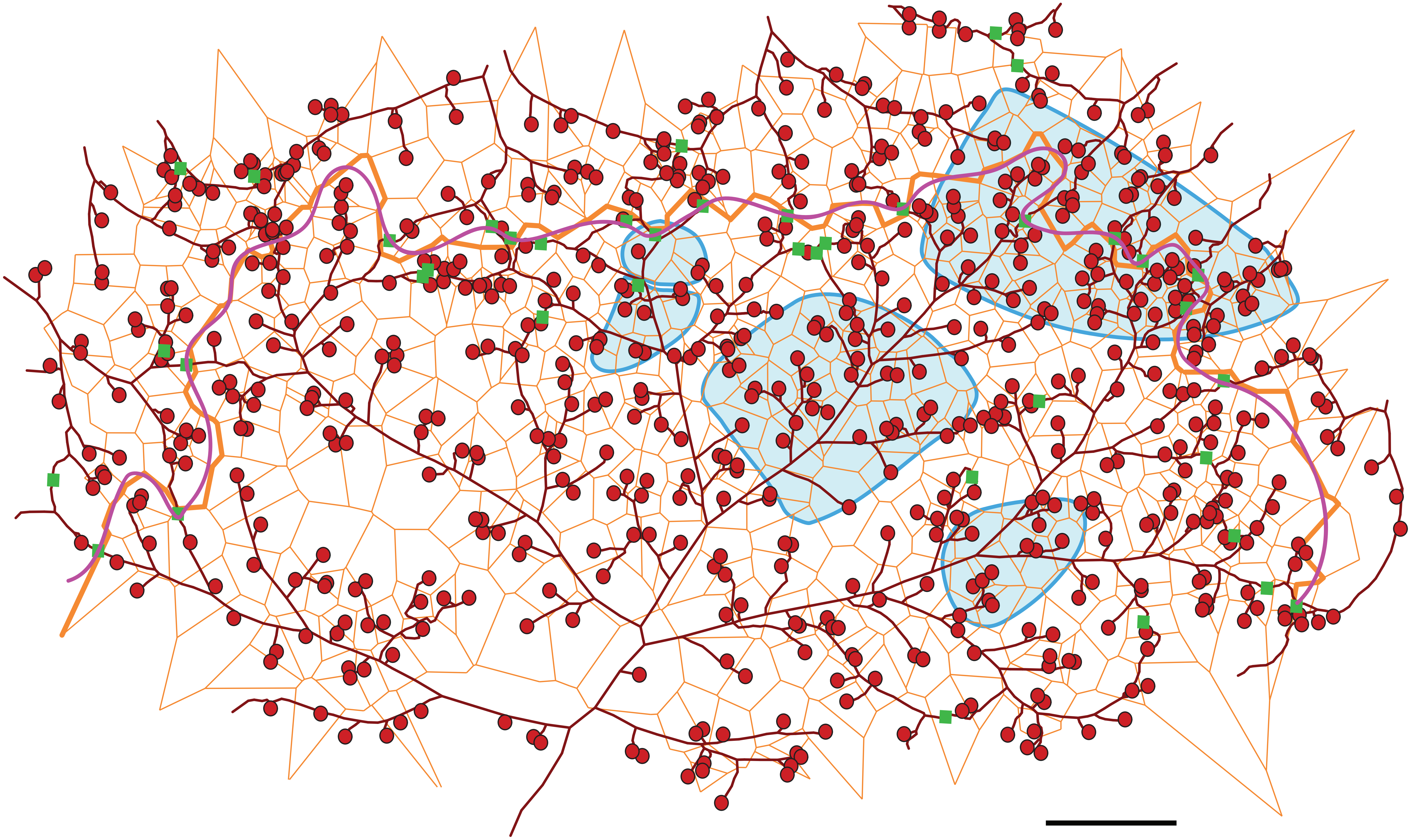

MDA106L

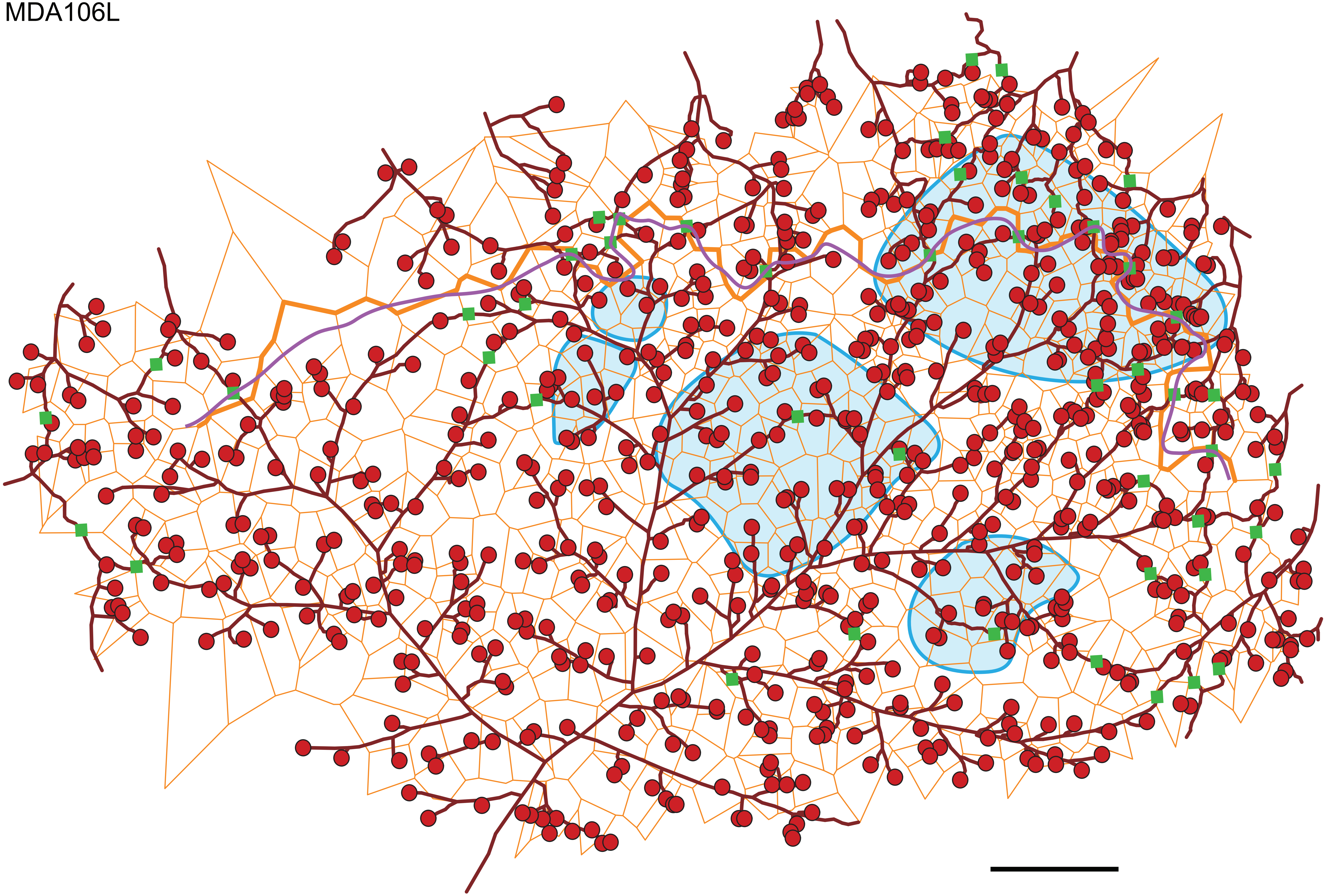

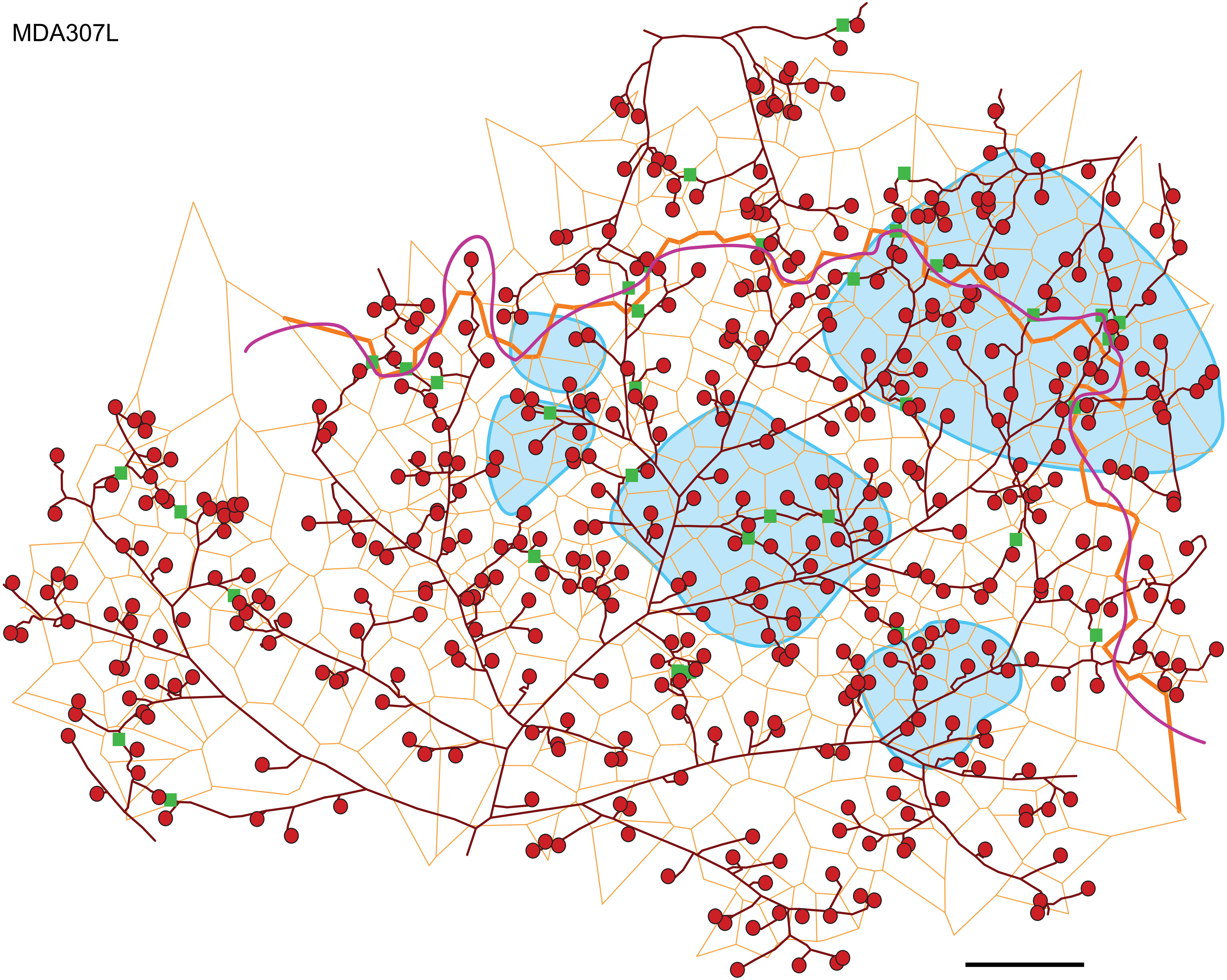
MDA307L

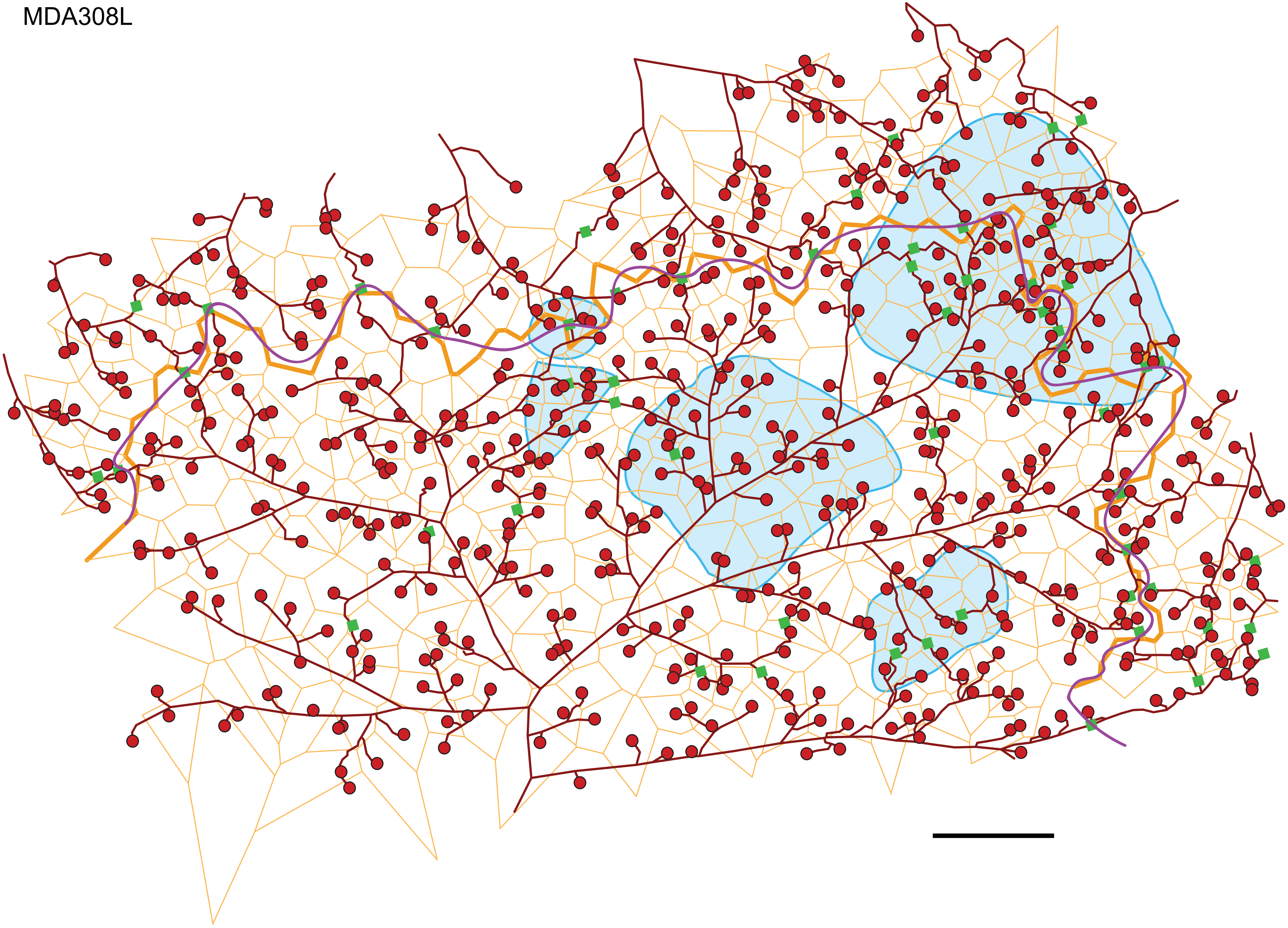
MDA308L

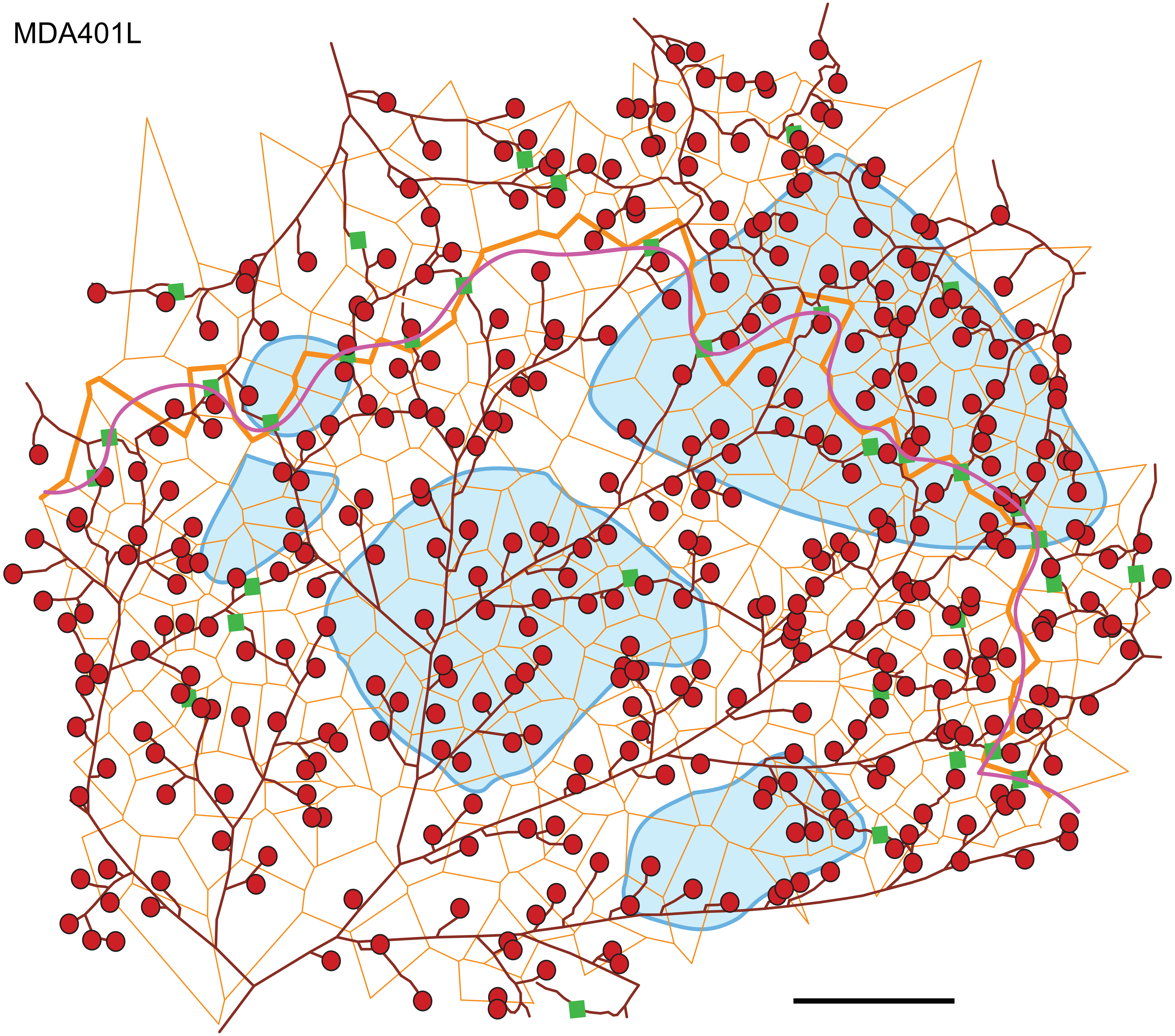
MDA401L

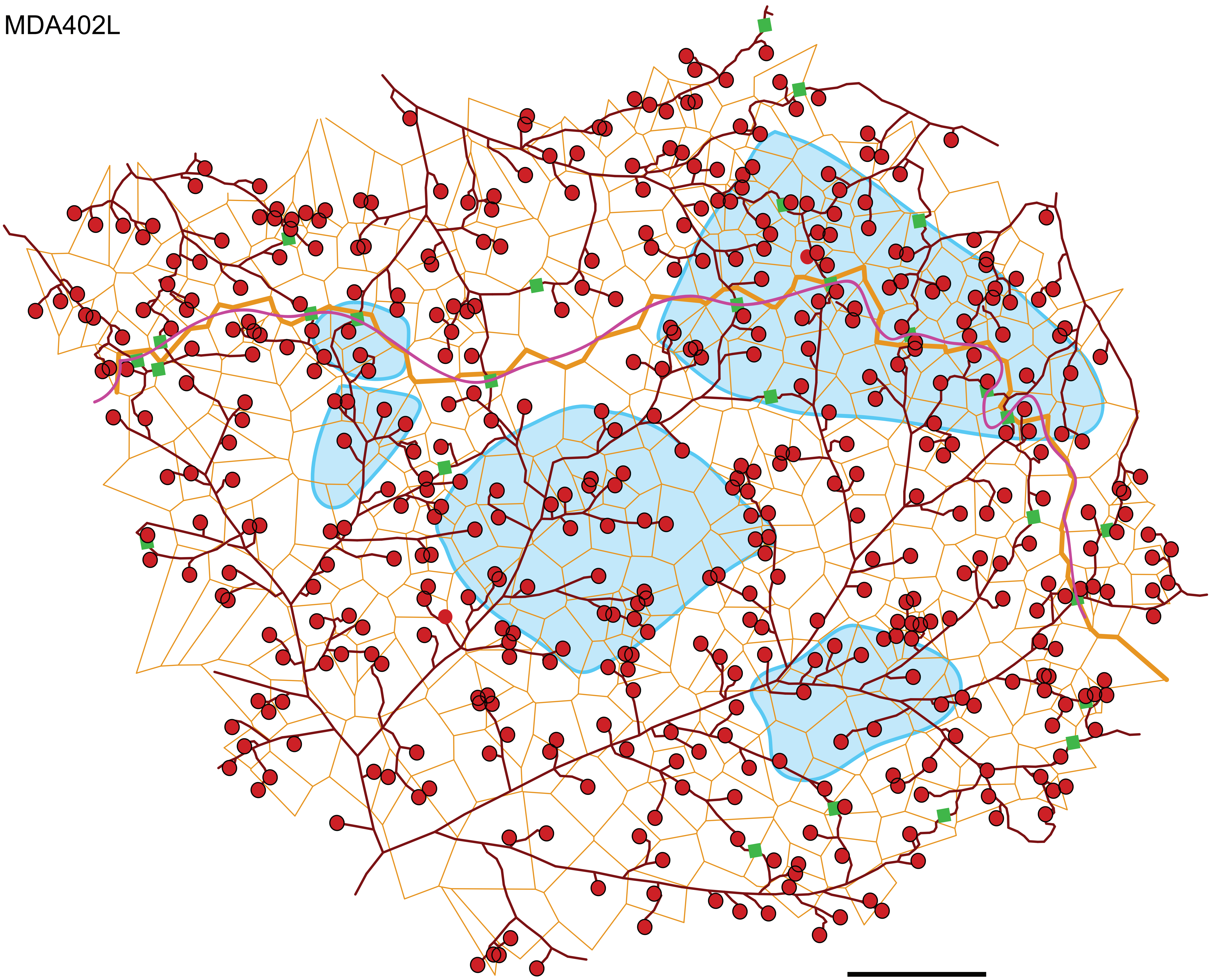
MDA402L

MDA403L

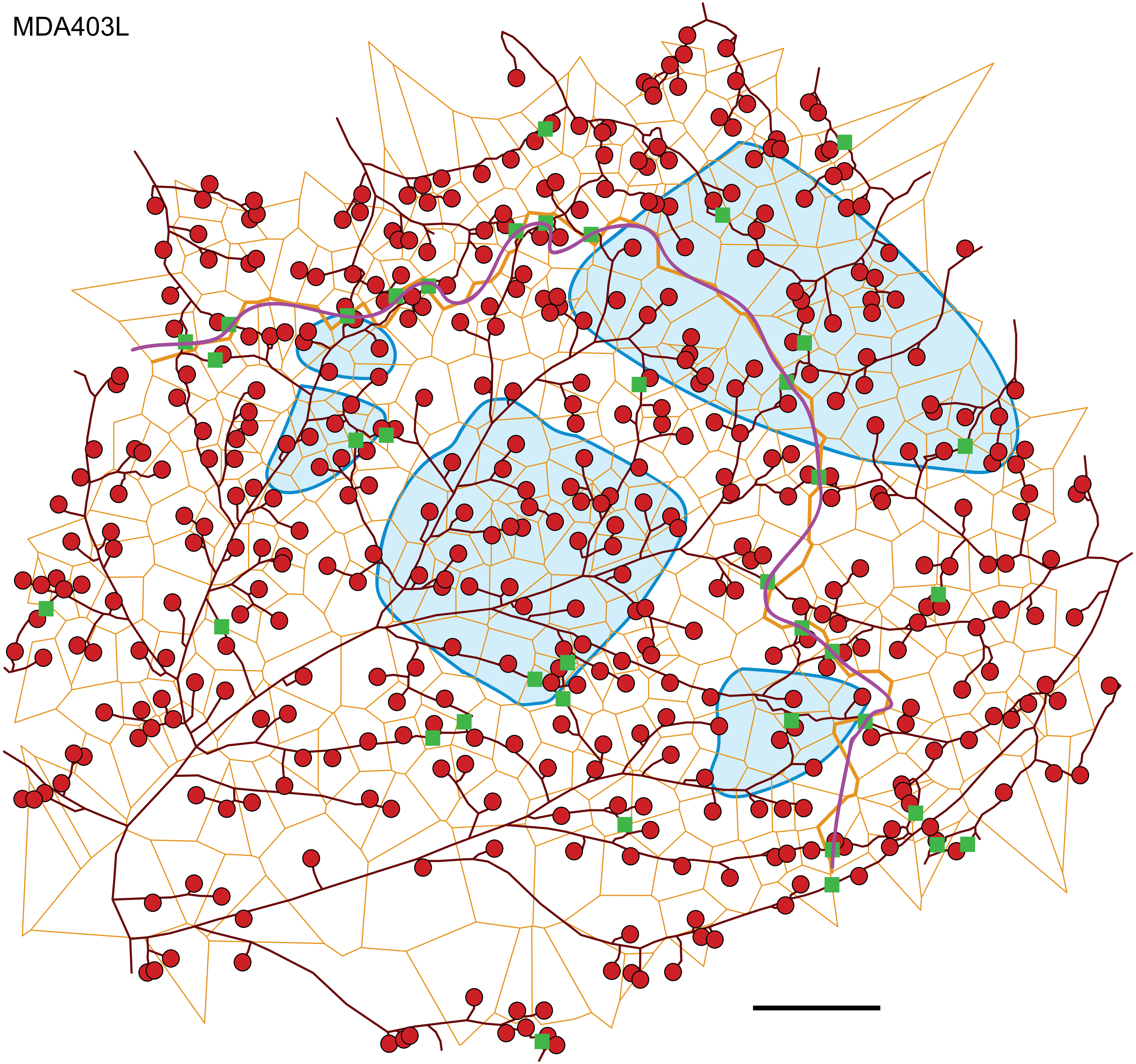

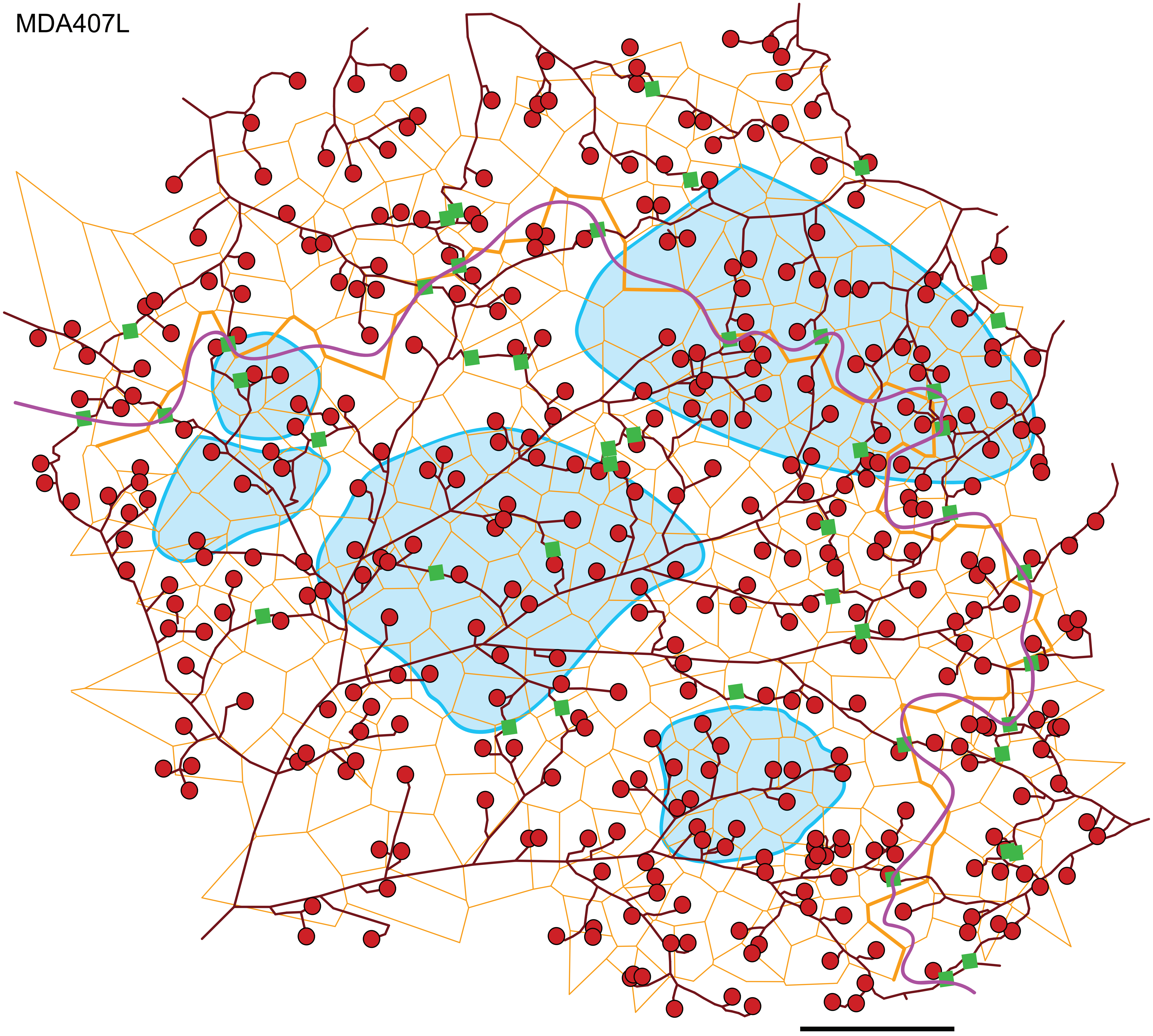
MDA407L

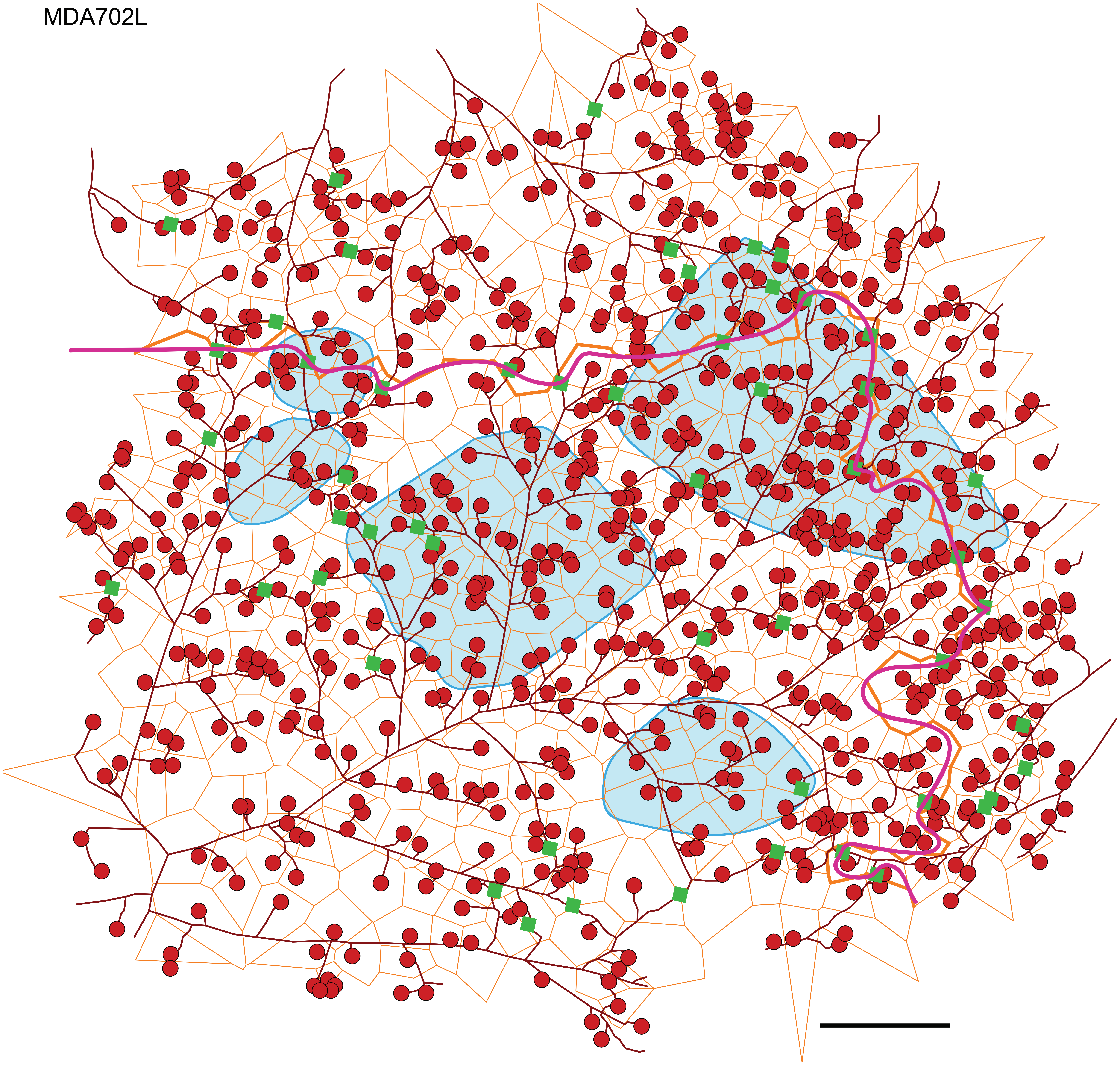
MDA702L

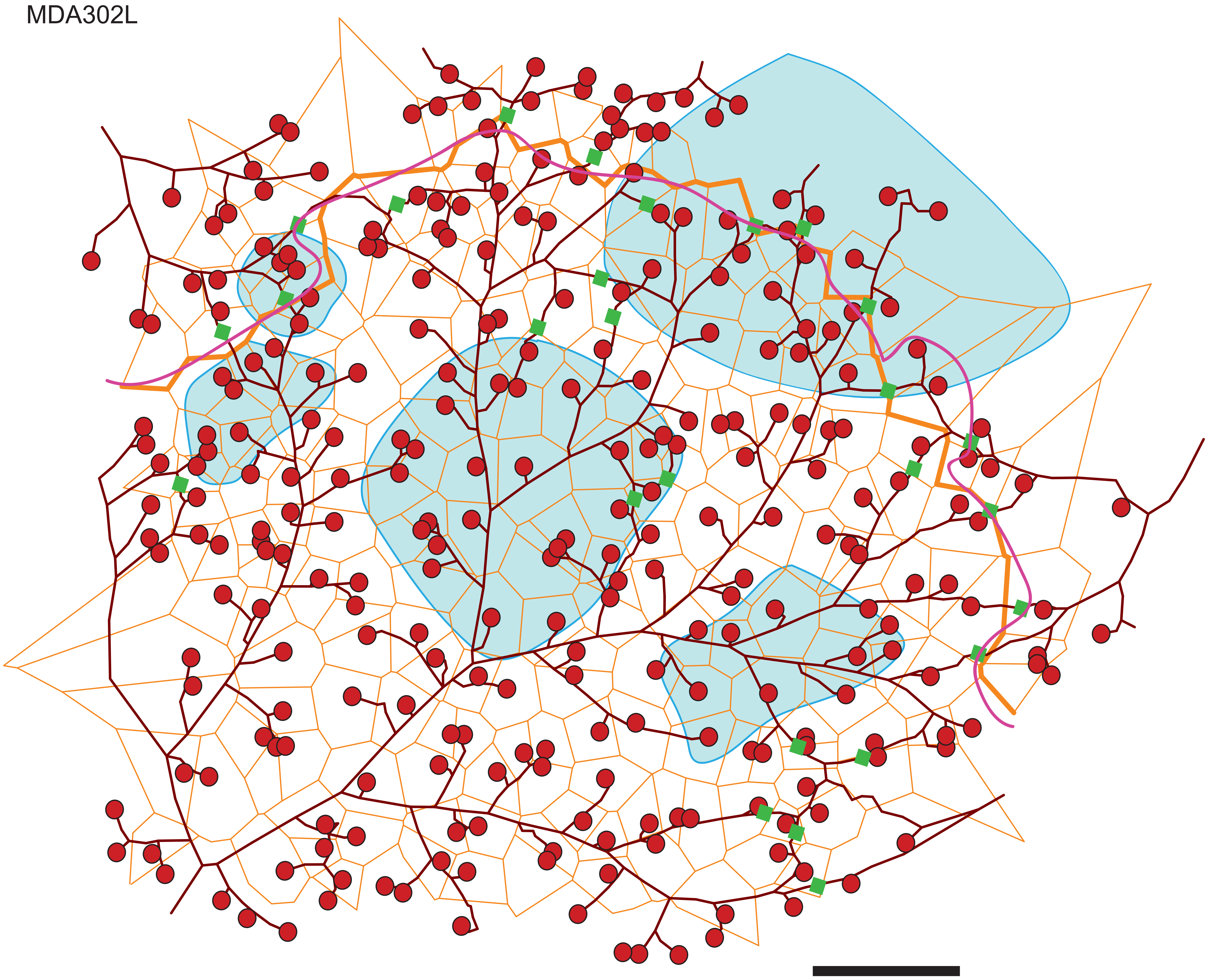
MDA302L

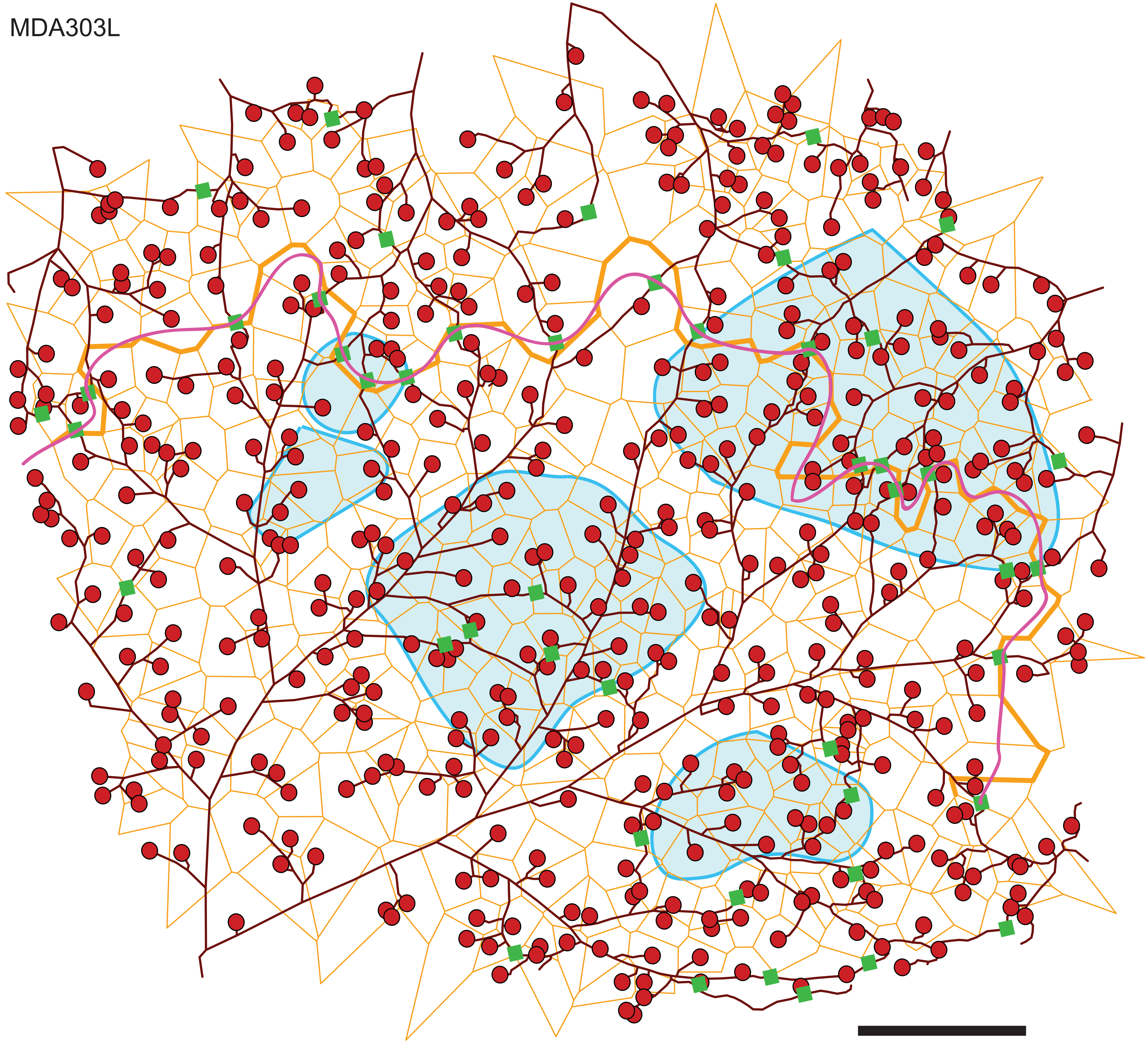
MDA303L

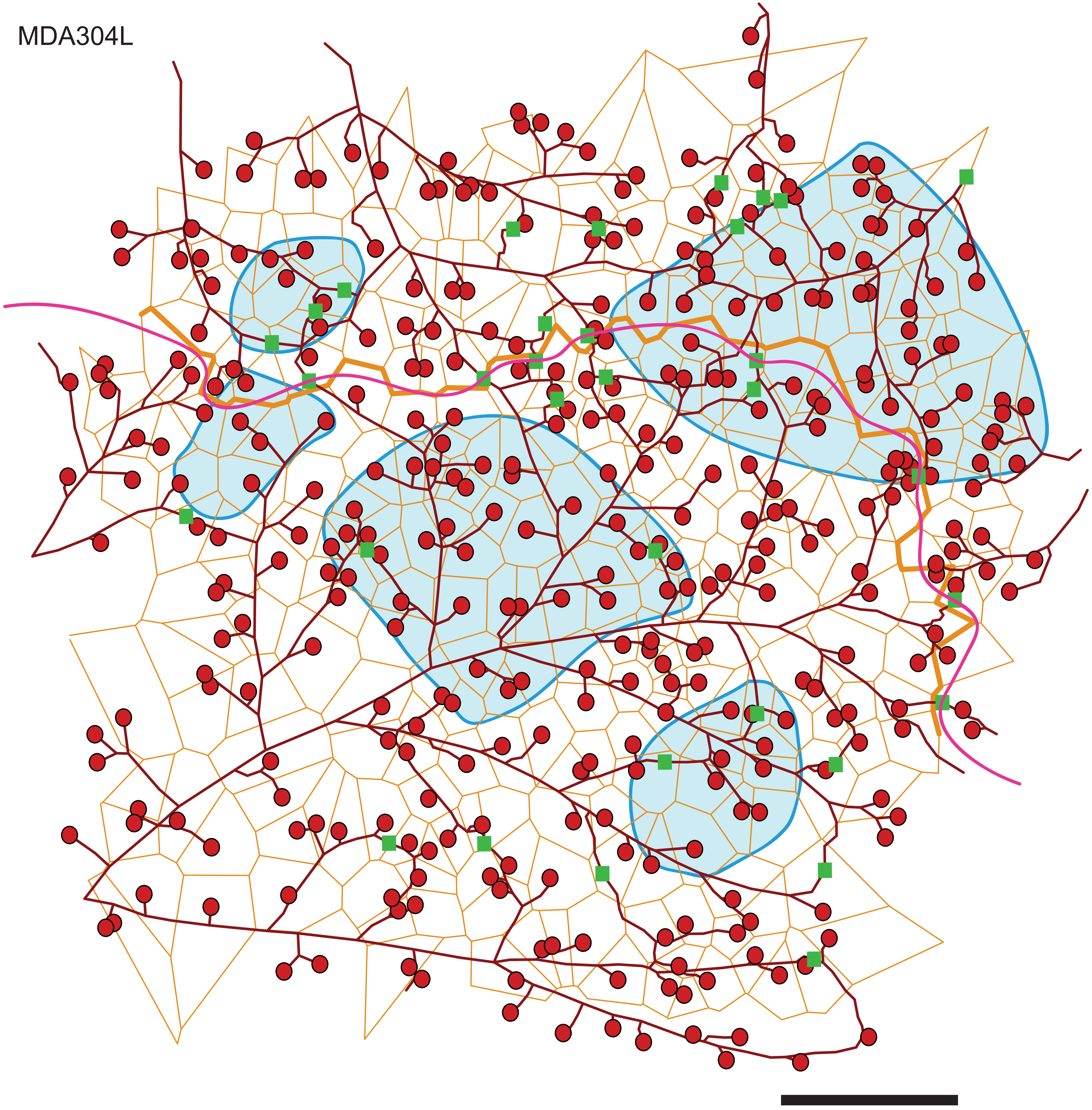
MDA304L

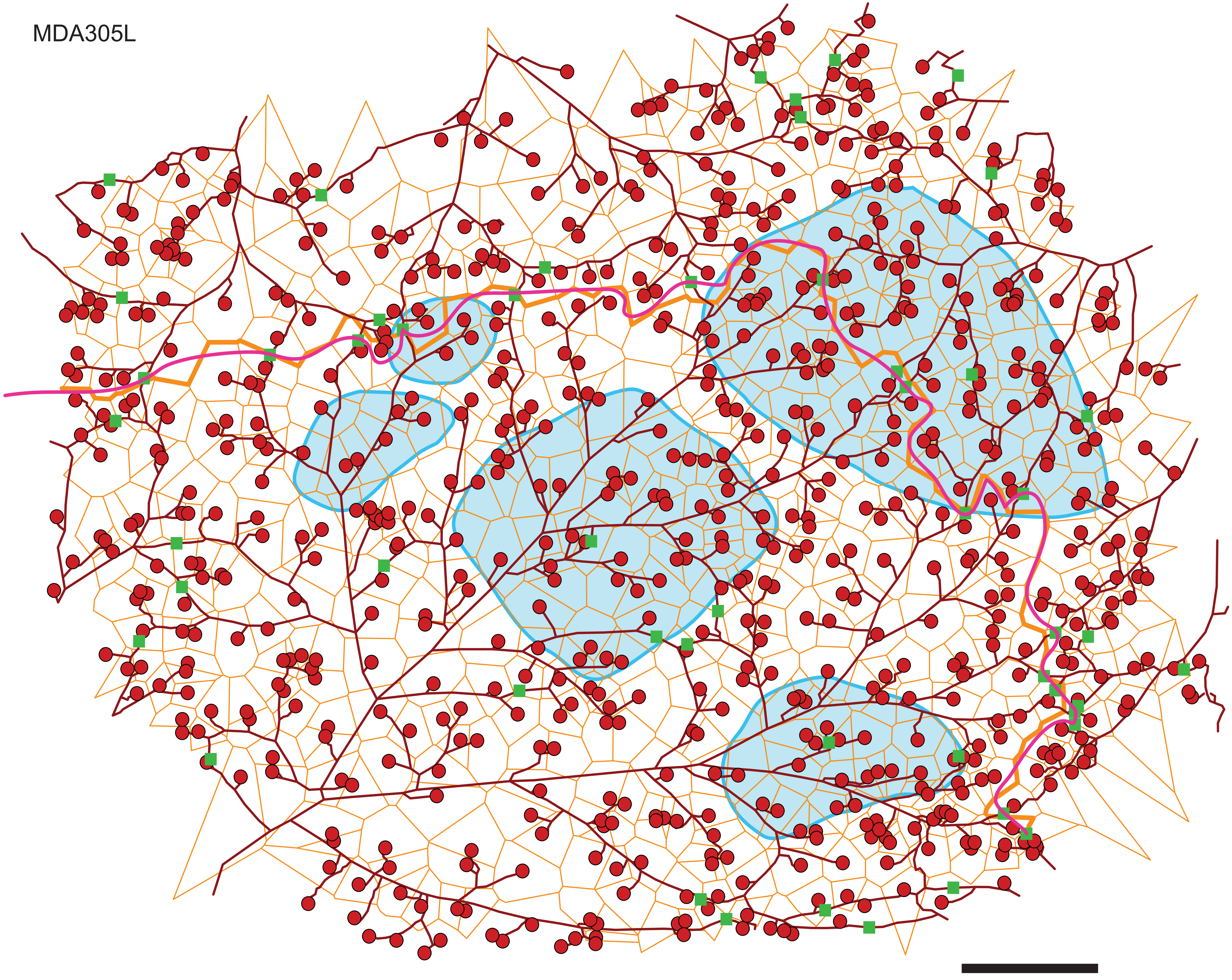
MDA305L

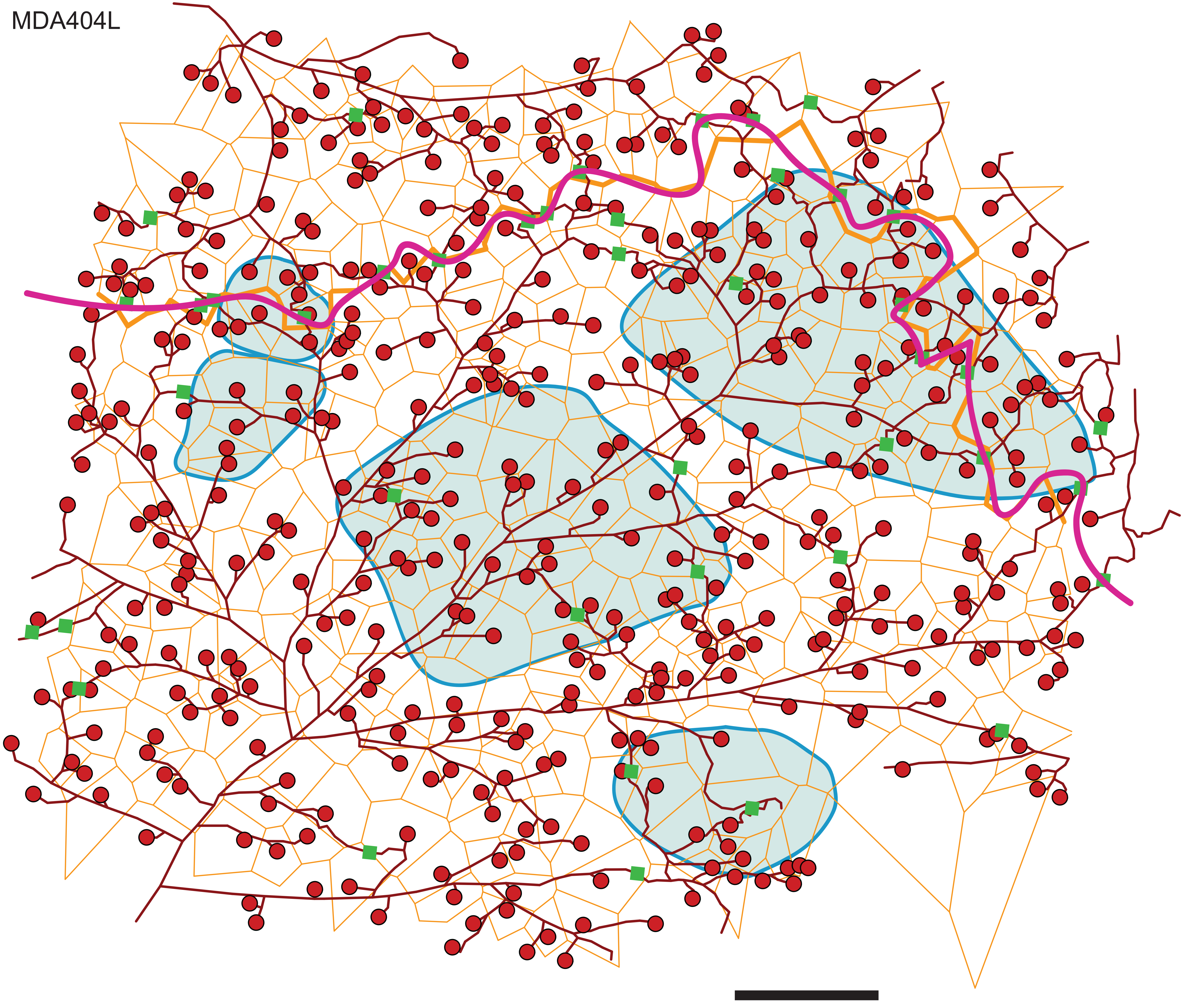

MDA404L

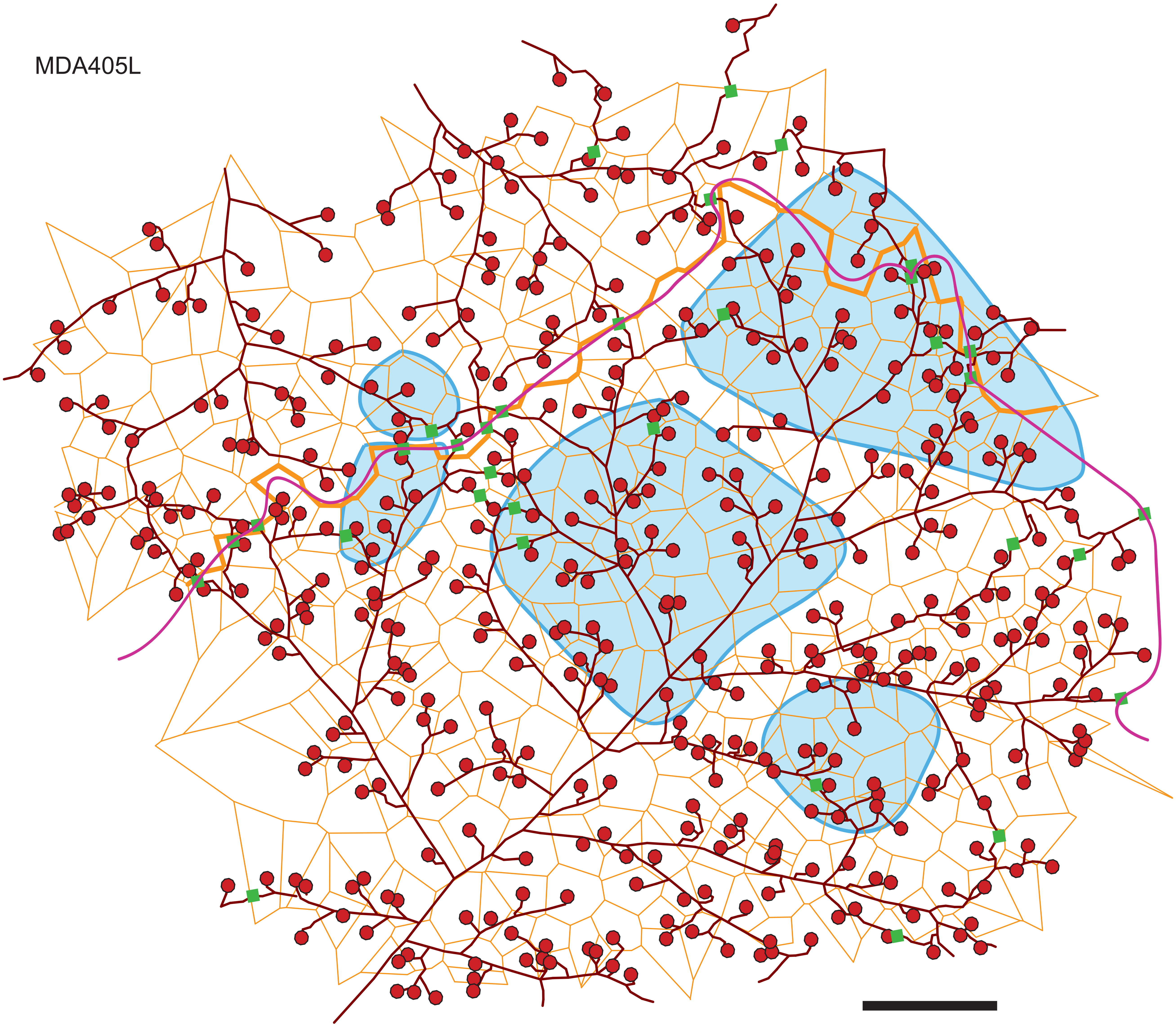
MDA405L

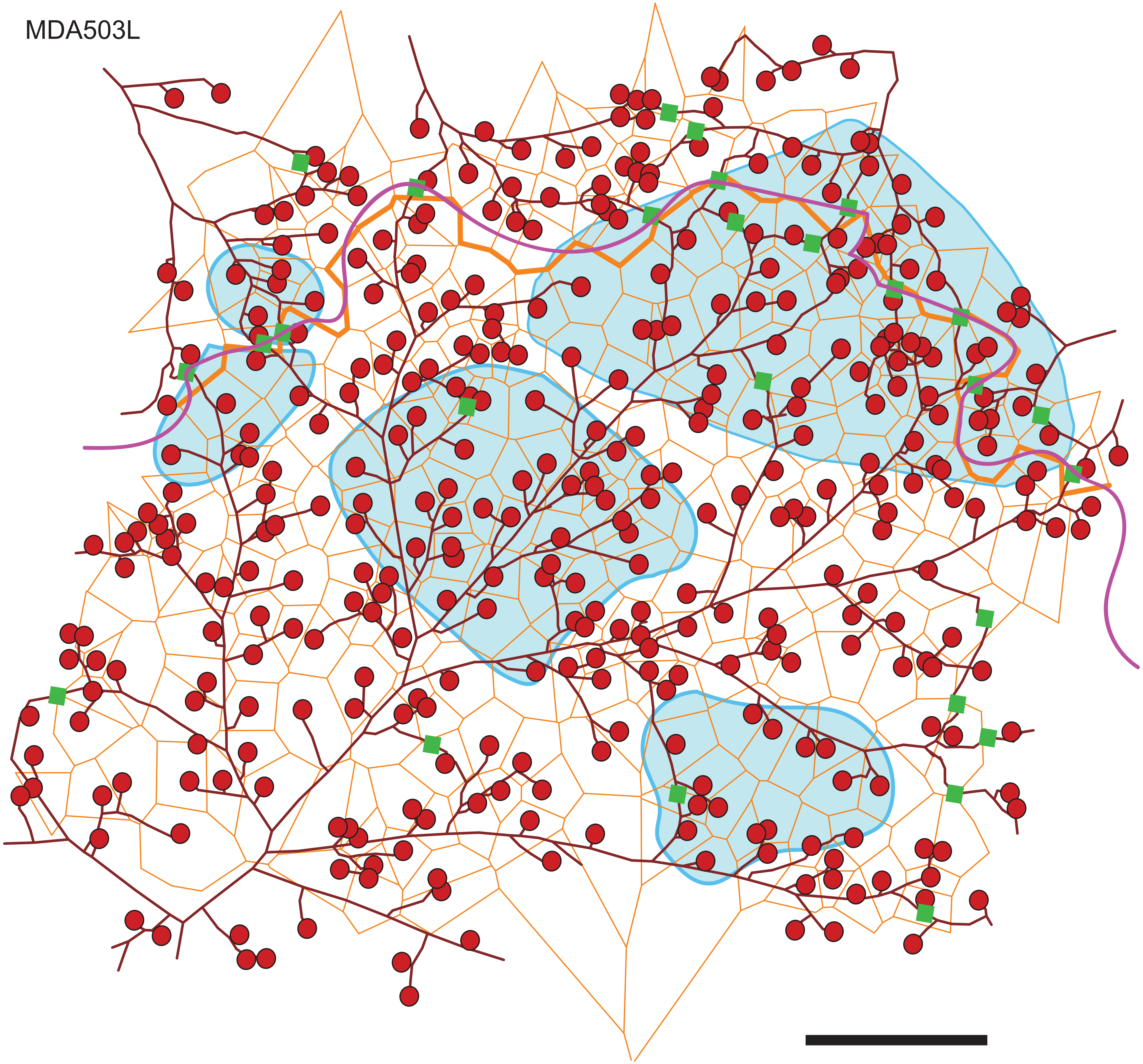
MDA503L

MDA601L

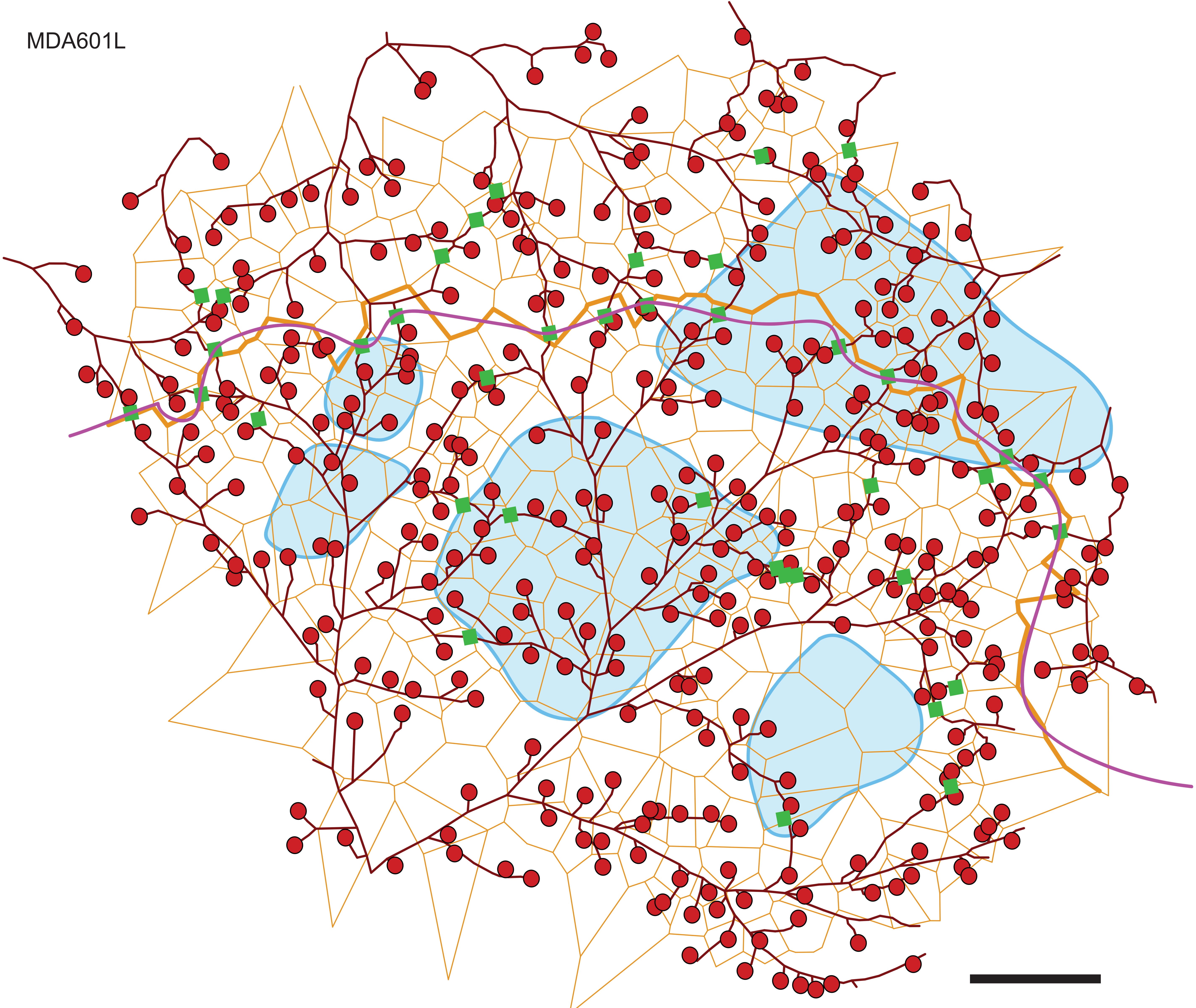

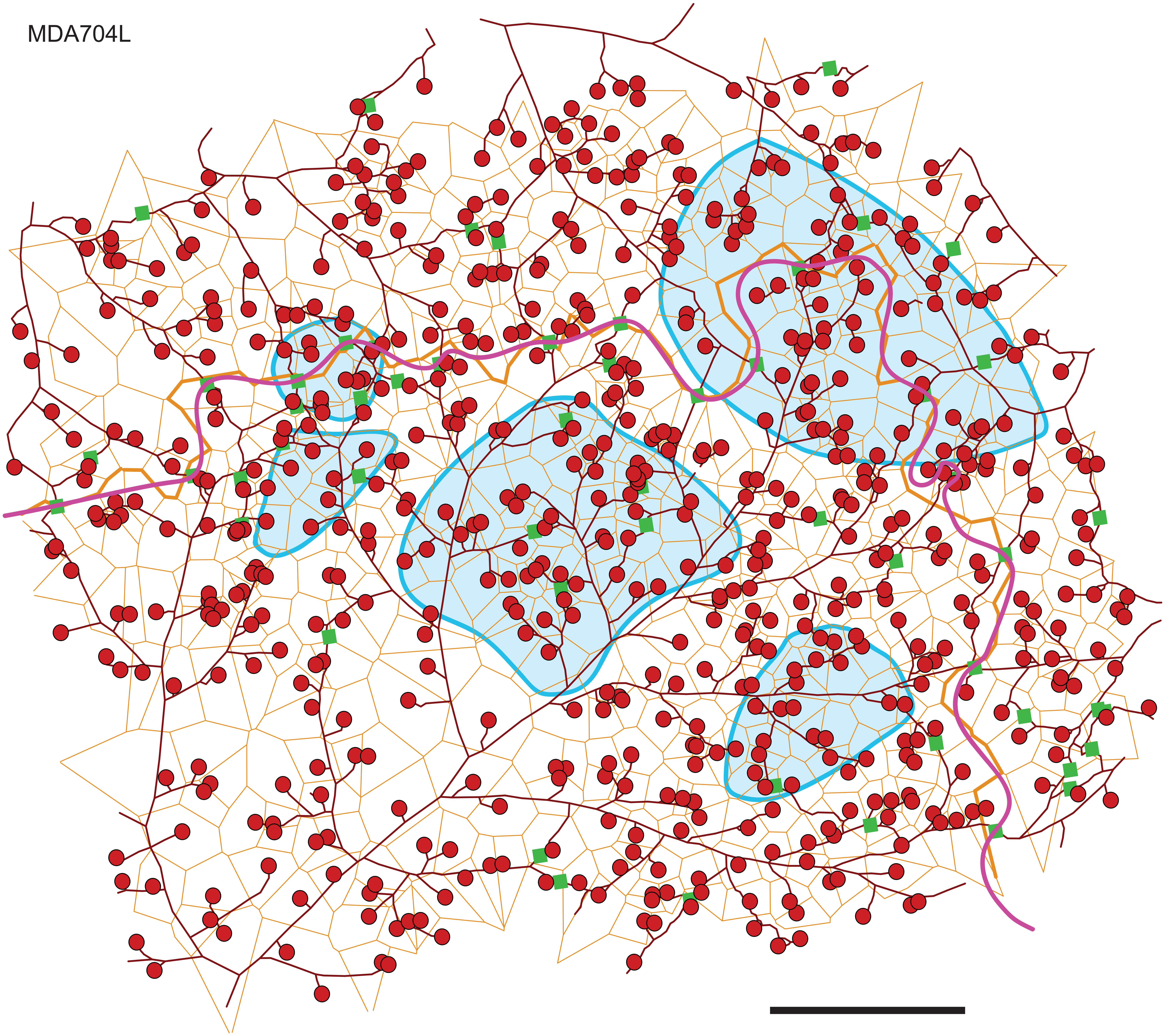
MDA704L
